# Distinct genetic pathways define pre-malignant versus compensatory clonal hematopoiesis in Shwachman-Diamond syndrome

**DOI:** 10.1038/s41467-021-21588-4

**Published:** 2021-02-26

**Authors:** Alyssa L. Kennedy, Kasiani C. Myers, James Bowman, Christopher J. Gibson, Nicholas D. Camarda, Elissa Furutani, Gwen M. Muscato, Robert H. Klein, Kaitlyn Ballotti, Shanshan Liu, Chad E. Harris, Ashley Galvin, Maggie Malsch, David Dale, John M. Gansner, Taizo A. Nakano, Alison Bertuch, Adrianna Vlachos, Jeffrey M. Lipton, Paul Castillo, James Connelly, Jane Churpek, John R. Edwards, Nobuko Hijiya, Richard H. Ho, Inga Hofmann, James N. Huang, Siobán Keel, Adam Lamble, Bonnie W. Lau, Maxim Norkin, Elliot Stieglitz, Wendy Stock, Kelly Walkovich, Steffen Boettcher, Christian Brendel, Mark D. Fleming, Stella M. Davies, Edie A. Weller, Christopher Bahl, Scott L. Carter, Akiko Shimamura, R. Coleman Lindsley

**Affiliations:** 1 Division of Hematology/Oncology, Boston Children’s Hospital, Harvard Medical School, Boston, MA USA; 2 Department of Pediatric Oncology, Dana-Farber Cancer Institute, Harvard Medical School, Boston, MA USA; 3 Department of Pediatrics, University of Cincinnati College of Medicine, Cincinnati, OH USA; 4 Division of Bone Marrow Transplantation and Immune Deficiency, Cincinnati Children’s Hospital Medical Center, Cincinnati, OH USA; 5 Institute for Protein Innovation, Boston, MA USA; 6 Department of Medical Oncology, Division of Hematological Malignancies Dana-Farber Cancer Institute, Boston, MA USA; 7 Dana-Farber Cancer Institute, Boston, MA USA; 8 Boston Children’s Hospital, Boston, MA USA; 9 Broad Institute, Boston, MA USA; 10 Biostatistics and Research Design Center, Institutional Centers for Clinical and Translational Research, Boston Children’s Hospital, Boston, MA USA; 11 Department of Internal Medicine, University of Washington, Seattle, WA USA; 12 Division of Hematology, Department of Medicine, Brigham and Women’s Hospital, Boston, MA USA; 13 Center for Cancer and Blood Disorders, Children’s Hospital Colorado, University of Colorado School of Medicine, Aurora, CO USA; 14 Department of Pediatrics/Hematology-Oncology, Baylor College of Medicine, Houston, TX USA; 15 Division of Hematology/Oncology and Cellular Therapy, Cohen Children’s Medical Center of New York, New Hyde Park, NY USA; 16 Zucker School of Medicine at Hofstra/Northwell School of Medicine, Hempstead, NY USA; 17 Shands Children’s Hospital, Department of Pediatrics, Division of Pediatric Hematology Oncology, University of Florida, Gainesville, FL USA; 18 Department of Pediatrics, Division of Pediatric Hematology Oncology, Vanderbilt University Medical Center, Nashville, TN USA; 19 Department of Medicine, Section of Hematology, Oncology, and Palliative Care, The University of Wisconsin-Madison, Madison, WI USA; 20 Indiana Blood and Marrow Transplantation, Indianapolis, IN USA; 21 Department of Pediatrics, Columbia University Medical Center, New York, NY USA; 22 Department of Pediatrics, Division of Pediatric Hematology/Oncology and BMT, University of Wisconsin, Madison, WI USA; 23 Department of Pediatrics, UCSF Benioff Children’s Hospital, San Francisco, CA USA; 24 Division of Pediatric Allergy, Immunology, and Blood & Marrow Transplantation, UCSF Benioff Children’s Hospital, San Francisco, CA USA; 25 Division of Hematology, Department of Medicine, University of Washington, Seattle, WA USA; 26 Division of Hematology-Oncology, Seattle Children’s Hospital, Seattle, WA USA; 27 Dartmouth-Hitchcock Medical Center, Pediatric Hematology Oncology, Geisel School of Medicine, Lebanon, NH USA; 28 Department of Medicine, University of Florida, Gainesville, FL USA; 29 Division of Cancer Medicine, Baptist MD Anderson Cancer Center, Jacksonville, FL USA; 30 UCSF Helen Diller Family Comprehensive Cancer Center, San Francisco, CA USA; 31 Department of Medicine, University of Chicago, Chicago, IL USA; 32 Division of Pediatric Hematology- Oncology, Department of Pediatrics, University of Michigan Medical School, Ann Arbor, MI USA; 33 Department of Medical Oncology and Hematology, University Hospital Zurich and University of Zurich, Zurich, Switzerland; 34 Harvard Stem Cell Institute, Cambridge, MA USA; 35 Department of Pathology, Boston Children’s Hospital, Boston, MA USA; 36 Joint Center for Cancer Precision Medicine, Dana-Farber Cancer Institute/Brigham and Women’s Hospital, Boston, MA USA

**Keywords:** Cancer genomics, Medical genomics

## Abstract

To understand the mechanisms that mediate germline genetic leukemia predisposition, we studied the inherited ribosomopathy Shwachman-Diamond syndrome (SDS), a bone marrow failure disorder with high risk of myeloid malignancies at an early age. To define the mechanistic basis of clonal hematopoiesis in SDS, we investigate somatic mutations acquired by patients with SDS followed longitudinally. Here we report that multiple independent somatic hematopoietic clones arise early in life, most commonly harboring heterozygous mutations in *EIF6* or *TP53*. We show that germline SBDS deficiency establishes a fitness constraint that drives selection of somatic clones via two distinct mechanisms with different clinical consequences. *EIF6* inactivation mediates a compensatory pathway with limited leukemic potential by ameliorating the underlying SDS ribosome defect and enhancing clone fitness. *TP53* mutations define a maladaptive pathway with enhanced leukemic potential by inactivating tumor suppressor checkpoints without correcting the ribosome defect. Subsequent development of leukemia was associated with acquisition of biallelic *TP53* alterations. These results mechanistically link leukemia predisposition to germline genetic constraints on cellular fitness, and provide a rational framework for clinical surveillance strategies.

## Abstract (web summary)

Understanding the molecular basis of leukaemia predisposition is essential for intervention. The authors here investigate germline genetic leukaemia predisposition by studying Shwachman-Diamond syndrome and report compensatory inactivating mutations in *EIF6* and transforming biallelic *TP53* alterations.

## Introduction

Genetic predisposition to myeloid malignancy comprises a separate disease entity in the WHO classification^1^. Diagnosis of leukemia predisposition provides potential opportunities for early intervention, but data to guide precision medicine approaches to clinical surveillance are lacking.

Shwachman-Diamond syndrome (SDS) is a genetic disorder associated with a high risk of developing myeloid neoplasms (MN) early in life^2–4^. SDS is predominantly caused by biallelic germline mutations in the *SBDS* gene^5^. The SBDS protein promotes formation of the mature, translationally active 80S ribosome by cooperating with the GTPase EFL1 to catalyze the removal of EIF6 from the 60S ribosomal subunit. In the absence of SBDS, EIF6 remains bound to the 60S subunit and sterically inhibits its joining to the 40S subunit^6^). In SDS cells, SBDS deficiency impairs eviction of EIF6 from the nascent 60S subunit, resulting in decreased ribosomal subunit joining and reduced translation efficiency^6^. Activation of cellular senescence pathways by ribosome stress incurs a global fitness defect in hematopoietic stem and progenitor cells which manifests clinically as bone marrow failure^7–9^.

Survival of patients with SDS who develop myelodysplastic syndrome (MDS) or acute myeloid leukemia (AML) is poor^10^. Therefore, a central goal in clinical care of SDS patients is to identify incipient leukemic transformation and initiate pre-emptive treatment with allogeneic stem cell transplantation. Current surveillance strategies for patients with SDS and other leukemia predisposition syndromes rely on monitoring hematologic status by serial peripheral blood counts to identify worsening cytopenias and bone marrow examinations to identify morphologic changes or development of clonal chromosomal abnormalities^11^. These tests are insensitive and detect abnormalities that are late signs of impending transformation.

The p53 tumor suppressor pathway is activated by defective ribosome biogenesis and aberrant protein translation^7,12^. Somatic *TP53* mutations have been observed in patients with SDS who develop MDS^13^, raising the possibility that next-generation sequencing could be integrated into surveillance for somatic clones with enhanced leukemia potential. However, *TP53* mutations have also been identified in SDS patients without MN^14^, suggesting that additional factors must be uncovered before implementing molecular surveillance as a predictive tool in SDS. To understand the molecular pathogenesis of MN in patients with SDS, we characterized the presence and dynamics of somatic mutations in serial, clinically annotated samples collected prospectively from patients enrolled in the North American SDS Registry and studied the functional consequences of recurrently mutated pathways.

In this work, we demonstrate using genomic and functional studies that SDS patients develop frequent somatic hematopoietic clones that either bypass or compensate for the germline defect in ribosome function, and indicate that biallelic TP53 inactivation mediates clonal transformation through checkpoint inactivation.

## Results

### Genetic pathways of somatic clonal expansion in SDS

**Fig. 1:**
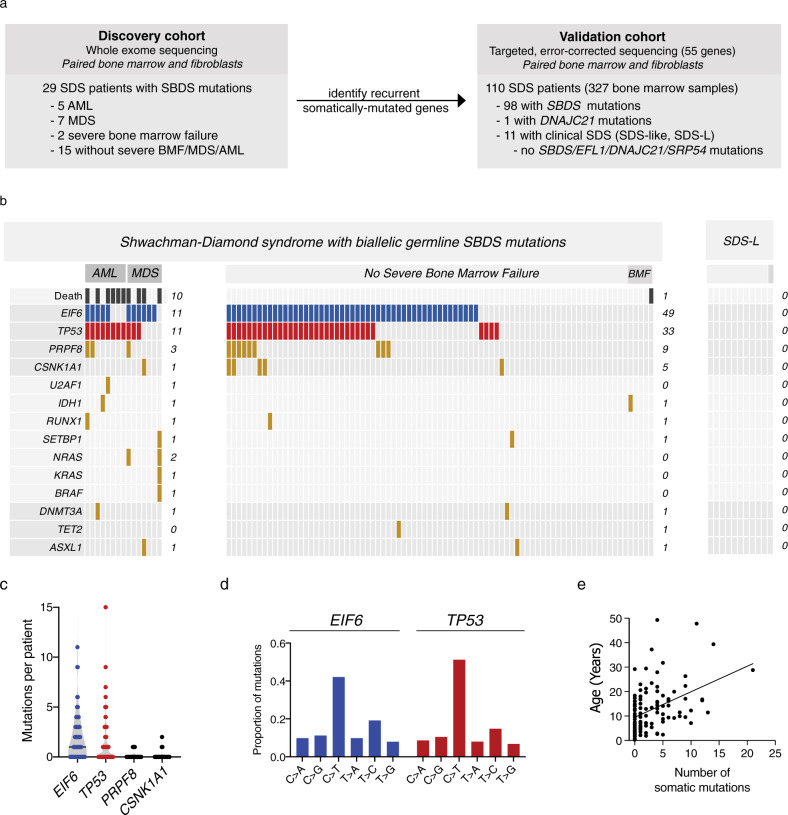
Clinical factors associated with CH in SDS patients. **a** Schema of genomic analysis. **b** A co-mutation plot showing somatic mutations in individual genes as labeled on the left. Mutations are depicted by colored bars and each column represents an individual patient in the indicated study cohort. The sum total of each event or mutation are tabulated to the right of each plot. **c** Number of mutations per patient in each of the four most frequently mutated genes:*TP53*, *EIF6*, *PRPF8*, and *CSNK1A*. **d** Base pair substitutions of somatic mutations in *TP53* and *EIF6*. **e** Total number of somatic mutations by age in patients with biallelic germline *SBDS* mutations, based on targeted deep sequencing.

We investigated genetic pathways that drive somatic hematopoietic clonal expansion and leukemogenesis in a cohort of 110 patients with a clinical diagnosis of SDS (Fig. 1a). The clinical characteristics of the cohort are described in Table 1. We first used whole exome sequencing to identify somatic mutations in bone marrow aspirate samples and paired fibroblasts from 29 patients (Fig. 1a and Supplementary Table 1). All 12 patients with MN had somatic alterations also seen in sporadic MN, including point mutations in *TP53, RUNX1*, *SETBP1, BRAF, NRAS*, and *ETNK1*, or recurrent structural alterations involving chromosomes 3, 5, 7, and 20. As expected, we observed frequent interstitial deletions of chromosome 20q (8 of 17, 47%) in patients without MN (Supplementary Fig. 1)^15^. Among these patients without MN, we further identified recurrent mutations in *EIF6* (5 of 17, 29%), suggesting that disruption of 60S:EIF6 function may drive clonal expansion in SDS cells. Since we used exome sequencing (mean target coverage 136×) for gene discovery, it is conceivable that some candidate somatic mutations were present exclusively below the threshold of exome sensitivity and were thus not taken forward to cohort-level validation.

**Table 1:** Patient characteristics.

	SDS (*n* = 99)	SDS-like (*n* = 11)
Age, median (range), years	10.8 (0.3–49.3)	15.6 (2.0–22.3)
Sex, *n* (%)		
Male	61 (61.6)	10 (90.9)
Female	38 (38.4)	1 (9.1)
SDS germline mutation, *n* (%)		
* SBDS*	98 (98.9)	0 (0)
* DNAJC21*	1 (1.0)	
Myeloid neoplasm, *n* (%)		
AML	8 (8.1)	0 (0)
MDS	7 (7.1)	0 (0)
None	84 (84.8)	11 (100)
Severe bone marrow failure, *n* (%)		
Yes	6 (6.1)	1 (9.1)
No	93 (93.9)	10 (90.9)
Granulocyte-colony-stimulating-factor, *n* (%)		
Yes	37 (37.4)	0 (0)
No	55 (55.6)	1 (9.1)
Not available	7 (7.1)	10 (90.9)

We performed targeted validation of candidate gene mutations in paired bone marrow and fibroblast samples from the whole cohort, including samples in the exome cohort. We sequenced 55 genes, including those recurrently mutated in the discovery exome cohort, as well as genes associated with sporadic MN (Supplementary Table 2). To detect clones present at low abundance [0.1% variant allele fraction (VAF)], we used a platform that incorporated duplex unique molecular identifiers, thereby enabling computational suppression of sequencing artifacts.

We initially focused our analysis on the most recent sample from each patient. We detected 327 somatic mutations in 74 of 98 (76%) SDS patients with germline *SBDS* mutations (median 2 mutations/patient, range 0–21), and no mutations in patients with SDS-like (SDS-L) disease who have some clinical features of SDS without disease defining mutations (*SBDS*, *EFL1*, *DNAJC21*, *SRP54*). The most frequent somatically mutated genes were *EIF6* (60/98, 61%), *TP53* (44/98, 45%), *PRPF8* (12/98, 12%), and *CSNK1A1* (6/98, 6%) (Fig. 1b). Secondary somatic *SBDS* mutations were found in three patients and no other genes were mutated in more than two patients. Among 74 patients with somatic mutations, 52 (72.2%) had multiple mutations, frequently affecting the same gene (Fig. 1c). The most common base substitution in somatic *EIF6* and *TP53* variants was a cytosine-to-thymine (C → T) transition (Fig. 1d), which is the predominant mutational signature associated with normal aging hematopoietic stem cells (HSCs), sporadic clonal hematopoiesis (CH), and AML^16–19^.

### Clinical factors associated with somatic mutations

Detectable *TP53* mutations were more common in SDS patients with germline *SBDS* mutations and MN than in those without MN (73.3% versus 39.8%, *p* = 0.023), while *EIF6*, *CSNK1A1*, and *PRPF8* were not associated with MN. In univariate analysis, the presence of any somatic mutation was associated with older age (median 12.9 versus 4.7 years, *p* = 0.0001), as were mutations in individual genes (*TP53*, *p* = 0.0002; *EIF6*, *p* = 0.0042; *PRPF8*, *p* = 0.0461) (Fig. 1e). Logistic regression adjusting for age, sex, and the presence of MN showed that age was independently associated with the presence of any somatic mutation (OR = 1.1, for each one-year increase in age, 95% CI 1.1–1.2, *p* = 0.0017). Further, the total number of somatic mutations per patient was positively associated with age and MN [*β*(se) = 0.50 (0.2071), *p* = 0.0165] in a Poisson regression model adjusted for the same variables.

### *EIF6* mutations are highly recurrent and specific to SDS

**Fig. 2:**
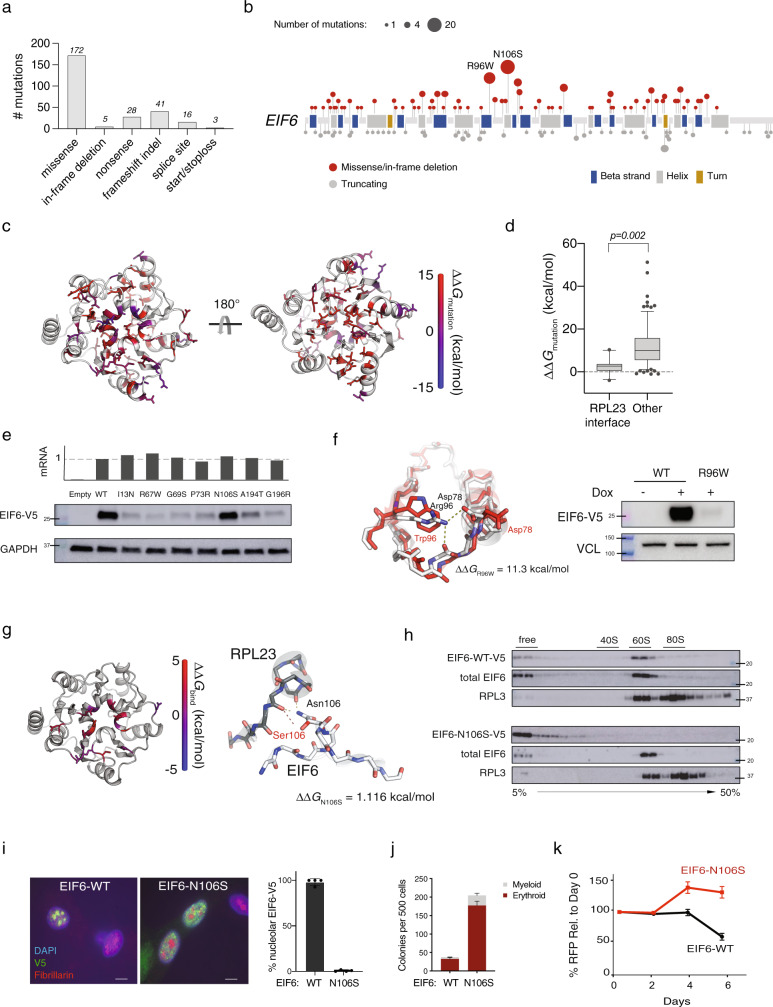
*EIF6* somatic missense mutations alter EIF6 protein stability or function to improve cell fitness. **a** Types of somatic *EIF6* mutations. **b** Number and location of *EIF6* mutations according to variant type. **c** Impact on the calculated energy of the folded state (ΔΔ*G*_mutation_) of *EIF6* missense mutations. Mutant residue colored according to ΔΔ*G* value. **d** ΔΔ*G*_mutation_ of 12 *EIF6* missense mutations located at the RPL23-binding interface versus 85 other *EIF6* missense mutations located in the remainder of *EIF6*. Boxes center around the median and span the 25th and 75th percentiles, whiskers extend to the 10th and 90th percentiles. *p* value calculated using unpaired two tailed *t*-test. **e** Relative levels of EIF6 mRNA using a V5-specific qPCR primer (top panel) and V5 immunoblot from K562 cells 48 h after doxycycline treatment (bottom panel). Data shown is representative of three independent experiments. **f** Left panel: In silico modeling of EIF6-R96W. Right panel: V5 and VCL immunoblots of K562 cells with inducible EIF6-R96W versus V5-wild type EIF6 48 h after doxycycline treatment. Data shown is representative of three independent experiments. **g** Left panel: Change in the energy of binding (ΔΔ*G*_bind_) of missense mutations at RPL23 interface. Mutant residues are colored according to ΔΔ*G*_bind_. Right panel: In silico modeling of EIF6 N106S mutation. **h** V5, EIF6, and RPL3 immunoblots of sucrose gradient fractions from polysome profiles of doxycycline-treated K562 cells expressing V5-EIF6-WT or V5-EIF6-N106S. Data shown is representative of three independent experiments. **i** Immunofluorescence of V5-EIF-WT or V5-N106S-EIF6 protein in SDS patient-derived fibroblasts, V5 (green), fibrillarin (red), and DAPI (blue). Right panel: quantification of V5 nucleolar signal from four independent experiments. Error bars represent the mean ± standard deviation. Scale bar = 10 μm. **j** Quantification of colony forming units from sorted CD34+ transduced with shSBDS-GFP and either EIF6-WT-RFP or EIF6-N106S-RFP plated in triplicate. Data shown are representative of three independent experiments. Error bars represent the mean ± standard deviation. **k** Competitive growth of doxycycline inducible-shSBDS in K562 cells (Supplementary Fig. 3D) transduced with either EIF6-WT-RFP or EIF6-N106S-RFP after indicated time from doxycycline treatment (*n* = 3 technical replicates, representative of three biological replicates). Error bars represent mean ± standard deviation.

Across all samples, we identified 265 *EIF6* mutations (Fig. 2a), all of which were in patients with germline *SBDS* mutations. We did not detect any *EIF6* mutations in control cohorts, including patients with SDS-L disease (*n* = 11), patients with other leukemia predisposition disorders (germline *GATA2* deficiency syndrome, *n* = 32; telomere biology disorders, *n* = 5; germline *SAMD9*/*SAMD9L* mutations, *n* = 5), or adults with sporadic AML (*n* = 39). *EIF6* truncating mutations were distributed throughout the coding region, whereas missense mutations were predominantly located in regions encoding conserved secondary structure (Fig. 2b).

To study the consequences of *EIF6* missense mutations, we generated a homology model that closely matches the EIF6 structures from *Methanocaldococcus jannaschii*^20^, *Saccharomyces cerevisiae*^20,21^, and *Dictyostelium discoideum*^22^ (Supplementary Fig. 2). To evaluate the impact of mutations on protein stability, we modeled the effect of each mutation on the energy of the folded state of EIF6 and compared this to the wild-type protein (ΔΔ*G*_mutation_) (Fig. 2c). Missense mutations located at the EIF6:RPL23 interface were not predicted to destabilize the protein (median ΔΔ*G*_mutation_ = 2.66 kcal/mol, 95% CI 0.29–3.73) (Fig. 2d and Supplementary Table 3). In contrast, mutations not located at binding interfaces (median ΔΔ*G*_mutation_ = 9.83 kcal/mol, 95% CI 7.37–12.37) and those located at the non-60S interface with EFL1 (median ΔΔ*G*_mutation_ = 10.04 kcal/mol, 95% CI 5.79–24.93) (Fig. 2d) were strongly destabilizing.

### *EIF6* mutations disrupt 60S:EIF6 function by two mechanisms

We cloned patient-derived mutations with different predicted functional consequences and generated K562 leukemia cell lines that expressed wild-type or mutant *EIF6* cDNA with a C-terminal V5-epitope tag under the control of a doxycycline-inducible promoter. We measured EIF6 protein levels and mRNA expression after 48 h of doxycycline treatment and found that six mutants (I13N, R67W, G69S, P73R, A194T, G196R) had reduced levels of EIF6 protein compared with EIF6^WT^, despite comparable abundance of mutant mRNA (Fig. 2e). Mutant EIF6 protein abundance was increased after treatment with the proteasome inhibitor MG-132 (Supplementary Fig. 3A). To assess the effect of destabilizing *EIF6* mutations on the functional competency of SDS hematopoietic stem and progenitor cells, we expressed either EIF6^WT^, EIF6^I13N^, or EIF6^A194T^ in SBDS-deficient human CD34+ cells and quantified hematopoietic colony formation. Both myeloid and erythroid colonies were more abundant with EIF6^I13N^ and EIF6^A194T^ compared with EIF6^WT^ (Supplementary Fig. 3B). These results indicate that *EIF6* missense mutations can cause functional inactivation via protein destabilization.

The two most common recurrent mutations in the cohort were *EIF6* p.N106S and *EIF6* p.R96W, found in 20% and 13% of SDS patients, respectively. Among patients with somatic *EIF6* mutations, p.N106S was found in 32% and *EIF6* p.R96W in 22%. In the EIF6 homology model, R96W disrupts hydrogen bonds and is predicted to destabilize the protein (ΔΔ*G*_mutation_ = 11.3 kcal/mol), while N106S is predicted to be stable (ΔΔ*G*_mutation_ = 1.12 kcal/mol). Consistent with these models, the level of EIF6^R96W^ protein was markedly reduced compared with EIF6^WT^ (Fig. 2f) and the level of EIF6^N106S^ protein was similar to EIF6^WT^ (Fig. 2e).

Since N106 is highly conserved and located at the interface between EIF6 and the 60S ribosomal protein RPL23 (Fig. 2g), we tested the hypothesis that N106S impairs the EIF6:60S interaction. In the homology model, N106S had significantly increased energy of EIF6:RPL23 binding (ΔΔ*G*_bind_)^23^ compared with mutations not located at the EIF6:RPL23 interface. To directly analyze the impact of N106S on this interaction, we conducted sucrose gradient polysome profiling of lysates from cells expressing V5-tagged EIF6^WT^ or EIF6^N106S^, followed by western blotting across the gradient fractions. While EIF6^WT^ was primarily present in the 60S fractions^24,25^, EIF6^N106S^ was found only in the free fractions and was absent from the 60S fractions (Fig. 2h). To establish whether lack of EIF6^N106S^ binding to 60S altered protein synthesis, we compared EIF6^N106S^ to EIF6^WT^ and found that EIF6^N106S^ improved the SDS-associated impairment of translation as measured by incorporation of O-propargyl-puromycin into nascent peptides (Supplementary Fig. 3C). Additionally, EIF6^WT^ was distributed normally in the cytoplasm and nucleolus^21,26^ and EIF6^N106S^ was detectable only in the cytoplasm (Fig. 2i).

To assess the effect of EIF6^N106S^ on colony formation, we expressed either EIF6^WT^ or EIF6^N106S^ in SBDS-deficient human CD34+ cells. Comparable to destabilizing mutants, we observed an increase in the number of myeloid and erythroid colonies with EIF6^N106S^ compared with EIF6^WT^ (Fig. 2j). Similarly, SBDS-deficient cells expressing EIF6-N106S displayed increased overall growth compared to cells expressing EIF6^WT^ (Fig. 2k).

### *EIF6* and *TP53* mutations alleviate p53 activation

SBDS deficiency impairs ribosome assembly and results in reduced abundance of the mature 80S ribosome, concomitant accumulation of free 60S ribosome subunits^27,28^, and upregulation of p53-dependent cellular stress pathways in SDS patient bone marrow^29^ and SDS mouse models^8^. Somatic mutations that reduce p53 activation could thus drive selective clonal advantage either by rescuing the underlying defect in ribosome maturation or by directly inactivating *TP53*.

**Fig. 3:**
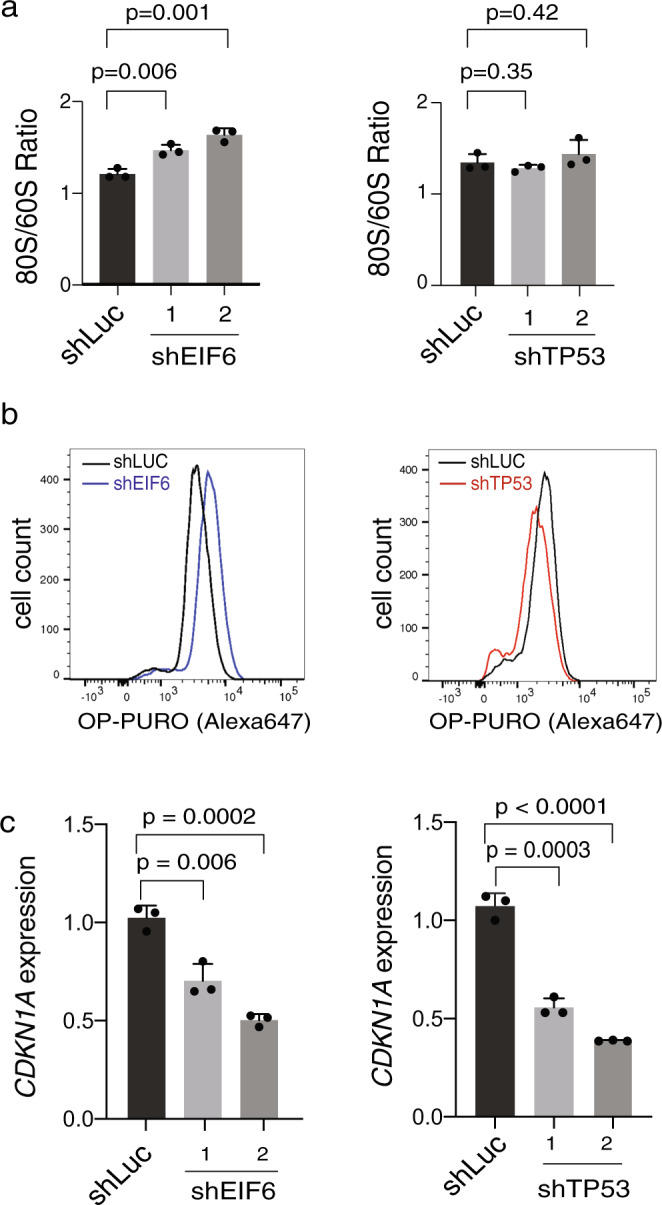
*EIF6* and *TP53* mutations attenuate p53 activation via different mechanisms. **a** Quantification of 80S:60S ratio from polysome profiles in SDS patient-derived primary fibroblasts transduced with shRNAs targeting luciferase, EIF6 (left panel) or TP53 (right panel). **b** OP-Puro incorporation in primary SDS patient-derived fibroblasts transduced with shRNAs targeting luciferase, EIF6 (left panel) or TP53 (right panel). **c** Relative *CDKN1A* expression in SDS patient-derived fibroblasts transduced with either shLUC control or shEIF6 (left panel) and shTP53 (right panel). Error bars represent mean ± standard deviation of three technical replicates representative of two to three independent experiments. *p* value calculated using unpaired two-tailed *t*-test.

To investigate the effects of *EIF6* and *TP53* mutations on ribosome maturation, protein translation, and p53 target gene activation in SBDS-deficient cells, we introduced shRNAs that targeted *EIF6* or *TP53*, or a control shRNA targeting luciferase into primary SDS patient-derived bone marrow fibroblasts (Supplementary Fig. 4A). Using sucrose gradient polysome profiling, we found that knockdown of *EIF6*, but not knockdown of *TP53*, resulted in an increased ratio of 80S:60S ribosomal subunits relative to control (Fig. 3a and Supplementary Fig. 4B). Consistent with these distinct effects on ribosome maturation, knockdown of *EIF6*, but not knockdown of *TP53*, improved the SDS-associated impairment of protein synthesis, as measured by incorporation of O-propargyl-puromycin into nascent peptides (Fig. 3b and Supplementary Fig. 4C). Despite their different impact on the SDS ribosome joining defect, knockdown of either *EIF6* or *TP53* resulted in reduction of *CDKN1A* induction in SDS fibroblasts (Fig. 3c). Together, these results indicate that *EIF6* and *TP53* mutations have distinct effects on ribosome joining and global protein synthesis, but share a common downstream effect of reducing *CDKN1A* expression, which is a marker of p53 pathway activation.

### Independence of *TP53* and *EIF6* mutated clones in SDS

Among SDS patients with *TP53*-mutated CH, 90.9% (30 of 33) had concurrent *EIF6* mutations, raising the possibility that *TP53* and *EIF6* mutations cooperate to drive clonal progression. To distinguish whether *TP53* and *EIF6* mutations arise in separate clones or together within the same clones, we performed single cell DNA sequencing from patients with CH who had multiple *EIF6* and *TP53* mutations detected by bulk sequencing.

**Fig. 4:**
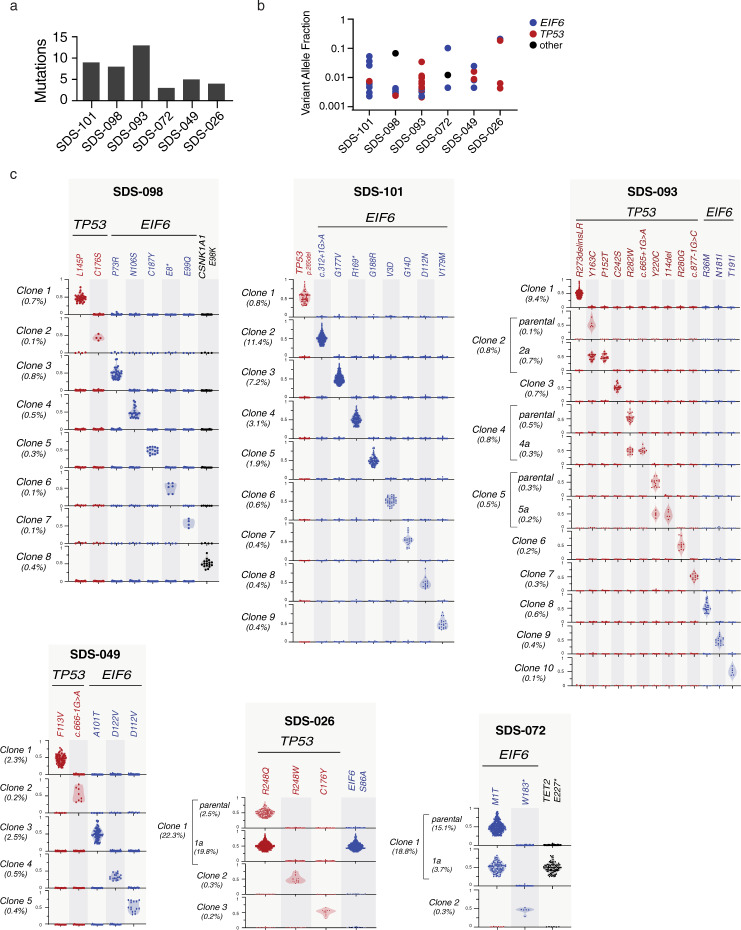
Independence of *TP53* and *EIF6* mutated clones in SDS patients. **a** Number of somatic mutations detected in each patient by bulk DNA sequencing. **b** corresponding VAF of *TP53* (red), *EIF6* (blue) or other (black) mutation. **c** Clonal hierarchy of mutations determined by single cell sequencing amongst six patients with SDS. Each row represents a unique clone or subclone and the frequency of each clone is indicated to the left. Columns reflect the genotype status of each mutation in each clone, and all depicted clones have complete genotyping at all loci. The *y*-axis indicates single cell VAF from 0 to 1, where 0 is absent, 0.5 is heterozygous mutation, and 1 is homo/hemizygous. Each dot reflects a single cell, colored according to gene mutation, *TP53* (red), *EIF6* (blue), *CSNK1A1* (black) and the frequency distribution of the data points reflected by shaded violin plots.

Using a custom panel covering seven genes implicated in SDS or sporadic CH and 43 single nucleotide polymorphism (SNP) loci on chromosomes 7, 17, and 20, we sequenced 33,426 cells from six patients with CH. The number (Fig. 4a) and VAF (Fig. 4b) of gene mutations detected by bulk sequencing in each patient is shown in Fig. 4. Single cell data was analyzed using the Mission Bio Tapestri Insight platform and mean sample level allelic dropout (ADO) was 10.1% (95% CI 8.3–11.8%). ADO was not accounted for computationally during clone identification, but was taken into account during manual review based on metrics including genotype quality, read depth relative to parent clone, and overall clone size. We focused the single cell analysis only on mutations that were detected using our bulk DNA sequencing platform; we did not make any de novo variant calls from the scDNA-sequencing data. Genotyping was successful for 84.4% of all targeted mutations that were observed by bulk DNA sequencing and undetected mutations were restricted to low abundance clones (median VAF 0.0032, range 0.0022–0.0087). Using this single cell approach, we found that somatic mutations were almost always present in independent clones: among the 50 clones we identified: 24 had a sole *EIF6* mutation, 21 had a sole *TP53* mutation, and 3 had a sole *CSNK1A1* mutation (Fig. 4c). One patient (SDS-026) had a clone with concurrent mutations in *TP53* and *EIF6*, where *TP53* p.R248Q defined the founding clone and *EIF6* p.S86A defined a subclone. In another patient (SDS-072), we observed a founding clone with *EIF6* p.M1T and a subclone with *TET2* p.E227* mutation.

### Clonal hematopoiesis in SDS patients

**Fig. 5:**
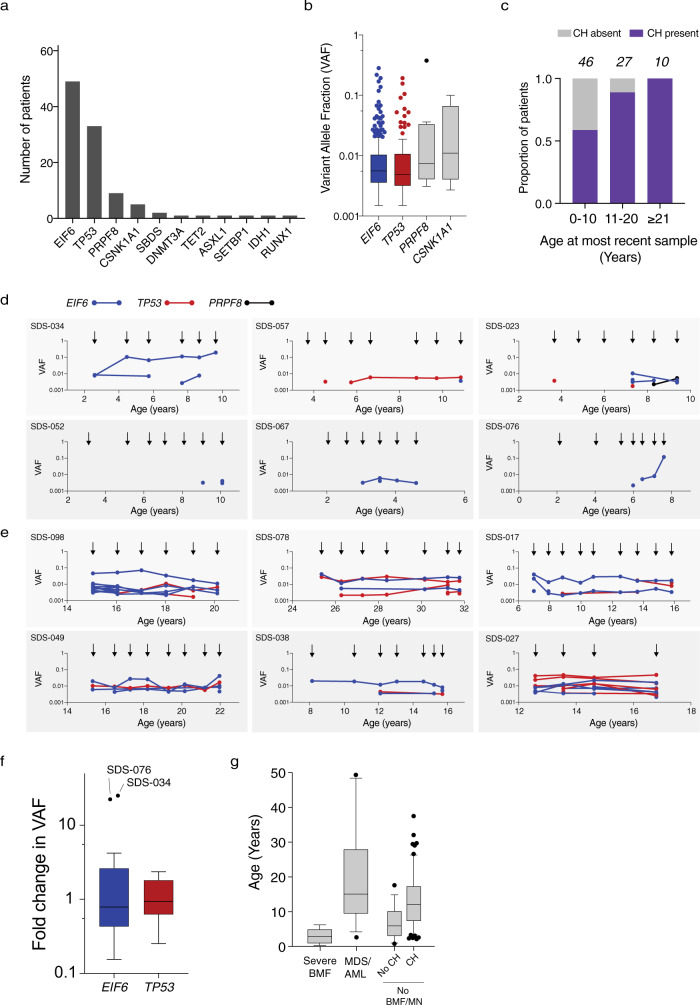
CH in SDS patients. **a** Frequency of mutations in the indicated genes among the 58 SDS patients with CH. **b** VAFs in the indicated genes among 378 samples from SDS patients with clonal hematopoiesis. Horizontal lines within boxes indicate median VAF. Boxes center around the median and span the 25th and 75th percentiles with whiskers and outliers defined by the Tukey method. **c** Proportion of patients in the study cohort per decade of age with detectable CH, where CH was defined as the presence of a recurrent somatic clonal genetic alteration. **d**, **e** Shown is the VAF of each somatic *EIF6* (blue), *TP53* (red) or *CSNK1A* mutation (black) from **d** six patients who developed clonal hematopoiesis in the first decade of life and **e** six patients who were found to have clonal hematopoiesis in their second or third decade of life. Arrows indicate timing of sample acquisition. Points represent the VAF for detected mutations **f**, Fold change in VAF of all somatic *EIF6* (blue) and *TP53* (red) mutations from time of first detection to time of most recent detection in 23 patients with CH. Boxes center around the median and span 25th and 75th percentiles with whiskers and outliers defined by the Tukey method. **g** Ages of patients at diagnosis of six patients with severe bone marrow failure (BMF), 15 patients with myeloid neoplasm (MN), or no BMF/MDS with (59 patients) or without CH (19 patients) at last follow up. Boxes center around the median and span the 25th and 75th percentiles, whiskers extend to the 10th and 90th percentiles.

CH in individuals without germline predisposition is associated with older age and usually involves single mutations affecting *DNMT3A*, *TET2*, or *ASXL1*^19,30^. Among 83 SDS patients without a MN diagnosis, 60 (72%) had detectable CH, 40 of whom had more than one mutation (median 3, range 1–21). Two of these patients had CH defined by clonal cytogenetic alterations in the absence of point mutations. Recurrent mutations in *EIF6* (49 of 83, 59.0%), *TP53* (33 of 83, 39.8%), *PRPF8* (9 of 83, 10.8%), and *CSNK1A1* (6 of 83, 7.2%) composed 96.9% of all somatic mutations, while typical CH mutations such as *DNMT3A*, *TET2*, or *ASXL1*, were rare (*n* = 1 for each) (Fig. 5a). CH mutations were present at low abundance irrespective of the affected gene, including *EIF6* (median VAF 0.0047, range 0.002–0.282), *TP53* (0.0044, range 0.002–0.193), *PRPF8* (0.0052, range 0.002–0.375), and *CSNK1A1* (0.0053, range 0.002–0.100) (Fig. 5b). CH was detectable in 27 of 46 patients (59%) 10 years old and younger, 24 of 27 patients (89%) 11–20 years old (89%) and 10 of 10 patients (100%) 21 years or older (Fig. 5c).

To assess the onset, persistence, and dynamics of CH mutations over time, we sequenced 208 serial samples from 49 SDS patients with CH (median 4 samples, range 2–11). We first analyzed serial samples from six patients who had developed CH prior to 10 years of age and for whom the initial sample was obtained at age 3 years or younger. In five cases, stable CH developed at older ages (3–10 years old), while in one case (SDS-034), a stable *EIF6* mutation was detected at time of first sampling at age 2 (Fig. 5d). Among six older patients whose last sample was obtained between ages 15 and 31 years, stable CH was detectable at the earliest available time point, 5–10 years prior (Fig. 5e).

Among all 49 patients with CH who had serial samples, most mutations were detected across multiple timepoints. Among persistent clones, we measured the mutation allele burden across serial timepoints and found that most clones remained stable at low VAF over time, with little change in relative abundance between initial detection and the most recent sample (Fig. 5f). None of the patients with CH involving *EIF6* or *TP53* had severe marrow failure despite the higher median age of the CH group compared to the group who developed severe marrow failure (Fig. 5g).

### Clonal evolution and development of leukemia

The diagnosis of MN was associated with the presence of somatic *TP53* mutations. However, *TP53* mutations were common in SDS clonal hematopoiesis and most were stable without hematologic progression across years of observation or detected only at a single timepoint. Therefore, we sought to identify additional genetic characteristics of *TP53* mutated leukemias that might enable distinction of CH clones with high-risk of transformation from those likely to remain clinically stable.

**Fig. 6:**
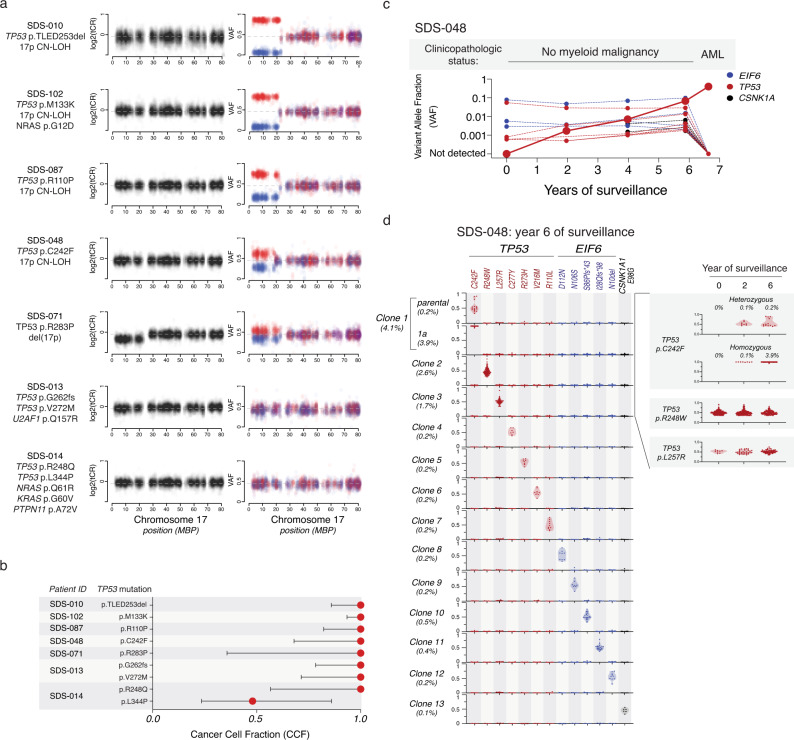
Biallelic *TP53* inactivation and myeloid neoplasia in patients with SDS. **a** Total copy ratio (tCR, denoted in black) and phased SNP-VAF (denoted in red/blue) across chromosome 17. **b** Cancer cell fraction of somatic *TP53* mutations in seven patient samples analyzed in panel **a**. **c** Shown are the clinicopathologic status and VAF of somatic *TP53* (red), *EIF6* (blue), and *CSNK1A* (black) mutations from bulk sequencing of serial samples from SDS-048, a patient with SDS who progressed to AML. **d** Single cell sequencing demonstrating clonal hierarchy from SDS-048 during serial surveillance prior to development of AML. Each row represents a unique clone or subclone and the frequency of each clone is indicated to the left. Columns reflect the genotype status of each mutation in each clone, and all depicted clones have complete genotyping at all loci. *Y*-axis indicates single cell VAF from 0 to 1, where 0 is absent, 0.5 is heterozygous mutation, and 1 is homo/hemizygous. Each dot reflects a single cell, colored according to gene mutation, *TP53* (red), *EIF6* (blue), *CSNK1A1* (black) and the frequency distribution of the data points reflected by shaded violin plots. Shown on the right is a time course indicating dynamics of the pre-leukemic p.C242F mutated clone and two independent *TP53*-mutated clones that did not transform.

We analyzed exomes from seven patients with *TP53*-mutated MN for allelic imbalances at the *TP53* locus by evaluating the total copy ratio (tCR) and SNP VAF across chromosome 17. We found that all seven patients had biallelic alteration of *TP53*, occurring by one of three mechanisms based on the number of *TP53* mutations (1 vs. 2 or more) and the presence of *TP53* deletion or copy-neutral loss of heterozygosity (CN-LOH). We observed 4 cases with monoallelic *TP53* mutations and 17p CN-LOH, 1 with monoallelic *TP53* mutation and 17p deletion, and 2 with biallelic *TP53* mutations (Fig. 6a). We next determined the fraction of clonal cells in which each *TP53* mutation was present, which defines its cancer cell fraction (CCF)^31,32^. In each case, the *TP53* mutations were present at high CCF, indicating that they were likely present in all cells of the leukemic clone (Fig. 6b). Among *TP53* mutated MN, 3 of 7 also harbored somatic mutations in typical myeloid drivers, including subclonal mutations in *NRAS* (*n* = 2), *KRAS*, or *PTPN11*. Somatic mutations in genes encoding effectors of RAS/MAPK signaling (*NRAS*, *KRAS*, *PTPN11*, *CBL*, *FLT3*, *RIT1*, *KIT*) were rarely present in samples from patients without morphologic transformation.

In 4 of 15 patients with morphologically defined MN, we did not detect somatic *TP53* mutations. All four of these patients had MDS. In two, we identified mutations in canonical myeloid driver genes, including one with *SETBP1*, *BRAF*, *NRAS*, and *KRAS* mutations and one with *ASXL1* and *ETNK1* mutations. Of the remaining two patients without point mutations, one had deletion of chromosome 7q and the other was diagnosed with MDS based on morphologic dysplasia without cytogenetic abnormalities or increased blasts.

### Biallelic *TP53* alterations identify leukemic subclones

Early detection of leukemia-associated genetic alterations could identify clones with increased leukemic potential prior to clinical transformation. We therefore sought to define the latency between detection of these mutations and clinical progression in a patient who developed AML despite having stable blood counts and no morphologic evidence of MN on bone marrow examinations during standard clinical surveillance. Using exome sequencing, we identified a *TP53* p.C242F mutation with CN-LOH in the AML sample (Fig. 6a), then used deep, error-corrected sequencing to quantify mutation allele burden in bulk DNA across serial samples obtained prior to transformation. The *TP53* p.C242F mutation was first detectable at low abundance (VAF = 0.17%) 4.5 years prior to transformation (Fig. 6c). In addition to this pre-leukemic mutation, we also identified 17 additional mutations from four samples across 6.6 years of surveillance (ages 16–22), including *TP53* (*n* = 7), *EIF6* (*n* = 7), *CSNK1A1* (*n* = 2), *PRPF8* (*n* = 1), and *SBDS* (*n* = 1). Throughout surveillance, the pre-leukemic *TP53* p.C242F clone was indistinguishable from other *TP53*-mutated clones based on low VAF and relative stability across serial samples.

Bulk sequencing cannot reliably identify interval acquisition of *TP53* allelic imbalance in small clones. We therefore sequenced 20,214 single cells across three samples obtained 6.5, 4.5, and 0.5 years before clinical transformation in order to identify the earliest evidence of *TP53* CN-LOH. All *TP53* mutations detected by bulk sequencing were also observed using single cell DNA sequencing, but only the *TP53* p.C242F clone displayed evidence of clonal evolution with *TP53* LOH. Concordant with bulk-sequencing data, the *TP53* p.C242F was first detectable 4.5 years prior to development of AML (Fig. 6d). The *TP53* p.C242F clone was initially present at low abundance (0.1%), with a balanced proportion of the heterozygous founding clone and the homozygous (CN-LOH) progression subclone. Subsequently, the CN-LOH subclone expanded selectively over the following 4 years prior to subsequent transformation. Other stable *TP53* mutations, including the most abundant p.R248W and p.L257R clones, defined independent clones and remained in the monoallelic state across 6.5 years of surveillance. These data indicate that development of *TP53* LOH events can precede frank transformation by several years and that single cell DNA sequencing enables detection of small clones defined by *TP53* LOH events.

## Discussion

**Fig. 7:**
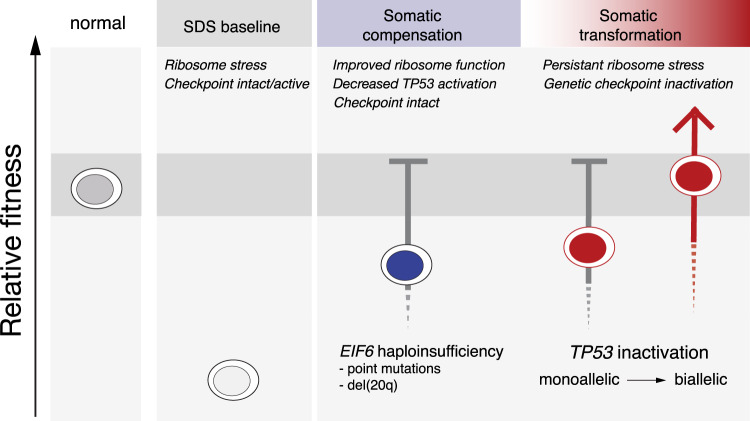
*TP53* and *EIF6* mutations define distinct pathways of somatic clonal progression and distinguish leukemia predisposition in SDS. Germline context drives separate compensatory and maladaptive somatic pathways of clonal evolution in patients with SDS. Germline *SBDS* mutations result in ribosomal stress which activate TP53 checkpoint pathways and promote bone marrow failure. *EIF6* mutations alleviate the underlying ribosome maturation defects which reduces p53 checkpoint activation and improves cell fitness. *TP53* mutations eliminate checkpoint pathways to improve relative fitness without improving the underlying ribosomal abnormalities, and promote the development of myeloid malignancies.

We found that germline SBDS deficiency establishes a global fitness constraint that drives selection of somatic clones via two pathways with distinct mechanisms and different clinical consequences. A compensatory pathway with limited leukemic potential, mediated predominantly by *EIF6* inactivation, enhances clone fitness by ameliorating the SDS ribosome defect. A maladaptive pathway with enhanced leukemic potential, driven by *TP53* inactivation, subverts normal tumor suppressor checkpoints without correcting the ribosome defect (Fig. 7).

Through analysis of serial samples collected in children and young adults with SDS, we demonstrate that somatic clones are infrequent in the first several years of life, but approach ubiquity in the second decade. Many patients had multiple somatic mutations, but this high somatic mutation burden typically reflects a composite of multiple, genetically distinct and independently arising clones rather than a single clone with complex subclonal evolution. Most of these somatic clones in SDS patients carried a mutation in one of only four genes (*EIF6*, *TP53*, *PRPF8*, and *CSNK1A1*), and rarely involved genes commonly mutated in age-related CH (*DNMT3A*, *TET2*, *ASXL1*). These results provide genetic evidence that germline SBDS deficiency causes a global, disease-specific HSC fitness constraint that drives parallel development of somatic CH at an early age.

We show that somatic *EIF6* mutations are common in SDS and that they cause functional compensation of SBDS deficiency by rescuing the SDS ribosome joining defect, improving translation, and reducing p53 activation. Using structural modeling and functional studies, we demonstrate that *EIF6* missense mutations exert these effects by either disrupting the binding interaction between EIF6 and the 60S subunit or by destabilizing the EIF6 protein. Our findings in SDS patients are consistent with prior studies in yeast, where mutations in the *EIF6* homolog *TIF6* were shown to attenuate the slow growth phenotype seen with deletion of the *SBDS* homolog *SDO1*^27^. Notably, several residues that we found to be recurrently mutated in SDS patients, including p.G14, p.G105, and p.N106, are invariant between human and Archaea and were found in a yeast genetic screen to cause reduced affinity for 60S subunits. Our study contrasts with a mouse model of *Eif6* haploinsufficiency in non-SDS cells, where reduced Eif6 levels impaired translational efficiency, reduced proliferation, and delayed Myc-dependent lymphomagenesis^33^.

Normalization of germline-encoded hematopoietic defects through somatic reversion has been observed in inherited bone marrow failure syndromes^34–36^. In SDS, we found that somatic mutations mitigate the cellular consequences of SBDS deficiency via compensatory mechanisms without direct reversion or correction of the causative genetic lesion. Specifically, highly recurrent functional inactivation of EIF6 via multiple genetic mechanisms (point mutations and interstitial del(20q)) indicates that normalizing the functional ratio of SBDS:EIF6 protein in SDS hematopoietic cells enhances competitive fitness by improving ribosome maturation and translational capacity. *EIF6* alterations are not associated with leukemic transformation or *TP53* co-mutation within the same cell and were not found in patients with severe bone marrow failure, suggesting that functional correction of germline-encoded cellular defects may drive enhanced fitness of somatic clones without altering normal pathways of differentiation or tumor suppression. Our results support EIF6 as a potential therapeutic target in SDS patients, since pharmacologic inactivation of EIF6 could mimic genetic inactivation of *EIF6*, thereby reducing leukemia risk and improving hematopoietic function in SDS patients.

The presence, number, persistence, and allele abundance of somatic *TP53* mutations were not predictive of imminent leukemia risk in SDS patients with CH. Biological and clinical heterogeneity among different hotspot *TP53* mutations has been reported^37^, but we were unable to evaluate this question due to sample size limitation. Our results indicate that progression of *TP53*-mutated clones is driven by development of biallelic alterations of the *TP53* locus via deletion, CN-LOH, or point mutation, consistent with recent findings in patients with sporadic MDS^37^. Importantly, we found that SDS patients can develop multiple, independent *TP53*-mutated clones and that serial monitoring by bulk sequencing fails to distinguish clinically significant subclonal changes in *TP53* allelic state. These findings suggest that integration of single cell DNA sequencing into surveillance strategies might identify patients with high risk clones prior to clinical transformation. In conclusion, our study elucidates biological mechanisms driving distinct pathways of CH in SDS and defines a framework for rational surveillance. An improved ability to identify patients with high risk of developing leukemia has the potential to improve clinical outcomes by enabling preemptive intervention with curative therapies, such as allogeneic transplantation.

## Methods

### Cell culture

Human leukemia cell lines (K562, isogenic K562 with a CRISPR-HDR corrected *TP53* allele^38^), kindly provided by Benjamin Ebert (Dana Farber Cancer Institute) were maintained in RPMI media (Gibco, 11875-119) supplemented with 10% FBS, penicillin and streptomycin (Gibco). Primary cultures of bone marrow fibroblasts were established and maintained in Chang D media (Irvine Scientific, T105). Mobilized peripheral blood CD34+ and bone marrow mononuclear cells were maintained in GMP SCGM Serum-free Media (Cellgenix, 20802–0500) supplemented with 100 ng/mL hSCF (Peprotech, 300-07), hTPO (Peprotech, 300-18), hFLT3-L (Peprotech, 300-19), and for bone marrow mononuclear cell culture, 20 ng/mL of IL-3 (R&D systems 203-IL-010/CF) was added. All cells lines were cultured at 37 °C under 5% CO_2_ and routinely screened for mycoplasma^39^.

### Colony formation assays

For methylcellulose colony formation assays, G-CSF mobilized peripheral blood CD34+ cells (Fred Hutch CCEH Core B) were resuspended in GMP SCGM Serum-free Media plus cytokines noted above (Cellgenix, 20802-0500) and allowed to recover for 36 h. Cells were transduced with indicated lentiviral vectors and for CD34+ cells were sorted using BD FACSAria (BD biosciences) to obtain double-positive population. Doublets were excluded using standard methods of FSC/FSC-A doublet exclusion and then distinctly positive RFP and GFP cells were selected based on comparison to untransduced CD34+ cells(gating shown in Supplementary Fig. 5A). Then 1750 CD34+ cells were added to 3.5 ml of methylcellulose (Stem Cell Technologies, H4434). One milliliter was plated in triplicate wells of six-well Smartdishes (Stem Cell Technologies, 27370). After 12 days of growth at 37 °C/5% CO_2_, colonies were imaged, results blinded and then counted using STEMVision (Stem Cell Technologies). Counts were averaged for triplicate wells.

### Plasmids, cloning and site-directed mutagenesis

Gateway vectors containing EIF6 cDNA were obtained from the Harvard Plasmid Repository in closed format (clone ID HsCD00044644) or open format (clone ID HsCD00041550). Site-directed mutagenesis was performed using the NEB Q5 site-directed mutagenesis kit according to manufacturer instructions (New England Biolabs, E0554S) using primers listed in Supplementary Table 4. Gateway cloning was performed using LR clonase (Invitrogen, 11791-020) according to manufacturer instructions. Closed constructs were cloned into pRRL-SFFV-gwdest. Open constructs were cloned into constructs with V5 C-terminal tags: tetracycline inducible pLIX-403 or constitutive expression pLX304. pLIX-403 and pLX304^40^ were gifts from David Root (pLIX-403 is Addgene plasmid #41395, pLX304 is Addgene plasmid #25890). Tetracycline-inducible short hairpin RNAs targeting SBDS were made by annealing oligos and ligating with T4 ligase (New England Biolabs, M0202) into AgeI and EcoRI-digested Tet-pLKO-puro. Tet-pLKO-puro was a gift from Dmitri Wiederschain (Addgene plasmid #21915)^41^.

### Immunofluorescence

Primary bone marrow fibroblasts from SDS patients were grown on coverslips in a six-well plate at a density of 3 × 10^5^ cells/coverslip for 24 hs. Cells were washed with PBS (Gibco), fixed with 4% (w/v) paraformaldehyde (MilliporeSigma) for 10 min at room temperature, washed three times with PBS, permeabilized with 0.2% Triton X-100 (VWR) for 5 min at room temperature, washed three times with PBS, and blocked for 30 min at room temperature in solution with 3% BSA (MilliporeSigma) before 30 min incubation with primary antibody at room temperature (Fibrillarin, Cell Signaling, 2639, clone C13C3, Lot: 2, 1:1000), (V5, Medical and Bio Labs, M215-3, clone OZA3, Lot: 003, 1:10,000). Coverslips were washed three times with 1% Triton (VWR) in PBS before incubation with secondary antibody (AlexaFluor 594 donkey anti-rabbit IgG (Fisher Scientific, A21207, Lot: 1827674, 1:1000) or AlexaFluor 488 donkey anti-mouse (Life Technologies, A-21202, Lot: 1820538, 1:1000)). Cells were mounted with mounting medium containing DAPI (Vector Laboratories, H-1200) for nuclear counterstaining and were imaged with a Nikon Eclipse 90i microscope.

### Polysome profiling

Ribosomal subunits were separated by sucrose density gradients as described^28^. Briefly, 4 × 10^6^ fibroblasts (80% confluence) or 2.5 × 10^6^ K562 (1 million/mL) were treated with cycloheximide at final concentration of 100 µg/mL for 10 min at 37 °C before harvesting. Cells were then lysed in lysis buffer (20 mM HEPES pH 7.4, 100 mM KCl, 10 mM MgCl_2_, 1% [w/v] NP-40, 1% [w/v] deoxycholate, 100 µg/mL cycloheximide, 1 mM DTT, 200 µg/mL heparin, with complete EDTA-free protease inhibitors (Roche), 1.4 µM pepstatin A, and 40 U/mL Rnasin (Promega N2115) and incubated for 10 min on ice. Lysates were cleared in a microcentrifuge at 4 °C. Equal amounts were applied to a 5–50% (w/v) sucrose gradient in gradient buffer (10 mM HEPES pH 7.4, 50 mM KCl, 5 mM MgCl_2_, 100 µg/mL cycloheximide) and centrifuged (Beckman SW55.1 rotor at 246,000 × *g* for 1 h and 15 min at 4 °C). The sucrose gradient was made using a Biocomp Gradient Master. Centrifugation samples were unloaded using a Brandel gradient fractionator, polysome profiles detected at 254 nM absorbance and area under the curve for 80S and 60S peaks quantitated using Peakchart version 2.08 (Brandel). 0.1 mL fractions collected into Laemmli sample buffer and separated on SDS–PAGE gels and transferred to PVDF membranes for immunoblotting.

### qRT-PCR analysis of transcription

RNA was isolated following manufacturer’s instructions for RNeasy Plus Mini Kit (QIAGEN Inc., 44134). RNA was eluted in 30 μl of water. We used 200 ng–1 μg of RNA for reverse transcription with Superscript III First Strand Synthesis using oligo-dT primer (Invitrogen, 18080051). For qPCR analysis, cDNAs were diluted threefold in MilliQ water. Quantitative PCR was performed with iTaq Universal SYBR Green Supermix (Bio-Rad, 1725125). The linear range of amplification for each primer pair was confirmed by serial dilution of genomic DNA from K562 cells. Reactions were carried out in triplicate in a 7500 Fast real-time PCR System (Applied Biosystems) and analyzed using the ΔΔCT method^42^. The primer sequences are shown in Supplementary Table 4.

### Virus production and titration

Transfection of 293T cells (obtained from Dr. David Williams’ laboratory) was performed as described^43^. Lentiviral vector supernatants were generated by cotransfecting lentiviral transfer vectors (pRRL-SFFV-gwdest, pLIX-403 (Addgene plasmid #41395), pLX304 (Addgene plasmid #25890), tet-pLKO-puro (Addgene plasmid #21915), SMARTvector-human-shEIF6 (Dharmacon V3SH11243-07EG3692), SMARTvector-hCMV-shTP53-TurboGFP (Dharmacon V3SH11243-00EG7157), SMARTvector-hCMV-shTP53-TurboRFP (Dharmacon V3SH11243-07EG7157), pL40C.SFFV.eGFP.miR30N.PRE-shLUC^44^ (CGCTGAGTACTTCGAAATGTC) or pL40C.SFFV.eGFP.miR30N.PRE-shSBDS with packaging plasmids psPAX2 (Addgene plasmid #12260) and pMD2.G (Addgene plasmid #12259) using PEI reagent (Polysciences #23966-2). Supernatants were collected, filtered through a 0.45 µm membrane (ThermoFisher, 165-0045), and subsequently concentrated by ultracentrifugation at 10,000 rpm for 10 h in a Beckmann XL-90 centrifuge using SW-28 swinging buckets. To determine the titer, HT1080 cells (obtained from Dr. David Williams’ Laboratory) were infected with the virus in the presence of 8 µg/ml polybrene (Santa Cruz, SC134220) and analyzed 48 h post-transduction by fluorescence-activated cell sorting for GFP expression.

### Growth competition assay

K562 *TP53-*corrected cells containing puromycin selectable doxycycline inducible shRNA-targeting SBDS (target sequence GCTTGGATGATGTTCCTGATT) were transduced with lentivirus encoding constitutively expressed EIF6-RFP mutant or wild-type constructs as noted. After 48 h, cells were admixed at a ratio of 1:1 and subjected to flow cytometry (gating shown in Supplementary Fig. 5B) at indicated times on a Fortessa HTS flow cytometer (BD Biosciences). Doxycycline (Clonetech 1 µg/mL) was refreshed daily and cells were maintained in puromycin (Mirus, 2 µg/mL) throughout the experiment. Analysis was performed at indicated timepoints with FacsDIVA software (BD Biosciences). Doublets were excluded using standard methods of FSC-H/FSC-A doublet exclusion and then the number of distinctly positive RFP cells analyzed based on comparison to untransduced cells.

### Immunoblotting

Cells were lysed in RIPA buffer (MilliporeSigma) supplemented with protease inhibitors (Inhibitab, Roche). Protein concentrations were determined by colorimetric assay (BCA Protein, Thermo Fisher Scientific), and 20–40 μg of protein was loaded on 12% SDS–PAGE gels and blotted on a PVDF membrane (MilliporeSigma, IPVH00010). The membranes were blocked with 5% nonfat dry milk (VWR) diluted in Tris-buffered saline (Teknova, T1680) with 1% Tween-20 (VWR, M147-1L). Primary antibodies SBDS^45^ (homemade, B1872, 1:10,000), GAPDH (Cell Signaling, 2118, clone 14C10, Lot: 10, 1:1000), eIF6 (Cell Signaling, 3833, clone D16E9, Lot: 1, 1:1000), p53 (Calbiochem, OP43, clone DO-1, Lot: 3182624, 1:1000), RPL3 (Abcam, ab241412, polyclonal, Lot: GR3251648-4, 1:1000), Ubiquitin (Cell Signaling, 3933, polyclonal, Lot: 6, 1:1000), V5 tag (Abcam, ab15828, polyclonal, Lot: GR3265659-1, 1:1000), Vinculin (Invitrogen, 700062, clone 42H89L44, Lot: 2090723, 1:5000), were incubated overnight at 4 °C. After washing with TBS-T, membranes were incubated with HRP-conjugated secondary antibodies ECL anti-rabbit IgG (GE Healthcare, NA934V, Lot: 16897770, 1:10,000; Cell Signaling, 7074, Lot: 26, 1:10,000) and ECL anti-mouse (GE Healthcare, NA931V, Lot: 16895895, 1:10,000) and developed using SuperSignal West Pico Chemiluminescent substrate (Thermo Fisher Scientific, 34094). Detection of bands was conducted in the Amersham Imager 600 (GE Healthcare).

### OP-Puro incorporation

OP-Puro (Medchem Source; Life Technologies, C10459; 50 μM final concentration) was added to the culture medium for 3 h and incubated in a 37 °C incubator. Cells were removed from wells and washed twice in Ca^2+^ and Mg^2+^ free phosphate buffered saline (PBS) + cycloheximide. Cells were fixed in 0.5 ml of 1% paraformaldehyde in PBS for 10 min, then permeabilized in PBS supplemented with 3% fetal bovine serum and 0.1% saponin for 5 min at room temperature. The azide-alkyne cycloaddition was performed using the Click-iT Cell Reaction Buffer Kit (Life Technologies, C10458) and azide conjugated to Alexa Fluor 647 (Life Technologies, C10458) at 5 μM final concentration for 30 min. The cells were washed twice in PBS and passed through a filter top tube prior to being analyzed by flow cytometry using a Fortessa HTS flow cytometer (BD Biosciences). Doublets were excluded using standard methods of FSC-H/FSC-A doublet exclusion and then distinctly positive RFP or GFP cells were selected based on comparison to control cells (gating shown in Supplementary Fig. 5C). Mean fluorescence intensity of positive cells was quantified using FlowJo (FlowJo LLC) version 10.3.0.

### Statistical analysis

Graphpad Prism version 8 and SAS 9.4 (SAS Institute, Cary, NC) was used to analyze results and create graphs. Fisher’s exact test is used to assess the association between presence of mutations and patient characteristics. Wilcoxon rank sum test is used to assess the association between number of mutations and patient characteristics. Results are considered significantly associated with outcome if *p*-values < 0.05 and marginally associated with outcome if *p* < 0.10.

### Patients and samples

Subjects provided written informed consent for protocols approved by the institutional review boards of Boston Children’s Hospital and Cincinnati Children’s Hospital, in accordance with the Declaration of Helsinki’s Ethical Principles of Medical Research Involving Human Subjects. All subjects or their guardian provided written informed consent prior to their participation in the study. This informed consent included permission to publish. Clinical criteria for SDS diagnosis were as described in consensus guidelines^46^.

### DNA extraction

DNA was extracted from patient samples and patient fibroblasts using the QIAamp DNA Blood Mini kit (Qiagen, 51104) according to manufacturer instructions.

### Single-cell DNA sequencing

We designed a custom panel covering seven genes implicated in SDS or sporadic clonal hematopoiesis and 43 single nucleotide polymorphism loci on chromosomes 7, 17, and 20 for ADO determination (Supplementary Table 5). Libraries were generated from cyropreserved or fresh bone marrow mononuclear cells with the Mission Bio Tapestri Single-cell DNA custom Kit according to manufacturer’s instruction (Mission Bio) with the following modifications: concentration of cell input was increased by 15% to 3500–4500 cells per microliter and library PCR cycles were increased by one cycle. Libraries were pooled in equimolar concentration and sequenced on a NovaSeq (Illumina) on a 150 base pair paired end run. FASTQ files were processed using the Tapestri Pipeline for adapter trimming, alignment, barcode correction, cell finding, and variant calling. Loom files that were generated by the Tapestri Pipeline using GATK-based haplotype calling and subjected to the following genotyping criteria: total read count (depth, DP) ≥ 10, alternative allele count ≥ 3, alternate single cell allele fraction ≥ 30%. Only variants defined based on duplex UMI bulk sequencing data were included in this analysis; no de novo variant calling was performed using the scDNA sequencing data (Tapestri Insights 2.2, Mission Bio). To quantify the technical dropout, we defined the proportion of targeted mutations, which were defined as those (1) present in the bulk sequencing and (2) located in designable amplicons, for which genotyping was successful. To quantify ADO, we evaluated informative SNPs within exonic targets and a series of informative intergenic SNPs included in our design. For each variant, we determined the ADO by dividing the number of cells with homozygous reference or variant genotype by the number of cells with heterozygous genotype. The mean sample-level ADO was 10.1% (95% CI 8.3–11.8%) and mean site-specific ADO rate was 10.4% (95% CI 9.8–11.9%).

### Whole exome sequencing

Prior to library preparation, DNA was fragmented (Covaris sonication) to 250 bp and further purified using Agentcourt AMPure XP beads. Size-selected DNA was then ligated to specific adapters during automated library preparation (SPRIworks, Beckman-Coulter). Libraries were pooled and sequenced on an Illumina MiSeq to estimate the concentration based on the number of barcode reads per sample). Library construction is considered successful if the yield is ≥250 ng. Libraries were pooled in equal mass to a total of 750 ng for SureSelect Human All Exon V5 enrichment using the Agilent SureSelect hybrid capture kit. Captures were further pooled and sequenced on HiSeq2500 or HiSeq3000 (Illumina). Pooled sample reads were de-convoluted (de-multiplexed) and sorted using the Picard tools^47^. Reads were aligned to the reference sequence b37 edition from the Human Genome Reference Consortium using “bwa aln” (http://bio-bwa.sourceforge.net/bwa.shtml) using the following parameters “-q 5 -l 32 -k 2 -o 1” and duplicate reads were identified and removed using the Picard tools. The alignments were further refined using the GATK tool for localized realignment around indel sites. Recalibration of the quality scores was also performed using GATK tools^48,49^. Metrics for the representation of each sample were generated on the unaligned reads after sorting on the barcode. Fingerprinting analysis was performed using 44 polymorphic loci to identify if the aggregation pairing strategy was performed appropriately. Picard Tools GenotypeConcordance was used to calculate the concordance that a given test sample matches the sample being considered. This was performed on all pairwise combinations of samples in the cohort. The output of the pairwise comparisons was then mapped to a concordance matrix, where concordance values above 4 standard deviations of the median concordance value for the cohort indicated a high likelihood that the samples match. Samples can match for reasons other than being from the same individual, so potential matches are manually reviewed where applicable.

For bone marrow samples, median of mean target coverage = 136×, range 101–198×. Mutation analysis for single nucleotide variants (SNV) was performed using MuTect v1.1.4 and annotated by Variant Effect Predictor (VEP). We used the SomaticIndelDetector tool that is part of the GATK for indel calling. VEP v79 is used for annotating the variants. MuTect was run in paired mode with bone marrow aspirate and cultured fibroblast samples from each subject. Variants that affected protein coding regions underwent further filtering/classification based on frequency in the gnomAD, ESP, and COSMIC (version 80) databases. Variants that affect protein coding regions were flagged as “REVIEW_REQUIRED”, if the frequency of the variant is ≤1% in all gnomAD and ESP populations or if the frequency of the variant is >1% and ≤10% in all gnomAD and ESP populations and present in “COSMIC” database at least two times. Variants were flagged as “NO_REVIEW_GERMLINE_FILTER” if the frequency of the variant is between 1% and ≤10% in all gnomAD and ESP populations and not present in “COSMIC” database at least two times or if the frequency of the variant is >10% in any gnomAD and ESP populations. Variants with frequency >10% in any gnomAD or ESP population were considered to be a common SNP irrespective of presence in the COSMIC database.

### Copy number analysis

To obtain raw copy-number estimates across the genome of each sample, the number of unique templates mapping to each exome target region (padded by 250 bp) was extracted from the BAM file. The raw estimates were normalized against coverage obtained from a panel of diploid normal samples. A subset of targets was removed based on estimates of mean total copy-ratio and the standard deviation of copy-ratio estimates within a panel of diploid normal samples. The resulting total copy-ratio profiles were then segmented using an adaptation of the circular binary segmentation algorithm, which includes information from all patient samples when segmenting. Subsequently, the allele-specific copy number was estimated by examining the template counts supporting alternative and reference alleles at germline heterozygous SNP sites within the 1000 Genomes Phase 3 variants. Of the 1000 Genomes Phase 3 variants, a patient was considered heterozygous at a given locus based on the number of reference and alternative template counts observed in all patient samples. The allele-specific template counts were then used to infer allele-specific copy ratios as described previously serving as input into ABSOLUTE v.1.4, which jointly estimated the fraction of cancer cells, cancer ploidy and absolute allelic copy numbers across the genome^31^. For each somatic *TP53* mutation, we estimated the fraction of cancer cells that harbors the mutation [its CCF], represented as a distribution over the possible CCF values, between 0 and 1. A CCF value of 1 indicates that mutations are present in 100% of clonal cells in the sample. A CCF value of <1 indicates that the mutation is subclonal, and present in only a subset of the clonal cells in the sample. Probability distributions over CCF were computed for each *TP53* mutation by correcting mutant and reference read fractions for sample purity and local copy-number^31,32^.

### Phasing

In each patient, a genotype was estimated at known polymorphic sites in the 1000 Genomes project. Specifically, a subset of the polymorphic 1000 genomes sites that intersected the whole exome targets. At each site, the evidence for a genotype (AA, AB, BB) was calculated based on reference and alternate allele counts and was additionally informed by population allele frequencies, which served as priors for genotyping. Given the cohort of patients that were genotyped at the polymorphic locations, haplotypes (AB vs. BA) were estimated using SHAPEIT2 and a haplotype panel from the 1000 Genome consortium. A hidden markov model was used to integrate these estimates of haplotype along with reference and alternate allele counts to create a maximum a posteriori estimate of haplotype at each heterozygous SNP in each patient^50^.

### Targeted deep sequencing

We selected 55 genes for targeted sequencing based on their recurrent alteration in SDS exome cohort and myeloid malignancies^13^ (Supplementary Table 2). We included 48 single nucleotide polymorphisms (SNPs) for establishing subject concordance of serial samples.

### Library construction

An aliquot of genomic DNA (250 ng in 50 µL) was used as the input into DNA fragmentation. Shearing was performed acoustically using a Covaris focused-ultrasonicator, targeting 150 bp fragments. Library preparation was performed using a commercially available kit provided by KAPA Biosystems (KAPA HyperPrep Kit with Library Amplification product KK8504) and IDT’s duplex UMI adapters. The libraries were then paired with unique 8-base dual index sequences embedded within the p5 and p7 primers (purchased from IDT) added during PCR. Enzymatic clean-ups were performed using Beckman Coulter AMPure XP beads with elution volumes reduced to 30 µL to maximize library concentration. In addition, during the post-enrichment SPRI cleanup, elution volume was reduced to 30 µL to maximize library concentration, and a vortexing step was added to maximize the amount of template eluted.

### Post library construction quantification and normalization

Library quantification was performed using the Invitrogen Quant-It broad range dsDNA quantification assay kit (Thermo Scientific Catalog: Q33130) with a 1:200 PicoGreen dilution. Following quantification, each library is normalized to a concentration of 35 ng/µL, using Tris–HCl, 10 mM, pH 8.0.

### In-solution hybrid selection

After library construction, hybridization and capture are performed using the relevant components of IDT’s XGen hybridization and wash kit and following the manufacturer’s suggested protocol, with several exceptions. A set of 12-plex pre-hybridization pools are created. These pre-hybridization pools are created by equivolume pooling of the normalized libraries, Human Cot-1 and IDT XGen blocking oligos. The pre-hybridization pools undergo lyophilization using the Biotage SPE-DRY. Post lyophilization, custom exome bait (TWIST Biosciences) along with hybridization mastermix is added to the lyophilized pool prior to resuspension. Samples are incubated overnight. Library normalization and hybridization setup are performed on a Hamilton Starlet liquid handling platform, while target capture is performed on the Agilent Bravo automated platform. Post capture, a PCR is performed to amplify the capture material.

After post-capture enrichment, library pools are quantified using qPCR (automated assay on the Agilent Bravo), using a kit purchased from KAPA Biosystems with probes specific to the ends of the adapters. Based on qPCR quantification, pools are normalized using a Hamilton Starlet to 2 nM and sequenced using Illumina sequencing technology.

### Cluster amplification and sequencing

Cluster amplification of library pools was performed according to the manufacturer’s protocol (Illumina) using Exclusion Amplification cluster chemistry and HiSeq X flowcells. Flowcells were sequenced on v2 Sequencing-by-Synthesis chemistry for HiSeq X flowcells. The flowcells are then analyzed using RTA v.2.7.3 or later. Each pool of whole genome libraries was run on paired 151 bp runs, reading the dual-indexed sequences to identify molecular indices and sequenced across the number of lanes needed to meet coverage for all libraries in the pool.

### Variant calling pipeline

Reads are aligned with bwa-mem 0.7.15. Duplex consensus reads are called with fgbio 1.0 and realigned using bwa-mem. Consensus reads are required to have reads from both families αβ and βα, and consensus reads with Ns in excess of 5% of bases are discarded. Read one and two are soft-clipped from the 5′ end by 10 bases to reduce errors due to end repair. Single nucleotide and small insertion and deletion calling was performed with samtools-0.1.18 mpileup and Varscan 2.2.3. Variants were annotated to include information about cDNA and amino acid changes, sequence depth, number, and percentage of reads supporting the variant allele, population allele frequency in 1000 Genomes release 2.2.2^51^, the Genome Aggregation Database (gnomAD)^52^, and presence in Catalog of Somatic Mutations in Cancer (COSMIC), version 64.6^53^. Variants were excluded if they had fewer than three total duplex-reassembled alternate reads at the position or had variant allele fraction <0.1%, fell outside of the target coordinates, had excessive read strand bias, had excessive number of calls in the local region, caused synonymous changes, or were recurrent small insertions/deletions at low variant allele fraction adjacent to homopolymer repeat regions. Somatic status was determined using cultured fibroblast DNA as a germline reference tissue comparator. Individual single nucleotide substitutions and small insertions or deletions were evaluated as candidate drivers of MDS or bone marrow failure based on gene-specific characteristics, then curated manually and classified as MDS driver mutations or pathogenic bone marrow failure mutations based on genetic criteria and literature review^13,54,55^. Variant level details are available in Supplementary Table 6. All interpretation of variants was blinded to clinical characteristics and thus agnostic to variables including age, sex, diagnosis, treatment status, and clinical outcomes; the genetic analysis was completed and locked prior to merging with any clinical data.

### EIF6 model and mutational analysis

A human EIF6 structural model was generated using Rosetta^56,57^, and the structure was then further refined in Rosetta using the FastRelax algorithm^58^ with the Rosetta-ICO energy function^59^. Individual point mutations were evaluated for the predicted change in protein stability (ΔΔ*G*_mutation_) by introducing point mutations into the EIF6 structural model and calculating the change in energy (i.e. Rosetta total_score) relative to the native structure. The residues at the interface between EIF6 and RPL23 are 100% conserved between our model of human EIF6 and *D. discoideum* EIF6; thus, PDB ID 5ANB^22^ was refined in the same way and used for the ΔΔ*G*_bind_ calculations, which were performed using the Flex ddG method^23^. All scripts that were used for refinement and analysis are provided as Supplementary Software 1. Conservation scores were calculated using the ConSurf server^60^, and structural images were generated using PyMOL Molecular Graphics System version 1.8.4.0.

### Reporting summary

Further information on research design is available in the Nature Research Reporting Summary linked to this article.

## Supplementary information

Supplementary Information

Descriptions of Additional Supplementary Files

Supplementary Software 1

Reporting Summary

## Data Availability

The whole exome, single cell, and targeted sequencing data are deposited in the European Genome-phenome Archive (EGA), database under accession codes EGAS00001004879, EGAS00001004880, EGAS00001004881 and can be found on https://ega-archive.org. The data is available under restricted access, access can be obtained by contacting Akiko Shimamura and R. Coleman Lindsley. Source data are available as a Source Data file. The remaining data are available within the Article, Supplementary Information or available from the authors upon request. Source data are provided with this paper.

## Source data

Source data

## Code availability

Scripts used for Rosetta are included with the paper as Supplementary Software 1.
