# Supplementary material for: Distinct genetic pathways define pre-malignant versus compensatory clonal hematopoiesis in Shwachman-Diamond syndrome
Source: Nat Commun. 2021 Feb 26;12:1334. doi: 10.1038/s41467-021-21588-4 (PMC7910481; doi:10.1038/s41467-021-21588-4)
Supplement: Supplementary file 1 — Supplementary Information [file 41467_2021_21588_MOESM1_ESM.pdf]

Supplementary Information

Supplementary Figure 1

a

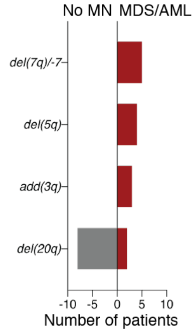

Supplementary Figure 1: Recurrent structural alterations found in patient cohort with or without myeloid neoplasm. Shown are the indicated somatic copy number alterations in the exome cohort without (grey bar) or with (red bars) myeloid neoplasm.

Supplementary Figure 2

a

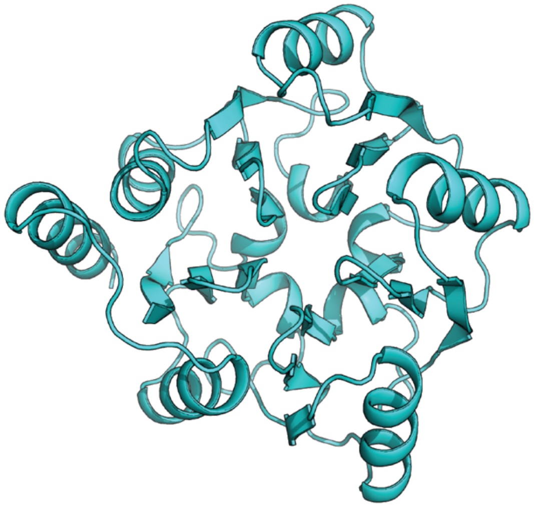

Supplementary Figure 2: Homology model of human EIF6 protein. Propeller like structural model of human EIF6 with five conserved ββαβ motifs.

Supplementary Figure 3

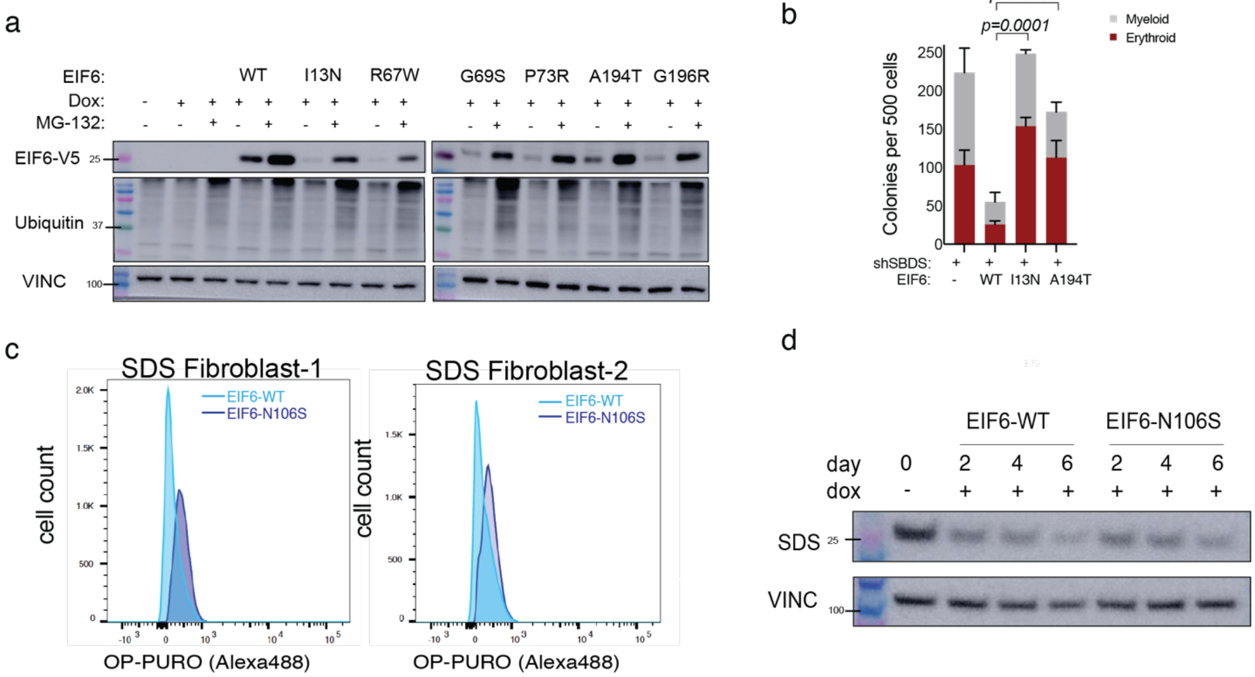

Supplementary Figure 3: Mutant EIF6 protein degradation is proteasome dependent and loss of function mutations lead to increased colony formation and translation. **A,** Immunoblot of K562 cells expressing doxycycline-inducible V5-tagged wild type EIF6 (WT) or mutant EIF6 cDNAs and treated with MG-132 proteasome inhibitor as indicated. The blots were probed with antibodies against the V5 tag, ubiquitin, or GAPDH. Ubiquitin serves as control for MG-132 activity and GAPDH as loading control. Data shown is representative of 3 independent experiments. **B**, Quantification of colony forming units from sorted CD34+ transduced with shSBDS-GFP together with the indicated EIF6 expression vectors (EIF6-WT-RFP or EIF6-I13N-RFP or EIF6-A194T-RFP) plated in triplicate. Shown is representative of 3 independent experiments. **C,**OP-Puro incorporation in two different primary SDS patient-derived fibroblast cell lines transduced with cDNAs of EIF6-WT or EIF6-N106S. Error bars represent the mean +/- standard deviation. **D**, Western blot of K562 cells expressing inducible shRNAs targeting SBDS at timepoints indicated. Data shown is representative of 3 independent experiments.

Supplementary Figure 4

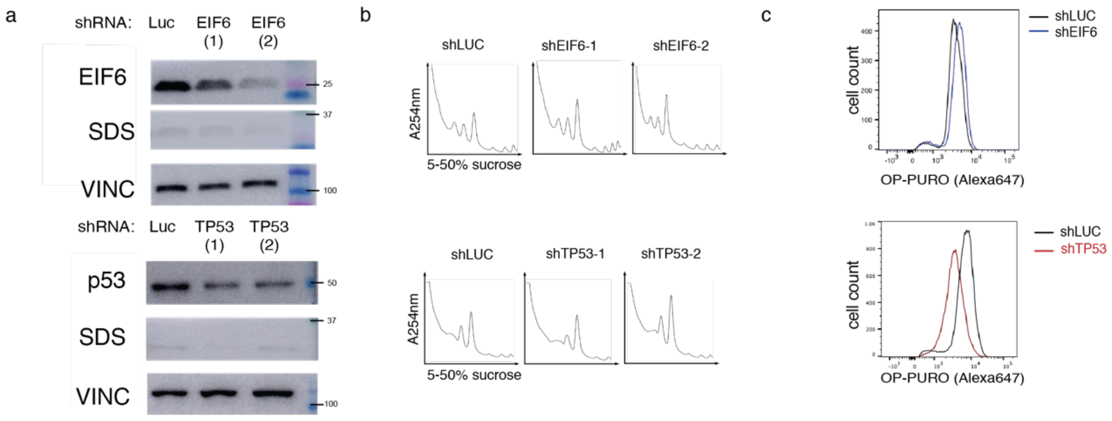

Supplementary Figure 4: Knockdown or mutations in *EIF6* lead to improved ribosome maturation, translation and colony formation. **A,** EIF6, SBDS and vinculin immunoblots of primary SDS patient-derived fibroblasts transduced with shRNAs targeting luciferase control, EIF6 (top panel) or TP53 (bottom panel). Data shown is representative of 3 independent experiments**. B,** Polysome profiles of cells in panel A. **C,** Biological replicate of data shown in Figure 3B. OP-Puro incorporation in primary SDS patient-derived fibroblasts transduced with shRNAs targeting luciferase, EIF6 (upper panel) or TP53 (lower panel).

Supplementary Figure 5

a. CD34+ cell sorting gating strategy

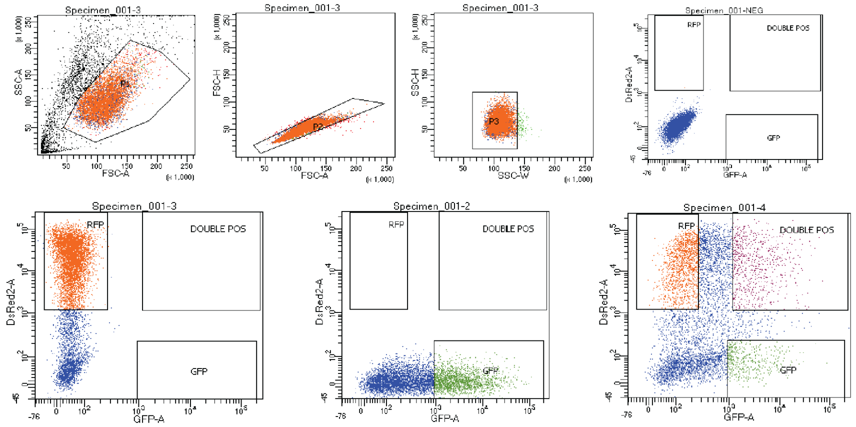

b. Growth Competition Gating Strategy

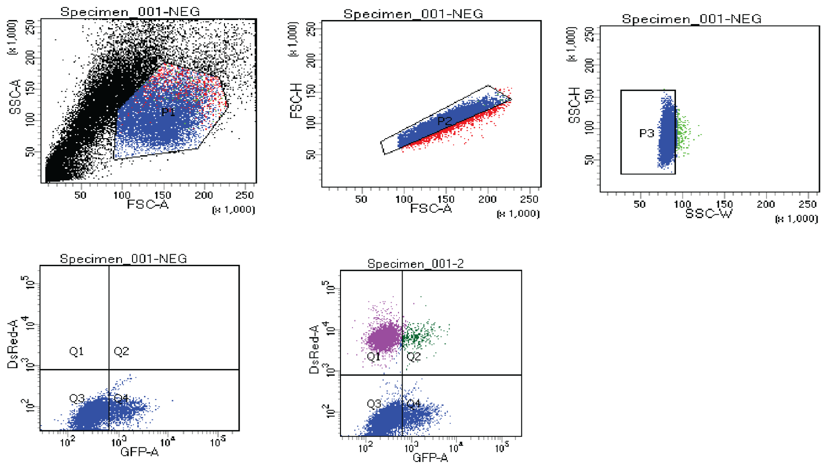

c. OP-PURO Gating Strategy

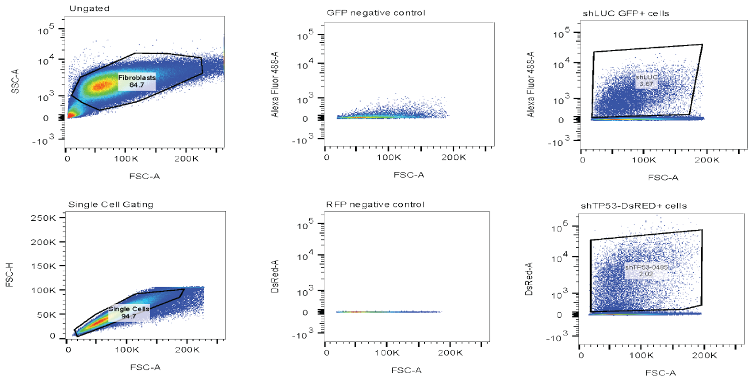

Supplementary Figure 5: Summary of flow cytometry gating strategies. A, Gating strategy for CD34 cells. **B,** Gating strategy for growth competition experiments. **C.** Gating strategy for OP-PURO.

Supplementary Table 1: Whole Exome/Discovery Sequencing Cohort:

SDS-10	SDS-48	SDS-78
SDS-11	SDS-49	SDS-84
SDS-13	SDS-57	SDS-86
SDS-14	SDS-62	SDS-87
SDS-18	SDS-63	SDS-89
SDS-21	SDS-65	SDS-92
SDS-36	SDS-71	SDS-95
SDS-39	SDS-72	SDS-98
SDS-40	SDS-75	SDS-99
SDS-47	SDS-77	SDS-102

Supplementary table 2: Targeted sequencing gene list and coordinates.

Chromosome	position start	position stop	Gene ID
chr1	1737908	1737983	gnb1_e6
chr1	1747189	1747308	gnb1_e5
chr1	36931638	36931807	csf3r_e18
chr1	36931952	36932515	csf3r_e17
chr1	36932825	36932918	csf3r_e16
chr1	36933153	36933258	csf3r_e15
chr1	36933417	36933569	csf3r_e14
chr1	43814928	43815036	MPL_e10
chr1	115251145	115251285	NRAS
chr1	115252179	115252359	NRAS
chr1	115256410	115256609	NRAS
chr1	115258660	115258791	NRAS
chr1	155870168	155870419	RIT1
chr1	155874091	155874303	RIT1
chr1	155874383	155874411	RIT1
chr1	155874511	155874605	RIT1
chr1	155880230	155880307	RIT1
chr1	155880436	155880605	RIT1
chr1	155880658	155880686	RIT1
chr10	112327564	112327599	SMC3
chr10	112328685	112328781	SMC3
chr10	112333454	112333513	SMC3
chr10	112335083	112335171	SMC3
chr10	112337168	112337260	SMC3
chr10	112337582	112337682	SMC3
chr10	112338375	112338474	SMC3
chr10	112340651	112340789	SMC3
chr10	112341670	112341866	SMC3
chr10	112342309	112342410	SMC3
chr10	112343131	112343316	SMC3
chr10	112343588	112343730	SMC3
chr10	112343930	112344164	SMC3
chr10	112349352	112349476	SMC3
chr10	112349639	112349759	SMC3
chr10	112350159	112350340	SMC3
chr10	112350738	112350900	SMC3
chr10	112352820	112352991	SMC3
chr10	112356145	112356318	SMC3
chr10	112357886	112358058	SMC3
chr10	112359401	112359580	SMC3
chr10	112360186	112360314	SMC3
chr10	112360769	112360898	SMC3
chr10	112361384	112361652	SMC3

chr10	112361713	112361946	SMC3
chr10	112362221	112362433	SMC3
chr10	112362572	112362770	SMC3
chr10	112362931	112363058	SMC3
chr10	112363978	112364070	SMC3
chr11	32410593	32410735	WT1
chr11	32413507	32413620	WT1
chr11	32414201	32414311	WT1
chr11	32417792	32417963	WT1
chr11	32421483	32421600	WT1
chr11	32438025	32438096	WT1
chr11	32439108	32439210	WT1
chr11	32449491	32449614	WT1
chr11	32450032	32450175	WT1
chr11	32452065	32452095	WT1
chr11	32456235	32456901	WT1
chr11	108098341	108098433	ATM
chr11	108098492	108098625	ATM
chr11	108099894	108100060	ATM
chr11	108106386	108106571	ATM
chr11	108114669	108114855	ATM
chr11	108115504	108115763	ATM
chr11	108117680	108117864	ATM
chr11	108119649	108119839	ATM
chr11	108121417	108121809	ATM
chr11	108122553	108122768	ATM
chr11	108123533	108123649	ATM
chr11	108124530	108124776	ATM
chr11	108126931	108127077	ATM
chr11	108128197	108128343	ATM
chr11	108129702	108129812	ATM
chr11	108137887	108138079	ATM
chr11	108139126	108139346	ATM
chr11	108141780	108141883	ATM
chr11	108141967	108142143	ATM
chr11	108143248	108143344	ATM
chr11	108143438	108143589	ATM
chr11	108150207	108150345	ATM
chr11	108151711	108151905	ATM
chr11	108153426	108153616	ATM
chr11	108154943	108155210	ATM
chr11	108158316	108158452	ATM
chr11	108159693	108159840	ATM
chr11	108160318	108160538	ATM
chr11	108163335	108163530	ATM

chr11	108164029	108164214	ATM
chr11	108165643	108165796	ATM
chr11	108168003	108168119	ATM
chr11	108170430	108170622	ATM
chr11	108172364	108172526	ATM
chr11	108173569	108173766	ATM
chr11	108175391	108175589	ATM
chr11	108178613	108178721	ATM
chr11	108180876	108181052	ATM
chr11	108183127	108183235	ATM
chr11	108186539	108186648	ATM
chr11	108186727	108186850	ATM
chr11	108188089	108188258	ATM
chr11	108190670	108190795	ATM
chr11	108192017	108192157	ATM
chr11	108196026	108196281	ATM
chr11	108196774	108196962	ATM
chr11	108198361	108198495	ATM
chr11	108199737	108199975	ATM
chr11	108200930	108201158	ATM
chr11	108202160	108202294	ATM
chr11	108202595	108202774	ATM
chr11	108203478	108203637	ATM
chr11	108204602	108204705	ATM
chr11	108205685	108205846	ATM
chr11	108206561	108206698	ATM
chr11	108213938	108214108	ATM
chr11	108216459	108216645	ATM
chr11	108217995	108218102	ATM
chr11	108224482	108224617	ATM
chr11	108225527	108225611	ATM
chr11	108235798	108235955	ATM
chr11	108236041	108236245	ATM
chr11	119148461	119148560	cbl_e7
chr11	119148870	119149013	cbl_e8
chr11	119149214	119149429	cbl_e9
chr12	11803051	11803104	ETV6
chr12	11905373	11905523	ETV6
chr12	11992063	11992248	ETV6
chr12	11993384	11993427	ETV6
chr12	12006350	12006505	ETV6
chr12	12022347	12022913	ETV6
chr12	12037368	12037531	ETV6
chr12	12038849	12038970	ETV6
chr12	12043864	12043990	ETV6

chr12	22811942	22812094	etnk1_e3
chr12	25362718	25362855	KRAS
chr12	25368364	25368504	KRAS
chr12	25378537	25378717	KRAS
chr12	25380157	25380356	KRAS
chr12	25398197	25398328	KRAS
chr12	111855939	111856691	SH2B3
chr12	111884546	111884668	SH2B3
chr12	111884735	111884847	SH2B3
chr12	111884918	111885033	SH2B3
chr12	111885123	111885358	SH2B3
chr12	111885449	111885641	SH2B3
chr12	111885776	111886116	SH2B3
chr12	112856905	112856939	PTPN11
chr12	112884069	112884212	PTPN11
chr12	112888111	112888326	PTPN11
chr12	112890988	112891201	PTPN11
chr12	112892357	112892494	PTPN11
chr12	112893743	112893877	PTPN11
chr12	112910737	112910854	PTPN11
chr12	112915444	112915544	PTPN11
chr12	112915650	112915829	PTPN11
chr12	112919867	112920019	PTPN11
chr12	112924268	112924447	PTPN11
chr12	112926236	112926324	PTPN11
chr12	112926817	112926989	PTPN11
chr12	112939937	112940070	PTPN11
chr12	112942488	112942578	PTPN11
chr13	28592598	28592732	flt3_e20
chr13	28601219	28601384	flt3_e17
chr13	28602309	28602431	flt3_e16
chr13	28608018	28608357	flt3_e14_15
chr13	33222899	33223027	PDS5B
chr13	33225930	33226154	PDS5B
chr13	33232365	33232472	PDS5B
chr13	33232560	33232678	PDS5B
chr13	33233280	33233427	PDS5B
chr13	33241890	33241991	PDS5B
chr13	33247342	33247503	PDS5B
chr13	33249970	33250106	PDS5B
chr13	33252961	33253076	PDS5B
chr13	33258004	33258170	PDS5B
chr13	33261260	33261432	PDS5B
chr13	33262582	33262716	PDS5B
chr13	33268349	33268451	PDS5B

chr13	33270980	33271049	PDS5B
chr13	33273856	33274016	PDS5B
chr13	33275449	33275585	PDS5B
chr13	33281060	33281186	PDS5B
chr13	33284072	33284253	PDS5B
chr13	33306227	33306371	PDS5B
chr13	33309298	33309477	PDS5B
chr13	33315207	33315296	PDS5B
chr13	33316718	33316875	PDS5B
chr13	33320104	33320248	PDS5B
chr13	33327459	33327684	PDS5B
chr13	33329969	33330104	PDS5B
chr13	33332214	33332367	PDS5B
chr13	33332661	33332801	PDS5B
chr13	33333755	33333838	PDS5B
chr13	33334702	33334868	PDS5B
chr13	33338616	33338742	PDS5B
chr13	33344248	33344708	PDS5B
chr13	33344781	33344913	PDS5B
chr13	33347316	33347472	PDS5B
chr13	33349144	33349200	PDS5B
chr15	90631808	90631989	IDH2
chr17	1553942	1554260	PRPF8
chr17	1554391	1554614	PRPF8
chr17	1554697	1554857	PRPF8
chr17	1554931	1555092	PRPF8
chr17	1556825	1556987	PRPF8
chr17	1557060	1557320	PRPF8
chr17	1558633	1558847	PRPF8
chr17	1559675	1559869	PRPF8
chr17	1559931	1560065	PRPF8
chr17	1561536	1561685	PRPF8
chr17	1561809	1562067	PRPF8
chr17	1562640	1562852	PRPF8
chr17	1563124	1563305	PRPF8
chr17	1563715	1563882	PRPF8
chr17	1563981	1564131	PRPF8
chr17	1564276	1564466	PRPF8
chr17	1564554	1564710	PRPF8
chr17	1564894	1565094	PRPF8
chr17	1565189	1565457	PRPF8
chr17	1576364	1576501	PRPF8
chr17	1576640	1576871	PRPF8
chr17	1577029	1577196	PRPF8
chr17	1577725	1577984	PRPF8

chr17	1578435	1578643	PRPF8
chr17	1578903	1579116	PRPF8
chr17	1579211	1579358	PRPF8
chr17	1579490	1579674	PRPF8
chr17	1579788	1580015	PRPF8
chr17	1580259	1580476	PRPF8
chr17	1580848	1580998	PRPF8
chr17	1581801	1581956	PRPF8
chr17	1582045	1582185	PRPF8
chr17	1582300	1582510	PRPF8
chr17	1582574	1582714	PRPF8
chr17	1582892	1583103	PRPF8
chr17	1584009	1584135	PRPF8
chr17	1584212	1584358	PRPF8
chr17	1584761	1584994	PRPF8
chr17	1585103	1585342	PRPF8
chr17	1585412	1585597	PRPF8
chr17	1586816	1587005	PRPF8
chr17	1587755	1587875	PRPF8
chr17	7565246	7565342	TP53
chr17	7569513	7569572	TP53
chr17	7572916	7573018	TP53
chr17	7573916	7574043	TP53
chr17	7576526	7576594	TP53
chr17	7576614	7576667	TP53
chr17	7576842	7576936	TP53
chr17	7577008	7577165	TP53
chr17	7577488	7577618	TP53
chr17	7578166	7578299	TP53
chr17	7578360	7578564	TP53
chr17	7579301	7579600	TP53
chr17	7579689	7579731	TP53
chr17	7579828	7579922	TP53
chr17	29422215	29422397	NF1
chr17	29482990	29483154	NF1
chr17	29486017	29486121	NF1
chr17	29490193	29490404	NF1
chr17	29496898	29497025	NF1
chr17	29508429	29508517	NF1
chr17	29508717	29508813	NF1
chr17	29509515	29509693	NF1
chr17	29527429	29527623	NF1
chr17	29528044	29528187	NF1
chr17	29528418	29528513	NF1
chr17	29533247	29533399	NF1

chr17	29541458	29541613	NF1
chr17	29546012	29546146	NF1
chr17	29548857	29549018	NF1
chr17	29550451	29550595	NF1
chr17	29552102	29552278	NF1
chr17	29553442	29553712	NF1
chr17	29554225	29554319	NF1
chr17	29554530	29554634	NF1
chr17	29556032	29556493	NF1
chr17	29556842	29557002	NF1
chr17	29557267	29557410	NF1
chr17	29557849	29557953	NF1
chr17	29559080	29559217	NF1
chr17	29559707	29559909	NF1
chr17	29560009	29560241	NF1
chr17	29562618	29562800	NF1
chr17	29562925	29563049	NF1
chr17	29575991	29576147	NF1
chr17	29579945	29580028	NF1
chr17	29585351	29585530	NF1
chr17	29586039	29586157	NF1
chr17	29587376	29587543	NF1
chr17	29588718	29588885	NF1
chr17	29592236	29592367	NF1
chr17	29652827	29653280	NF1
chr17	29654506	29654867	NF1
chr17	29657303	29657526	NF1
chr17	29661845	29662059	NF1
chr17	29663340	29663501	NF1
chr17	29663642	29663942	NF1
chr17	29664375	29664610	NF1
chr17	29664824	29664908	NF1
chr17	29665009	29665167	NF1
chr17	29665711	29665833	NF1
chr17	29667512	29667673	NF1
chr17	29670016	29670163	NF1
chr17	29676127	29676279	NF1
chr17	29677190	29677346	NF1
chr17	29679264	29679442	NF1
chr17	29683467	29683610	NF1
chr17	29683967	29684118	NF1
chr17	29684276	29684397	NF1
chr17	29685487	29685650	NF1
chr17	29685976	29686043	NF1
chr17	29687494	29687739	NF1

chr17	29701020	29701183	NF1
chr17	40359570	40359752	STAT5B_e16
chr17	40362183	40362325	STAT5B_e15
chr17	40467752	40467828	STAT3_e21
chr17	40468796	40468929	STAT3_e20
chr17	40469189	40469252	STAT3_e19
chr17	58677765	58678257	PPM1D
chr17	58700871	58701120	PPM1D
chr17	58711203	58711348	PPM1D
chr17	58725242	58725453	PPM1D
chr17	58733949	58734212	PPM1D
chr17	58740345	58740923	PPM1D
chr17	74732575	74733246	srsf2_e1
chr18	42531884	42531962	setbp1_e4hotspot
chr19	13054521	13054733	calr_e9
chr19	33792144	33793420	cebpa_e1
chr2	25457137	25457299	DNMT3A
chr2	25458565	25458704	DNMT3A
chr2	25459794	25459884	DNMT3A
chr2	25461988	25462094	DNMT3A
chr2	25462348	25462392	DNMT3A
chr2	25463160	25463329	DNMT3A
chr2	25463498	25463609	DNMT3A
chr2	25464420	25464586	DNMT3A
chr2	25466756	25466861	DNMT3A
chr2	25467013	25467217	DNMT3A
chr2	25467398	25467531	DNMT3A
chr2	25468111	25468211	DNMT3A
chr2	25468878	25468943	DNMT3A
chr2	25469018	25469188	DNMT3A
chr2	25469478	25469655	DNMT3A
chr2	25469909	25470037	DNMT3A
chr2	25470449	25470628	DNMT3A
chr2	25470895	25471131	DNMT3A
chr2	25472515	25472603	DNMT3A
chr2	25475052	25475076	DNMT3A
chr2	25497799	25497966	DNMT3A
chr2	25498358	25498422	DNMT3A
chr2	25505246	25505590	DNMT3A
chr2	25522997	25523122	DNMT3A
chr2	25536771	25536863	DNMT3A
chr2	198266460	198266618	sf3b1_e16
chr2	198266703	198266860	sf3b1_e15
chr2	198267274	198267556	sf3b1_e14
chr2	198267667	198267765	sf3b1_e13

chr2	198268303	198268494	sf3b1_e12
chr2	209113060	209113159	IDH1
chr20	31021078	31025148	asxl1_e11-12
chr20	33866989	33867019	EIF6
chr20	33867358	33867560	EIF6
chr20	33867734	33867931	EIF6
chr20	33868446	33868642	EIF6
chr20	33871968	33872300	EIF6
chr20	57484399	57484484	gnas_e8
chr20	57484570	57484640	gnas_e9
chr21	36164421	36164917	RUNX1
chr21	36171587	36171769	RUNX1
chr21	36193954	36194003	RUNX1
chr21	36206696	36206908	RUNX1
chr21	36228699	36228754	RUNX1
chr21	36231760	36231885	RUNX1
chr21	36252843	36253020	RUNX1
chr21	36259129	36259419	RUNX1
chr21	36261963	36262044	RUNX1
chr21	36265211	36265270	RUNX1
chr21	36421128	36421206	RUNX1
chr21	44514749	44514904	u2af1_e6
chr21	44524419	44524518	u2af1_e2
chr3	128198260	128200171	GATA2_E7
chr3	128200652	128202858	GATA2_E5-6
chr3	128204560	128205929	GATA2_E3-4
chr3	128206544	128207383	GATA2_E1-2
chr4	55589744	55589870	kit_e8
chr4	55593576	55593714	kit_e11
chr4	55599230	55599364	kit_e17
chr4	106111616	106111653	TET2
chr4	106155043	106158607	TET2
chr4	106162485	106162600	TET2
chr4	106163980	106164094	TET2
chr4	106164716	106164945	TET2
chr4	106180765	106180936	TET2
chr4	106182905	106183015	TET2
chr4	106190756	106190914	TET2
chr4	106193710	106194085	TET2
chr4	106196194	106197686	TET2
chr5	1295100	1295488	TERT_promoter
chr5	148876405	148876433	CSNK1A1
chr5	148884994	148885168	CSNK1A1
chr5	148886579	148886706	CSNK1A1
chr5	148889431	148889526	CSNK1A1

chr5	148891346	148891445	CSNK1A1
chr5	148892622	148892782	CSNK1A1
chr5	148897346	148897450	CSNK1A1
chr5	148899842	148899961	CSNK1A1
chr5	148904597	148904744	CSNK1A1
chr5	148929627	148929754	CSNK1A1
chr5	148930394	148930537	CSNK1A1
chr5	170837525	170837575	npm1_e11
chr7	66453350	66453496	sbds_e5
chr7	66456115	66456299	sbds_e4
chr7	66458198	66458411	sbds_e3
chr7	66459127	66459338	sbds_e2
chr7	66460269	66460412	sbds_e1
chr7	140453069	140453199	braf_e15
chr7	148504727	148504808	EZH2
chr7	148506152	148506257	EZH2
chr7	148506391	148506492	EZH2
chr7	148507414	148507516	EZH2
chr7	148508706	148508822	EZH2
chr7	148511040	148511239	EZH2
chr7	148511995	148512141	EZH2
chr7	148512587	148512648	EZH2
chr7	148513765	148513880	EZH2
chr7	148514303	148514493	EZH2
chr7	148514958	148515219	EZH2
chr7	148516677	148516789	EZH2
chr7	148523535	148523734	EZH2
chr7	148524245	148524368	EZH2
chr7	148525821	148525982	EZH2
chr7	148526809	148526950	EZH2
chr7	148529715	148529852	EZH2
chr7	148533903	148534102	EZH2
chr7	148543551	148543700	EZH2
chr7	148544263	148544400	EZH2
chr8	117859728	117859940	RAD21
chr8	117861174	117861278	RAD21
chr8	117862846	117863016	RAD21
chr8	117864176	117864345	RAD21
chr8	117864777	117864957	RAD21
chr8	117866473	117866717	RAD21
chr8	117868394	117868537	RAD21
chr8	117868874	117869020	RAD21
chr8	117869495	117869722	RAD21
chr8	117870580	117870707	RAD21
chr8	117874069	117874189	RAD21

chr8	117875358	117875508	RAD21
chr8	117878814	117878978	RAD21
chr8	128748829	128748879	MYC
chr8	128750483	128751275	MYC
chr8	128752631	128753214	MYC
chr9	5069919	5070058	JAK2_e12
chr9	5073692	5073791	JAK2_e14
chr9	5078300	5078450	JAK2_e16
chrX	15339617	15339904	PIGA
chrX	15342776	15343003	PIGA
chrX	15343131	15343284	PIGA
chrX	15344025	15344178	PIGA
chrX	15349327	15350062	PIGA
chrX	15353612	15353645	PIGA
chrX	15808608	15808669	ZRSR2
chrX	15809046	15809146	ZRSR2
chrX	15817984	15818086	ZRSR2
chrX	15819386	15819647	ZRSR2
chrX	15821800	15821929	ZRSR2
chrX	15822223	15822330	ZRSR2
chrX	15826345	15826404	ZRSR2
chrX	15827312	15827451	ZRSR2
chrX	15833789	15834023	ZRSR2
chrX	15836699	15836775	ZRSR2
chrX	15838319	15838449	ZRSR2
chrX	15840843	15841375	ZRSR2
chrX	39909158	39909254	BCOR
chrX	39911351	39911663	BCOR
chrX	39913124	39913305	BCOR
chrX	39913498	39913596	BCOR
chrX	39914610	39914776	BCOR
chrX	39916397	39916584	BCOR
chrX	39921381	39921656	BCOR
chrX	39921988	39922334	BCOR
chrX	39922850	39923215	BCOR
chrX	39923578	39923862	BCOR
chrX	39930215	39930422	BCOR
chrX	39930879	39930953	BCOR
chrX	39931591	39934443	BCOR
chrX	39935696	39935795	BCOR
chrX	39937086	39937192	BCOR
chrX	53407013	53407117	SMC1A
chrX	53407530	53407661	SMC1A
chrX	53407928	53408018	SMC1A
chrX	53409142	53409314	SMC1A

chrX	53409416	53409591	SMC1A
chrX	53410007	53410184	SMC1A
chrX	53421687	53421818	SMC1A
chrX	53423136	53423310	SMC1A
chrX	53423381	53423547	SMC1A
chrX	53426500	53426662	SMC1A
chrX	53430487	53430614	SMC1A
chrX	53430698	53430835	SMC1A
chrX	53431876	53432091	SMC1A
chrX	53432166	53432333	SMC1A
chrX	53432414	53432614	SMC1A
chrX	53432692	53432898	SMC1A
chrX	53435982	53436210	SMC1A
chrX	53436341	53436444	SMC1A
chrX	53438700	53438861	SMC1A
chrX	53438934	53439213	SMC1A
chrX	53439839	53440098	SMC1A
chrX	53440171	53440395	SMC1A
chrX	53441696	53441829	SMC1A
chrX	53441919	53442128	SMC1A
chrX	53448834	53448977	SMC1A
chrX	53449430	53449559	SMC1A
chrX	123156467	123156531	STAG2
chrX	123159679	123159778	STAG2
chrX	123160404	123160478	STAG2
chrX	123164800	123164985	STAG2
chrX	123171366	123171483	STAG2
chrX	123176408	123176505	STAG2
chrX	123179003	123179228	STAG2
chrX	123181193	123181365	STAG2
chrX	123182844	123182938	STAG2
chrX	123184025	123184169	STAG2
chrX	123184960	123185079	STAG2
chrX	123185154	123185254	STAG2
chrX	123189967	123190095	STAG2
chrX	123191705	123191837	STAG2
chrX	123195063	123195201	STAG2
chrX	123195610	123195734	STAG2
chrX	123196741	123196854	STAG2
chrX	123196955	123197065	STAG2
chrX	123197687	123197911	STAG2
chrX	123199715	123199806	STAG2
chrX	123200014	123200122	STAG2
chrX	123200195	123200296	STAG2
chrX	123202403	123202516	STAG2

chrX	123204988	123205183	STAG2
chrX	123210171	123210331	STAG2
chrX	123211796	123211918	STAG2
chrX	123215219	123215388	STAG2
chrX	123217260	123217409	STAG2
chrX	123220386	123220630	STAG2
chrX	123224414	123224624	STAG2
chrX	123224693	123224824	STAG2
chrX	123227857	123228004	STAG2
chrX	123229211	123229309	STAG2
chrX	123234413	123234457	STAG2
chrX	129139197	129139303	BCORL1
chrX	129146543	129146654	BCORL1
chrX	129146915	129150199	BCORL1
chrX	129154949	129155135	BCORL1
chrX	129156861	129156962	BCORL1
chrX	129158954	129159364	BCORL1
chrX	129162599	129162846	BCORL1
chrX	129171331	129171518	BCORL1
chrX	129173101	129173267	BCORL1
chrX	129184681	129184779	BCORL1
chrX	129185824	129186001	BCORL1
chrX	129189818	129190121	BCORL1
chrX	133511637	133511795	PHF6
chrX	133512024	133512146	PHF6
chrX	133527520	133527674	PHF6
chrX	133527928	133527992	PHF6
chrX	133547507	133547697	PHF6
chrX	133547842	133548006	PHF6
chrX	133549035	133549262	PHF6
chrX	133551188	133551346	PHF6
chrX	133559220	133559370	PHF6

Supplementary Table 3: Human EIF6 *in silico* interface binding per conservation score, MSA data, variety of residues in other species and predicted binding energy per mutation.

Postion	Sequence	Conserv. Score	MSA data (of 150)	Residue Variety	Mutation in SDS cohort	Mut Residue	# Mutations in Cohort	ddGmut (kcal/mol)	Surface (boundary or core)	RPL23_interface	ddGbind EIF6-RPL23(kcal/mol)
2	A	-0.77	119	C,V,T,A,S							
3	V	0.984	121	R,S,Q,L,V,T,H,N,I	Y	D	1	7.43	core		
4	R	-0.905	122	K,G,R							
5	A	1.587	126	A,G,I,C,V,L,T	Y	D	1	4.22	core		
6	S	0.294	129	K,N,E,G,H,S,D,R,M,A,T,L,Q							
7	F	-0.09	130	C,Y,H,F							
8	E	-0.848	131	Q,K,D,E							
9	N	0.044	132	G,A,K,N,S,T	Y	D	1	0.29	surface	TRUE	2.49645
10	N	0.015	132	S,C,V,T,G,H,K,N							
11	C	0.27	133	T,C,D,P,S,N,K,H							
12	E	0.526	134	E,R,D	Y	K	2	-4.00	surface	TRUE	1.713336
13	I	0.397	134	I,L,V	Y	N	1	6.74	core		
14	G	-0.997	134	G	Y	D	3	15.31	core		
15	C	-0.846	135	A,C,V	Y	Y	1	1.31	surface	TRUE	3.803585
16	F	-0.781	135	F,Y,V	Y	S	1	10.33	core	TRUE	1.792967
17	A	0.764	136	T,C,L,S,A,G	Y	P	2	32.53	boundary		
18	K	0.837	136	S,P,R,M,A,T,L,K,N,H							
19	L	-1.021	136	L	Y	F	1	20.24	core		
20	T	-0.958	136	T,S,A	Y	I	1	18.53	core		
21	N	-0.911	136	L,N,S							
22	T	1.193	136	S,A,R,T,W,N,K,G							
23	Y	-0.812	136	L,Y,F	Y	H,N	2	5.061, 7.563	boundary		
24	C	-0.627	137	A,C							
25	L	-0.393	136	L,V,I,S,M	Y	R	1	10.81	boundary		
26	V	-0.059	136	T,V,C,L,I,A							
27	A	0.866	136	T,L,C,S,P,G,A	Y	V	1	3.27	boundary	TRUE	0.393157
28	I	1.181	136	I,Y,H,S,P,M,A,R,T,V,L,Q							
29	G	0.174	136	F,N,G,H,Q,C,S,A	Y	V	1	0.43	surface		
30	G	1.623	137	D,S,R,A,M,T,Q,V,N,G							
31	S	-0.512	136	N,I,F,X,T,C,S,A							
32	E	0.802	138	H,E,N,K,Q,T,A,M,S							
33	N	-0.185	139	A,S,V,Q,T,H,E,G,I,N							
34	F	-0.529	140	L,S,F,Y,G							
35	Y	0.244	140	S,A,T,V,L,N,I,F,H,Y							
36	S	-0.69	143	N,H,Y,G,S,D,R,T	Y	I,T	2	6.63	surface		
37	V	1.074	142	T,L,V,S,A,M,I,G							
38	F	-0.166	142	V,L,Y,I,F							
39	E	-0.853	142	E,R,A,D							
40	G	2.016	142	G,N,I,C,Q,T,A,M,S,D							
41	E	-0.408	143	K,I,E,H,S,D,R,A,Q							
42	L	-0.66	143	F,I,L,V							
43	S	1.653	142	V,Q,A,R,S,P,Y,E,G,N,F,K							
44	D	1.123	143	Q,H,E,G,P,D,S							
45	T	1.145	142	V,Q,L,T,A,R,S,H,Y,E,N,I							
46	I	-0.38	143	L,V,M,F,I	Y	S	2	9.35	boundary		
47	P	-0.987	144	P	Y	L	1	3.42	boundary		
48	V	-0.572	143	T,V,L,I							
49	V	0.96	143	C,V,F,I							
50	H	0.309	143	F,K,N,E,H,Y,T,Q,D,S,P,R,M							
51	A	1.044	143	S,A,T,C,V	Y	P	1	9.23	boundary		
52	S	-0.369	143	S,N,A,T,L,Q							
53	I	-0.244	144	L,V,T,I							
54	A	0.716	144	G,A,F,S,N,Q,C							
55	G	0.259	145	E,G,A,D,N,S,T							
56	C	0.177	145	I,N,T,C,V,L,S,A	Y	R,Y	2	2.398, 3.73	boundary	TRUE	2.946562, 1.906111
57	R	-0.5	145	T,S,R,A,K,I,G,H	Y	P	1	3.13	surface	TRUE	0.383353
58	I	-0.284	145	I,F,V,L,T,A,M,S							
59	I	0.2	146	I,F,V,L	Y	M	1	6.13	core		
60	G	-1	146	G							
61	R	-0.405	146	H,R,S,K,T	Y	H	4	9.38	surface	TRUE	0.551924
62	M	0.111	147	I,A,M,T,L,V							
63	C	0.848	147	V,C,T,A,P,S							
64	V	0.466	149	L,V,C,T,A,P,S,I							
65	G	-0.648	148	A,G	Y	V	1	8.24	boundary		
66	N	-0.998	148	K,N	Y	K,S	2	19.77, 6.135	core		
67	R	0.209	148	C,T,R,N,S,K	Y	W	1	10.25	surface		
68	H	0.475	148	R,H,K,N							
69	G	-1	148	G	Y	S,V	5	10.233, 30.976	boundary		
70	L	-0.904	148	V,L,I							
71	L	-0.591	148	I,V,L							
72	V	-0.443	149	V,L,I	Y	G	1	8.21	boundary		
73	P	-0.884	149	P,S	Y	L	1	2.91	boundary	TRUE	0.445027
74	N	1.054	149	N,I,F,H,Y,G,T,Q,S,M,A							
75	N	1.055	149	G,I,N,Q,T,A,M,S,D							
76	T	-0.587	149	C,V,T,A,I							
77	T	-0.466	149	T,Q,L,N,S,F,G							
78	D	-1.026	149	D	Y	Y	1	2.71	surface		
79	Q	-0.158	149	T,L,Q,V,S,P,R,A,M,N,G,E,H							
80	E	-0.917	150	E,D,Q	Y	G,K	2	14.045, 15.146	core		
81	L	-0.483	150	L,V,M,F,I,D	Y	R	1	-1.02	surface		
82	Q	-0.232	150	L,Q,S,D,R,A,M,K,I,E							
83	H	-0.689	150	H,Y,N,F,L,Q							
84	I	0.188	150	L,F,I,M							
85	R	-0.661	150	K,R,M,T,V	Y	H	1	9.15	surface		
86	N	-0.919	150	D,N							
87	S	-0.271	150	C,T,A,H,N,S							
88	L	-0.429	150	M,I,V,L							
89	P	-0.988	149	P							
90	D	-0.016	149	Q,E,G,S,D							
91	T	3.274	149	K,N,E,G,H,D,S,R,A,T,Q							
92	V	-0.638	148	A,I,V	Y	E	1	10.50	boundary		
93	Q	1.621	149	A,R,S,V,C,L,Q,T,E,G,I,N,K							
94	I	0.304	149	L,V,I							
95	R	-0.067	149	Q,V,R,M,E,H,X,Y,K							
96	R	-0.871	150	S,K,R,C	Y	W	13	11.20	boundary		
97	V	0.201	150	T,V,C,L,I,A,G							
98	E	-0.058	150	E,D,N,Q							
99	E	-0.971	148	D,X,E	Y	K,Q	3	17.519, 12.373	boundary		
100	R	-0.594	148	P,K,X,R							
101	L	-0.713	148	I,F,X,Y,M,L							
102	S	-0.874	148	T,C,N,S,A,X							
103	A	-0.95	148	S,A,X	Y	P	2	5.79	boundary	TRUE	8.973251
104	L	-1.023	148	L,X							
105	G	-1	148	G,X	Y	D	2	14.30	core		
106	N	-1.037	148	X,N	Y	S	20	1.12	surface	TRUE	2.557597
107	V	-0.319	150	A,M,T,L,C,V,I,N							
108	T	-0.425	150	V,T,I,D							
109	T	0.272	150	S,A,T,L,C,V							
110	C	0.146	150	N,S,A,E,T,C,V							
111	N	-0.999	150	S,N	Y	E,D,H,K,S	7	3.253, 0.511,10.775,12.494,3.39	boundary		
112	D	-0.974	150	H,D	Y	N,V	4	7.235, 12.438	surface		
113	Y	-0.259	150	S,N,K,H,Y,R	Y	C	1	3.24	surface		
114	V	-0.625	150	M,A,F,I,C,V,T							
115	A	-0.766	150	I,S,A,G							
116	L	-0.563	150	I,M,L							
117	V	0.357	149	M,A,I,L,C,V,T							
118	H	-0.905	149	N,R,H							
119	P	0.355	149	S,I,P,H,A,T,L							
120	D	-0.444	149	Q,D,S,K,E	Y	V	1	7.37	surface		
121	L	0.569	149	F,I,M,A,T,L,V							
122	D	-0.701	149	E,G,S,D	Y	V	2	6.30	surface		
123	R	1.066	149	T,V,L,Q,S,P,D,A,M,R,K,E							
124	E	-0.682	149	D,G,E,R,A							
125	T	-0.923	149	T,S							
126	E	-0.635	149	K,E,A,Q,V							
127	E	-0.481	149	E,Y,K,N,D,Q	Y	K	1	-0.83	surface		
128	I	-0.187	149	A,M,F,I,D,L,V							
129	L	-0.409	149	I,V,L	Y	Q	1	7.84	core		
130	A	0.61	149	Q,V,C,T,M,A,S,G,E,K,I							
131	D	-0.809	149	E,D							
132	V	-0.126	149	V,T,A,I,S							
133	L	-1.023	149	L	Y	P	1	30.22	core		
134	K	1.492	149	Q,G,R,N,D,K							
135	V	-0.898	150	V,L,T,A	Y	M	6	12.38	core		
136	E	-0.972	150	D,E							
137	V	-0.822	150	V,I,P,A							
138	F	-0.794	150	F,I,Y							
139	R	-0.592	150	C,K,P,R,G,A							
140	Q	0.068	150	T,V,C,L,Q,S,A,M,N,H,G,E							
141	T	-0.403	150	T,V,S,A,M,I,K,E							

142	V	0.785	150	I,V,L							
143	A	-0.286	150	C,V,S,A,G							
144	D	2.368	150	G,E,K,N,Q,T,S,D							
145	Q	-0.233	150	V,Q,N,H,Y,E							
146	V	0.6	150	I,E,G,T,V,L,S,P,A							
147	L	-0.908	150	M,I,L							
148	V	-0.751	150	A,I,V,T							
149	G	-1.001	150	G	Y	E	1	16.03	core		
150	S	-0.549	150	T,A,S,N	Y	R	1	-2.64	surface	TRUE	1.788935
151	Y	-0.565	150	Y,R,N,F							
152	C	0.466	150	G,T,L,C,V,S,M,A							
153	V	1.672	150	K,I,G,H,S,R,A,T,Q,C,V							
154	F	1.157	150	L,I,F,M							
155	S	-0.202	150	T,N,S							
156	N	-0.921	150	N,S,A							
157	Q	0.442	150	T,V,L,Q,S,A,R,N,K							
158	G	-0.911	150	E,G	Y	R,V	3	16.804, 46.324	boundary		
159	G	0.244	150	C,G,A	Y	E,R	2	35.351, 51.238	boundary		
160	L	0.188	150	L,W,V,I,M	Y	P	1	21.17	core		
161	V	-0.894	150	V,L,I,F	Y	G	1	9.83	core		
162	H	-0.667	150	I,H,E,S,D,A,R,Q							
163	P	-0.085	149	V,A,P,S							
164	K	2.983	149	A,M,R,S,D,C,Q,L,Y,H,G,N,K							
165	T	-0.636	149	H,A,S,I,V,T							
166	S	0.631	149	Q,T,K,S,N,P							
167	I	1.345	150	K,I,L,V,T,R,A,M,S,P	Y	N	1	1.12	surface		
168	E	1.176	150	D,S,R,A,T,Q,V,K,N,E							
169	D	0.431	150	E,Y,I,N,Q,L,V,T,A,D,S							
170	Q	0.063	150	I,K,M,R,T,L,Q	Y	P	1	24.93	boundary		
171	D	0.299	150	R,S,D,V,E,G,H,Y,K,N							
172	E	-0.805	150	E,S,D,Q							
173	L	-0.78	150	N,S,I,V,L	Y	P,R	2	24.974, 10.306	core		
174	S	-0.698	150	P,S,A							
175	S	-0.407	150	T,L,Q,S,D,A,N,E							
176	L	-0.785	150	L,Q,V,C,M							
177	L	-0.545	150	I,R,C,V,L	Y	R	1	30.74	core		
178	Q	-0.711	150	K,H,Y,E,G,T,Q,S							
179	V	-0.077	150	L,V,G,I,S	Y	D,G	2	13.868, 10.733	core		
180	P	-0.677	150	H,P,S							
181	L	0.033	150	M,I,F,V,L	Y	H,P	4	13.449, 19.496	core		
182	V	-0.272	149	C,V,L,T,A,M,Y,I							
183	A	-0.949	148	T,S,A							
184	G	-0.826	147	S,G	Y	E,R	2	4.272, 0.439	boundary		
185	T	-0.847	147	T,S							
186	V	-0.148	147	I,V,L							
187	N	-1.037	147	N	Y	I,K	2	7.072, 2.446	boundary		
188	R	-0.759	147	Q,C,K,S,R,H	Y	P	3	30.52	surface		
189	G	-1	147	G							
190	S	-0.906	147	S,N,L,C							
191	E	1.528	146	Q,A,D,P,S,E,H,N							
192	V	-0.368	146	T,L,V,C,S,A,M,N							
193	I	0.607	146	L,V,I							
194	A	0.185	146	A,X,G,S	Y	T	2	-0.19	core		
195	A	-0.185	147	S,G,A	Y	D	2	7.33	surface	TRUE	1.975551
196	G	-1	147	G	Y	E,R	4	0.439, 4.272	core		
197	M	1.061	147	M,I,C,V,L							
198	V	-0.056	147	C,V,L,T,A,M,I	Y	M	1	5.24	core		
199	V	-0.662	147	C,V,L,A,S							
200	N	-1.037	147	N	Y	I,T	2	11.76	boundary		
201	D	-1.026	147	D							
202	W	-0.293	147	F,Y,C,W							
203	C	2.759	147	S,M,A,T,L,Q,C,V,F,I							
204	A	-0.366	147	G,A,S,L	Y	D	1	11.11	core		
205	F	-0.753	147	F,I,M,V							
206	C	0.789	147	S,A,T,V,C	Y	R,Y	2	16.593, 11.859	core		
207	G	-1	147	G	Y	D,R	2	14.712, 23.569	boundary		
208	L	1.723	148	F,N,I,E,Y,T,Q,L,V,S,R,M,A							
209	D	0.025	148	K,N,D,S,E,H,A							
210	T	-0.962	147	T,S	Y	I	3	2.45	core		
211	T	-0.999	147	T,S							
212	S	-0.07	147	A,G,S,T							
213	T	-0.662	147	A,H,P,I,V,T							
214	E	-1.025	147	E							
215	L	0.674	147	M,I,L,V	Y	P	1	27.06	boundary		
216	S	-0.311	147	T,Q,S,N,G,A							
217	V	-0.848	147	A,I,L,V							
218	V	0.483	147	V,L,I,M							
219	E	-0.8	147	K,N,D,E							
220	S	-0.028	146	N,K,G,E,T,Q,S,A,R							
221	V	-0.059	147	A,I,L,V,T							
222	F	-0.614	147	Y,F,L							
223	K	0.281	146	A,G,R,N,K,Q							
224	L	-0.257	146	P,I,F,L							
225	N	1.585	146	H,E,G,N,I,K,V,Q,T,A,R,S,D							
226	E	3.165	144	T,Q,D,S,R,A,K,N,G,E,H							
227	A	3.032	142	N,K,H,Y,G,E,S,P,M,A,R,T,V,Q,L							
228	Q	3.221	142	P,S,D,R,A,M,T,Q,L,K,I,N,E,G,H							
229	P	2.021	140	S,P,D,A,T,Q,N,G,H							
230	S	2.678	139	K,I,N,G,E,T,Q,V,D,S,R,A,M							
231	T	3.282	138	E,G,Y,H,F,K,N,Q,L,V,T,R,A,D,S							
232	I	0.551	136	G,I,N,F,V,L,T,M,A,R,P							
233	A	2.124	133	V,Q,L,T,M,A,S,D,G,N,I,F,K							
234	T	3.286	134	H,E,G,N,K,A,D,S,V,Q,L,T							
235	S	3.289	134	K,N,E,G,H,T,Q,V,S,D,A,M							
236	M	0.303	133	D,M,V,L,I,N,K,F							
237	R	-0.175	134	Q,N,K,H,R							
238	D	1.344	134	P,D,S,R,A,T,Q,K,N,E,G							
239	S	0.494	133	T,Q,P,D,S,A,N,Y,G							
240	L	-0.057	133	T,L,V,F,I,M							
241	I	-0.159	133	F,I,L,V							
242	D	-0.071	130	Q,E,D							
243	S	0.917	121	K,N,G,H,D,S,R,A,T,Q							
244	L	0.581	67	F,I,Y,M,L,V							
245	T	-0.833	15	T							

Supplementary Table 4: Primers used in qPCR and site directed mutagenesis of EIF6.

Gene or EIF6 SDM amino acid change	primer sequence
hEIF6 Forward	CCTTGGACCCAGCTTTCTT
hEIF6 Reverse across V5 tag	TACTGTCGAGACCTAGGAGTG
GAPDH Forward	TGCACCACCAACTGCTTAGC
GAPDH Reverse	GGCATGGACTGTGGTCATGAG
CDKN1A Forward	CAGCATGACAGATTTCTACC
CDKN1A Reverse	CAGGGTATGTACATGAGGAG
I13NForward	AACTGTGAGAaCGGCTGCTTTG
I13NReverse	GTTCTCGAACGAAGCTCG
R67WForward	TGTGGGGAACtGGCACGGTCT
R67WReverse	CACATGCGCCCGATGATG
G69SForward	GAACAGGCACaGTCTCCTGGTACCC
G69SReverse	CCCACACACATGCGCCCG
P73RForward	CTCCTGGTACgCAACAATACCACC
P73RReverse	ACCGTGCCTGTTCCCCAC
R96WForward	GCAGATTAGGTGGGTGGAGGA
R96WReverse	ACTGTGTCTGGGAGGCTG
N106Forward	GCCTTGGGCAGTGTCACCACC
N106Reverse	TGAGAGCCGCTCCTCCAC
A194TForward	TGAGGTGATTaCTGCTGGGATG
A194TReverse	CTGCCTCGGTTCACAGTC
G196RForward	GATTGCTGCTaGGATGGTGGTG
G196RReverse	ACCTCACTGCCTCGGTTC

Supplementary Table 5: Single cell sequencing coordinates

chromosome	position start	position stop
chr2	25457136	25457349
chr2	25458539	25458743
chr2	25459801	25460005
chr2	25461899	25462103
chr2	25463127	25463327
chr2	25463465	25463671
chr2	25464369	25464585
chr2	25466697	25466860
chr2	25467026	25467230
chr2	25467400	25467614
chr2	25468072	25468306
chr2	25468723	25468904
chr2	25469027	25469218
chr2	25469485	25469694
chr2	25469895	25470109
chr2	25470457	25470627
chr2	25470904	25471114
chr2	25472488	25472689
chr2	25474735	25474963
chr2	25475065	25475238
chr2	25497765	25497972
chr2	25498273	25498447
chr2	25505317	25505550
chr2	25522949	25523150
chr2	25536735	25536925
chr4	106155105	106155319
chr4	106155368	106155560
chr4	106155630	106155857
chr4	106155904	106156137
chr4	106156272	106156502
chr4	106156609	106156809
chr4	106156890	106157081
chr4	106157171	106157363
chr4	106157414	106157601
chr4	106157656	106157772
chr4	106157835	106158047
chr4	106158107	106158311
chr4	106158389	106158599
chr4	106162450	106162651
chr4	106163977	106164165
chr4	106164725	106164932
chr4	106180744	106180929
chr4	106182858	106183005

chr4	106190709	106190903
chr4	106193540	106193738
chr4	106193794	106194016
chr4	106194057	106194281
chr4	106196203	106196436
chr4	106196481	106196669
chr4	106196713	106196872
chr4	106196922	106197107
chr4	106197213	106197443
chr4	106197502	106197682
chr5	148899836	148900029
chr5	148904571	148904738
chr7	13010156	13010358
chr7	25435592	25435755
chr7	34230275	34230449
chr7	50545021	50545221
chr7	52518590	52518774
chr7	66116028	66116230
chr7	66456179	66456393
chr7	66459178	66459405
chr7	68003177	68003365
chr7	70132929	70133091
chr7	75322548	75322746
chr7	80864445	80864629
chr7	82857047	82857229
chr7	88441439	88441593
chr7	93225093	93225275
chr7	94484213	94484392
chr17	3444147	3444340
chr17	5906390	5906565
chr17	6202241	6202420
chr17	7572919	7573112
chr17	7573884	7574087
chr17	7576499	7576700
chr17	7576779	7576969
chr17	7577015	7577175
chr17	7577405	7577614
chr17	7578098	7578294
chr17	7578353	7578564
chr17	7579314	7579547
chr17	7579698	7579929
chr17	8934656	8934808
chr17	9806662	9806862
chr17	10916913	10917087
chr17	13960334	13960492

chr17	58740315	58740481
chr17	58740530	58740735
chr17	58740781	58740969
chr20	6047840	6048015
chr20	8699445	8699615
chr20	16075717	16075920
chr20	16702487	16702683
chr20	18990856	18991015
chr20	20765111	20765246
chr20	33488766	33488964
chr20	33867359	33867564
chr20	33867741	33867943
chr20	33868448	33868631
chr20	33868679	33868849
chr20	33871887	33872067
chr20	33872135	33872326
chr20	36816080	36816262
chr20	37493143	37493312
chr20	39271393	39271576
chr20	39950650	39950826

Supplementary Table 6: Somatic Mutations detected in SDS Cohort

Cohort ID	Timepoint	chrom	position start	position end	coordinates	reference	variant	gene	cdna	aa	result	reads1	reads2	var_freq
1	A	20	33868509	33868509	20:33868509-33868509	T	C	EIF6	c.A317G	p.N106S	nonsynonymous SNV	806	12	0.0147
1	A	17	7577094	7577094	17:7577094-7577094	G	A	TP53	c.C844T	p.R282W	nonsynonymous SNV	845	5	0.0059
5	A	20	33868509	33868509	20:33868509-33868509	T	C	EIF6	c.A317G	p.N106S	nonsynonymous SNV	942	10	0.0105
5	A	20	33868573	33868573	20:33868573-33868573	G	-	EIF6	c.253delC	p.R85fs	frameshift deletion	923	7	0.0075
5	A	20	33868633	33868633	20:33868633-33868633	C	G	EIF6	c.194-1G>C		splicing	648	3	0.0046
5	A	20	33872219	33872219	20:33872219-33872219	A	-	EIF6	c.72delT	p.C24fs	frameshift deletion	873	22	0.0246
5	A	17	7577534	7577534	17:7577534-7577534	C	G	TP53	c.G747C	p.R249S	nonsynonymous SNV	990	3	0.003
5	A	17	7578236	7578236	17:7578236-7578236	A	G	TP53	c.T613C	p.Y205H	nonsynonymous SNV	1062	6	0.0056
5	A	17	7578508	7578508	17:7578508-7578508	C	T	TP53	c.G422A	p.C141Y	nonsynonymous SNV	1022	5	0.0049
8	A	20	33872187	33872188	20:33872187-33872188	0	-	EIF6	c.103_104del	p.Y35fs	frameshift deletion	942	17	0.0177
8	A	17	7577121	7577121	17:7577121-7577121	G	A	TP53	c.C817T	p.R273C	nonsynonymous SNV	837	84	0.0912
9	A	5	148904673	148904673	5:148904673-148904673	C	T	CSNK1A1	c.G292A	p.E98K	nonsynonymous SNV	773	9	0.0115
9	B	5	148904673	148904673	5:148904673-148904673	C	T	CSNK1A1	c.G292A	p.E98K	nonsynonymous SNV	758	12	0.0156
10	A	17	7578190	7578190	17:7578190-7578190	T	C	TP53	c.A659G	p.Y220C	nonsynonymous SNV	786	3	0.0038
11	A	2	209113113	209113113	2:209113113-209113113	G	A	IDH1	c.C394T	p.R132C	nonsynonymous SNV	520	300	0.3659
13	Z	X	39922007	39922007	X:39922007-39922007	C	T	BCOR	c.G4165A	p.D1389N	nonsynonymous SNV	798	7	0.0087
13	2	X	39922007	39922007	X:39922007-39922007	C	T	BCOR	c.G4165A	p.D1389N	nonsynonymous SNV	990	5	0.005
13	1	20	33867759	33867759	20:33867759-33867759	G	A	EIF6	c.C475T	p.Q159X	stopgain	2347	5	0.0021
13	1	20	33867782	33867782	20:33867782-33867782	T	G	EIF6	c.A452C	p.Q151P	nonsynonymous SNV	2743	5	0.0018
13	1	20	33872183	33872183	20:33872183-33872183	C	A	EIF6	c.G108T	p.R36S	nonsynonymous SNV	2698	16	0.0059
13	Z	12	25398285	25398285	12:25398285-25398285	C	A	KRAS	c.G34T	p.G12C	nonsynonymous SNV	976	3	0.0031
13	1	17	58740651	58740651	17:58740651-58740651	G	-	PPM1D	c.1556delG	p.G519fs	frameshift deletion	2572	4	0.0016
13	1	17	7577121	7577121	17:7577121-7577121	G	A	TP53	c.C817T	p.R273C	nonsynonymous SNV	2435	3	0.0012
13	Z	17	7577124	7577124	17:7577124-7577124	C	T	TP53	c.G814A	p.V272M	nonsynonymous SNV	510	357	0.4118
13	1	17	7577124	7577124	17:7577124-7577124	C	T	TP53	c.G814A	p.V272M	nonsynonymous SNV	1962	414	0.1742
13	2	17	7577124	7577124	17:7577124-7577124	C	T	TP53	c.G814A	p.V272M	nonsynonymous SNV	632	474	0.4286
13	Z	17	7577152	7577152	17:7577152-7577152	A	-	TP53	c.786delT	p.G262fs	frameshift deletion	388	299	0.4352
13	1	17	7577152	7577152	17:7577152-7577152	A	-	TP53	c.786delT	p.G262fs	frameshift deletion	1516	323	0.1756
13	2	17	7577152	7577152	17:7577152-7577152	A	-	TP53	c.786delT	p.G262fs	frameshift deletion	479	380	0.4424
13	1	17	7577529	7577529	17:7577529-7577529	A	T	TP53	c.T752A	p.I251N	nonsynonymous SNV	2260	10	0.0044
13	1	17	7577539	7577539	17:7577539-7577539	G	A	TP53	c.C742T	p.R248W	nonsynonymous SNV	2403	3	0.0012
13	1	17	7577568	7577568	17:7577568-7577568	C	T	TP53	c.G713A	p.C238Y	nonsynonymous SNV	2159	4	0.0018
13	1	17	7578475	7578475	17:7578475-7578475	G	A	TP53	c.C455T	p.P152L	nonsynonymous SNV	2813	3	0.0011
13	1	17	7578496	7578496	17:7578496-7578496	A	T	TP53	c.T434A	p.L145Q	nonsynonymous SNV	2937	3	0.001
13	1	17	7578535	7578535	17:7578535-7578535	T	C	TP53	c.A395G	p.K132R	nonsynonymous SNV	2356	14	0.0059
13	Z	21	44514777	44514777	21:44514777-44514777	T	C	U2AF1	c.A470G	p.Q157R	nonsynonymous SNV	537	373	0.4099
13	1	21	44514777	44514777	21:44514777-44514777	T	C	U2AF1	c.A470G	p.Q157R	nonsynonymous SNV	1829	389	0.1754
13	2	21	44514777	44514777	21:44514777-44514777	T	C	U2AF1	c.A470G	p.Q157R	nonsynonymous SNV	685	459	0.4012
14	A	20	33868509	33868509	20:33868509-33868509	T	C	EIF6	c.A317G	p.N106S	nonsynonymous SNV	1059	4	0.0038
14	B	20	33868509	33868509	20:33868509-33868509	T	C	EIF6	c.A317G	p.N106S	nonsynonymous SNV	1278	6	0.0047
14	A	20	33871990	33871990	20:33871990-33871990	C	T	EIF6	c.G301A	p.A101T	nonsynonymous SNV	812	21	0.0252
14	B	20	33871990	33871990	20:33871990-33871990	C	T	EIF6	c.G301A	p.A101T	nonsynonymous SNV	912	23	0.0246
14	Z	1	115256529	115256529	1:115256529-115256529	T	C	NRAS	c.A182G	p.Q61R	nonsynonymous SNV	715	3	0.0042
14	Z	12	112888199	112888199	12:112888199-112888199	C	T	PTPN11	c.C215T	p.A72V	nonsynonymous SNV	1171	4	0.0034
14	Z	12	112926888	112926888	12:112926888-112926888	G	C	PTPN11	c.G1508C	p.G503A	nonsynonymous SNV	1269	15	0.0117
14	A	17	7573996	7573996	17:7573996-7573996	A	G	TP53	c.T1031C	p.L344P	nonsynonymous SNV	785	199	0.2022
14	B	17	7573996	7573996	17:7573996-7573996	A	G	TP53	c.T1031C	p.L344P	nonsynonymous SNV	953	302	0.2406
14	C	17	7573996	7573996	17:7573996-7573996	A	G	TP53	c.T1031C	p.L344P	nonsynonymous SNV	789	398	0.3353
14	Z	17	7573996	7573996	17:7573996-7573996	A	G	TP53	c.T1031C	p.L344P	nonsynonymous SNV	723	554	0.4338
14	A	17	7577538	7577538	17:7577538-7577538	C	T	TP53	c.G743A	p.R248Q	nonsynonymous SNV	590	259	0.3051
14	B	17	7577538	7577538	17:7577538-7577538	C	T	TP53	c.G743A	p.R248Q	nonsynonymous SNV	685	347	0.3362
14	C	17	7577538	7577538	17:7577538-7577538	C	T	TP53	c.G743A	p.R248Q	nonsynonymous SNV	636	317	0.3326
14	Z	17	7577538	7577538	17:7577538-7577538	C	T	TP53	c.G743A	p.R248Q	nonsynonymous SNV	614	507	0.4523
14	A	17	7578406	7578406	17:7578406-7578406	C	T	TP53	c.G524A	p.R175H	nonsynonymous SNV	1075	8	0.0074
14	B	17	7578406	7578406	17:7578406-7578406	C	T	TP53	c.G524A	p.R175H	nonsynonymous SNV	1241	7	0.0056
15	B	20	33872244	33872244	20:33872244-33872244	A	G	EIF6	c.T47C	p.F16S	nonsynonymous SNV	1003	3	0.003
17	L	20	33867001	33867001	20:33867001-33867001	C	G	EIF6	c.G680C	p.X227S	stoploss	897	16	0.0175
17	K	20	33867001	33867001	20:33867001-33867001	C	G	EIF6	c.G680C	p.X227S	stoploss	1081	19	0.0173
17	E	20	33867001	33867001	20:33867001-33867001	C	G	EIF6	c.G680C	p.X227S	stoploss	958	13	0.0134
17	I	20	33867001	33867001	20:33867001-33867001	C	G	EIF6	c.G680C	p.X227S	stoploss	946	30	0.0307
17	H	20	33867001	33867001	20:33867001-33867001	C	G	EIF6	c.G680C	p.X227S	stoploss	869	12	0.0136
17	C	20	33867001	33867001	20:33867001-33867001	C	G	EIF6	c.G680C	p.X227S	stoploss	1436	40	0.0271
17	A	20	33867001	33867001	20:33867001-33867001	C	G	EIF6	c.G680C	p.X227S	stoploss	905	39	0.0413
17	B	20	33867001	33867001	20:33867001-33867001	C	G	EIF6	c.G680C	p.X227S	stoploss	1050	15	0.0141
17	J	20	33867001	33867001	20:33867001-33867001	C	G	EIF6	c.G680C	p.X227S	stoploss	940	16	0.0167
17	G	20	33867001	33867001	20:33867001-33867001	C	G	EIF6	c.G680C	p.X227S	stoploss	720	22	0.0296
17	D	20	33867001	33867001	20:33867001-33867001	C	G	EIF6	c.G680C	p.X227S	stoploss	1073	14	0.0129
17	A	20	33867547	33867547	20:33867547-33867547	C	T	EIF6	c.G493A	p.G165R	nonsynonymous SNV	746	3	0.004
17	G	20	33867547	33867547	20:33867547-33867547	C	T	EIF6	c.G493A	p.G165R	nonsynonymous SNV	604	6	0.0098
17	B	20	33871979	33871979	20:33871979-33871979	C	T	EIF6	c.G312A	p.W104X	stopgain	766	3	0.0039
17	A	20	33872246	33872246	20:33872246-33872246	G	-	EIF6	c.45delC	p.C15fs	frameshift deletion	953	22	0.0226
17	B	20	33872246	33872246	20:33872246-33872246	G	-	EIF6	c.45delC	p.C15fs	frameshift deletion	1033	3	0.0029
17	C	20	33872246	33872246	20:33872246-33872246	G	-	EIF6	c.45delC	p.C15fs	frameshift deletion	1349	3	0.0022
17	D	20	33872246	33872246	20:33872246-33872246	G	-	EIF6	c.45delC	p.C15fs	frameshift deletion	1006	3	0.003
17	E	20	33872246	33872246	20:33872246-33872246	G	-	EIF6	c.45delC	p.C15fs	frameshift deletion	958	6	0.0062
17	K	20	33872246	33872246	20:33872246-33872246	G	-	EIF6	c.45delC	p.C15fs	frameshift deletion	1080	6	0.0055
17	L	20	33872246	33872246	20:33872246-33872246	G	-	EIF6	c.45delC	p.C15fs	frameshift deletion	858	3	0.0035
17	A	20	33872250	33872250	20:33872250-33872250	-	CGAT	EIF6	c.40_41insATCG	p.G14fs	frameshift insertion	965	11	0.0113
17	B	20	33872250	33872250	20:33872250-33872250	-	CGAT	EIF6	c.40_41insATCG	p.G14fs	frameshift insertion	1023	6	0.0058
17	C	20	33872250	33872250	20:33872250-33872250	-	CGAT	EIF6	c.40_41insATCG	p.G14fs	frameshift insertion	1352	8	0.0059
17	D	20	33872250	33872250	20:33872250-33872250	-	CGAT	EIF6	c.40_41insATCG	p.G14fs	frameshift insertion	1005	4	0.004
17	G	20	33872250	33872250	20:33872250-33872250	-	CGAT	EIF6	c.40_41insATCG	p.G14fs	frameshift insertion	736	3	0.0041
17	I	20	33872250	33872250	20:33872250-33872250	-	CGAT	EIF6	c.40_41insATCG	p.G14fs	frameshift insertion	878	3	0.0034
17	K	20	33872250	33872250	20:33872250-33872250	-	CGAT	EIF6	c.40_41insATCG	p.G14fs	frameshift insertion	1076	4	0.0037
17	G	12	111884636	111884636	12:111884636-111884636	A	G	SH2B3	c.A812G	p.N271S	nonsynonymous SNV	732	6	0.0081
17	L	17	7577108	7577108	17:7577108-7577108	C	T	TP53	c.G830A	p.C277Y	nonsynonymous SNV	909	12	0.013
17	C	17	7577121	7577121	17:7577121-7577121	G	A	TP53	c.C817T	p.R273C	nonsynonymous SNV	1407	4	0.0028
17	J	17	7577121	7577121	17:7577121-7577121	G	A	TP53	c.C817T	p.R273C	nonsynonymous SNV	849	3	0.0035
17	K	17	7578395	7578395	17:7578395-7578395	G	A	TP53	c.C535T	p.H179Y	nonsynonymous SNV	1101	3	0.0027
17	E	17	7578406	7578406	17:7578406-7578406	C	T	TP53	c.G524A	p.R175H	nonsynonymous SNV	1021	4	0.0039
17	G	17	7578406	7578406	17:7578406-7578406	C	T	TP53	c.G524A	p.R175H	nonsynonymous SNV	823	15	0.0179
17	L	17	7578406	7578406	17:7578406-7578406	C	T	TP53	c.G524A	p.R175H	nonsynonymous SNV	961	8	0.0083
18	A	5	148892636	148892636	5:148892636-148892636	T	C	CSNK1A1	c.A677G	p.Q226R	nonsynonymous SNV	714	3	0.0042
18	B	5	148892636	148892636	5:148892636-148892636	T	C	CSNK1A1	c.A677G	p.Q226R	nonsynonymous SNV	727	3	0.0041
18	C	5	148892636	148892636	5:148892636-148892636	T	C	CSNK1A1	c.A677G	p.Q226R	nonsynonymous SNV	1153	3	0.0026
18	B	20	33867510	33867510	20:33867510-33867510	C	T	EIF6	c.G530A	p.G177E	nonsynonymous SNV	1062	4	0.0038
18	C	20	33867510	33867510	20:33867510-33867510	C	T	EIF6	c.G530A	p.G177E	nonsynonymous SNV	1573	7	0.0044
18	A	20	33867866	33867869	20:33867866-33867869	0	-	EIF6	c.365_368del	p.T122fs	frameshift deletion	1037	13	0.0124
18	B	20	33867866	33867869	20:33867866-33867869	0	-	EIF6	c.365_368del	p.T122fs	frameshift deletion	1052	14	0.0131
18	C	20	33867866	33867869	20:33867866-33867869	0	-	EIF6	c.365_368del	p.T122fs	frameshift deletion	1527	15	0.0097
18	C	20	33872059	33872060	20:33872059-33872060	0	-	EIF6	c.231_232del	p.C77fs	frameshift deletion	1251	9	0.0071
18	C	17	1565303	1565303	17:1565303-1565303	T	C	PRPF8	c.A3919G	p.M1307V	nonsynonymous SNV	1591	5	0.0031
18	A	17	7577022	7577022	17:7577022-7577022	G	A	TP53	c.C916T	p.R306X	stopgain	937	3	0.0032
18	B	17	7577022	7577022	17:7577022-7577022	G	A	TP53	c.C916T	p.R306X	stopgain	991	5	0.005
18	C	17	7577022	7577022	17:7577022-7577022	G	A	TP53	c.C916T	p.R306X	stopgain	1444	6	0.0041
18	A	17	7577508	7577508	17:7577508-7577508	T	C	TP53	c.A773G	p.E258G	nonsynonymous SNV	755	24	0.0308

18	B	17	7577508	7577508	17:7577508-7577508	T	C	TP53	c.A773G	p.E258G	nonsynonymous SNV	793	31	0.0376
18	C	17	7577508	7577508	17:7577508-7577508	T	C	TP53	c.A773G	p.E258G	nonsynonymous SNV	1049	58	0.0524
18	A	17	7577539	7577539	17:7577539-7577539	G	A	TP53	c.C742T	p.R248W	nonsynonymous SNV	949	8	0.0084
18	B	17	7577539	7577539	17:7577539-7577539	G	A	TP53	c.C742T	p.R248W	nonsynonymous SNV	1012	10	0.0098
18	C	17	7577539	7577539	17:7577539-7577539	G	A	TP53	c.C742T	p.R248W	nonsynonymous SNV	1384	16	0.0114
18	A	17	7578393	7578393	17:7578393-7578393	A	T	TP53	c.T537A	p.H179Q	nonsynonymous SNV	852	158	0.1564
18	B	17	7578393	7578393	17:7578393-7578393	A	T	TP53	c.T537A	p.H179Q	nonsynonymous SNV	909	137	0.131
18	C	17	7578393	7578393	17:7578393-7578393	A	T	TP53	c.T537A	p.H179Q	nonsynonymous SNV	1343	208	0.1341
20	B	20	33867888	33867888	20:33867888-33867888	C	T	EIF6	c.G346A	p.V116M	nonsynonymous SNV	824	6	0.0072
20	B	20	33868584	33868584	20:33868584-33868584	A	C	EIF6	c.T242G	p.L81R	nonsynonymous SNV	945	3	0.0032
21	2	20	33868509	33868509	20:33868509-33868509	T	C	EIF6	c.A317G	p.N106S	nonsynonymous SNV	1294	5	0.0038
21	A	20	33868531	33868531	20:33868531-33868531	C	T	EIF6	c.G295A	p.E99K	nonsynonymous SNV	709	4	0.0056
21	A	17	7577543	7577543	17:7577543-7577543	C	A	TP53	c.G738T	p.M246I	nonsynonymous SNV	693	3	0.0043
21	1	17	7577543	7577543	17:7577543-7577543	C	A	TP53	c.G738T	p.M246I	nonsynonymous SNV	1164	3	0.0026
21	2	17	7577543	7577543	17:7577543-7577543	C	A	TP53	c.G738T	p.M246I	nonsynonymous SNV	1158	5	0.0043
21	2	17	7578212	7578212	17:7578212-7578212	G	C	TP53	c.C637G	p.R213G	nonsynonymous SNV	1014	3	0.0029
23	D	20	33867368	33867368	20:33867368-33867368	C	T	EIF6	c.671+1G>A		splicing	1319	4	0.003
23	E	20	33867368	33867368	20:33867368-33867368	C	T	EIF6	c.671+1G>A		splicing	970	4	0.0041
23	G	20	33867368	33867368	20:33867368-33867368	C	T	EIF6	c.671+1G>A		splicing	682	7	0.0102
23	D	20	33867468	33867468	20:33867468-33867468	G	A	EIF6	c.C572T	p.T191I	nonsynonymous SNV	1876	6	0.0032
23	G	20	33867534	33867534	20:33867534-33867534	C	G	EIF6	c.G506C	p.R169P	nonsynonymous SNV	899	3	0.0033
23	G	20	33867916	33867916	20:33867916-33867916	T	-	EIF6	c.318delA	p.T106fs	frameshift deletion	754	3	0.004
23	G	20	33868582	33868582	20:33868582-33868582	G	A	EIF6	c.C244T	p.Q82X	stopgain	1060	3	0.0028
23	E	20	33872073	33872073	20:33872073-33872073	A	C	EIF6	c.T218G	p.V73G	nonsynonymous SNV	1243	4	0.0032
23	G	20	33872073	33872073	20:33872073-33872073	A	C	EIF6	c.T218G	p.V73G	nonsynonymous SNV	791	3	0.0038
23	E	17	1565301	1565301	17:1565301-1565301	C	A	PRPF8	c.G3921T	p.M1307I	nonsynonymous SNV	1344	3	0.0022
23	G	17	1565301	1565301	17:1565301-1565301	C	A	PRPF8	c.G3921T	p.M1307I	nonsynonymous SNV	949	5	0.0052
23	A	17	7577568	7577568	17:7577568-7577568	C	A	TP53	c.G713T	p.C238F	nonsynonymous SNV	832	3	0.0036
23	D	17	7579349	7579349	17:7579349-7579349	A	C	TP53	c.T338G	p.F113C	nonsynonymous SNV	1794	3	0.0017
24	D	20	33868587	33868587	20:33868587-33868587	T	C	EIF6	c.A239G	p.E80G	nonsynonymous SNV	1304	4	0.0031
26	A	20	33872035	33872035	20:33872035-33872035	A	C	EIF6	c.T256G	p.S86A	nonsynonymous SNV	386	108	0.2186
26	A	17	7577538	7577538	17:7577538-7577538	C	T	TP53	c.G743A	p.R248Q	nonsynonymous SNV	482	115	0.1926
26	A	17	7577539	7577539	17:7577539-7577539	G	A	TP53	c.C742T	p.R248W	nonsynonymous SNV	601	4	0.0066
26	A	17	7578403	7578403	17:7578403-7578403	C	T	TP53	c.G527A	p.C176Y	nonsynonymous SNV	658	3	0.0045
27	C	20	33867435	33867438	20:33867435-33867438	0	-	EIF6	c.602_605del	p.S201fs	frameshift deletion	853	3	0.0035
27	C	20	33867517	33867517	20:33867517-33867517	C	T	EIF6	c.G523A	p.A175T	nonsynonymous SNV	858	3	0.0035
27	B	20	33867535	33867535	20:33867535-33867535	G	A	EIF6	c.C505T	p.R169X	stopgain	771	3	0.0039
27	C	20	33867535	33867535	20:33867535-33867535	G	A	EIF6	c.C505T	p.R169X	stopgain	746	9	0.0119
27	D	20	33867535	33867535	20:33867535-33867535	G	A	EIF6	c.C505T	p.R169X	stopgain	773	17	0.0215
27	E	20	33867535	33867535	20:33867535-33867535	G	A	EIF6	c.C505T	p.R169X	stopgain	1142	19	0.0164
27	E	20	33868509	33868509	20:33868509-33868509	T	C	EIF6	c.A317G	p.N106S	nonsynonymous SNV	1300	5	0.0038
27	B	20	33868591	33868591	20:33868591-33868591	G	A	EIF6	c.C235T	p.Q79X	stopgain	821	4	0.0048
27	C	20	33868591	33868591	20:33868591-33868591	G	A	EIF6	c.C235T	p.Q79X	stopgain	847	3	0.0035
27	D	20	33868591	33868591	20:33868591-33868591	G	A	EIF6	c.C235T	p.Q79X	stopgain	891	3	0.0034
27	B	20	33868633	33868633	20:33868633-33868633	C	T	EIF6	c.194-1G>A		splicing	587	5	0.0084
27	C	20	33868633	33868633	20:33868633-33868633	C	T	EIF6	c.194-1G>A		splicing	581	6	0.0102
27	C	20	33872073	33872073	20:33872073-33872073	A	C	EIF6	c.T218G	p.V73G	nonsynonymous SNV	712	5	0.007
27	E	20	33872073	33872073	20:33872073-33872073	A	C	EIF6	c.T218G	p.V73G	nonsynonymous SNV	1094	8	0.0073
27	B	20	33872242	33872242	20:33872242-33872242	C	G	EIF6	c.G49C	p.A17P	nonsynonymous SNV	890	7	0.0078
27	C	20	33872242	33872242	20:33872242-33872242	C	G	EIF6	c.G49C	p.A17P	nonsynonymous SNV	757	10	0.013
27	D	20	33872242	33872242	20:33872242-33872242	C	G	EIF6	c.G49C	p.A17P	nonsynonymous SNV	902	6	0.0066
27	E	20	33872242	33872242	20:33872242-33872242	C	G	EIF6	c.G49C	p.A17P	nonsynonymous SNV	1195	5	0.0042
27	E	20	33872247	33872247	20:33872247-33872247	C	T	EIF6	c.G44A	p.C15Y	nonsynonymous SNV	1196	3	0.0025
27	D	20	33872251	33872251	20:33872251-33872251	C	-	EIF6	c.40delG	p.G14fs	frameshift deletion	904	8	0.0088
27	E	20	33872251	33872251	20:33872251-33872251	C	-	EIF6	c.40delG	p.G14fs	frameshift deletion	1194	5	0.0042
27	B	17	7573009	7573009	17:7573009-7573009	C	T	TP53	c.984-1G>A		splicing	590	14	0.0232
27	C	17	7573009	7573009	17:7573009-7573009	C	T	TP53	c.984-1G>A		splicing	524	19	0.035
27	D	17	7573009	7573009	17:7573009-7573009	C	T	TP53	c.984-1G>A		splicing	622	18	0.0281
27	E	17	7573009	7573009	17:7573009-7573009	C	T	TP53	c.984-1G>A		splicing	888	14	0.0155
27	B	17	7577539	7577539	17:7577539-7577539	G	A	TP53	c.C742T	p.R248W	nonsynonymous SNV	804	8	0.0099
27	C	17	7577539	7577539	17:7577539-7577539	G	A	TP53	c.C742T	p.R248W	nonsynonymous SNV	774	8	0.0102
27	D	17	7577539	7577539	17:7577539-7577539	G	A	TP53	c.C742T	p.R248W	nonsynonymous SNV	858	12	0.0138
27	E	17	7577539	7577539	17:7577539-7577539	G	A	TP53	c.C742T	p.R248W	nonsynonymous SNV	1184	3	0.0025
27	B	17	7577556	7577556	17:7577556-7577556	C	G	TP53	c.G725C	p.C242S	nonsynonymous SNV	826	3	0.0036
27	E	17	7577556	7577556	17:7577556-7577556	C	G	TP53	c.G725C	p.C242S	nonsynonymous SNV	1202	4	0.0033
27	C	17	7578395	7578395	17:7578395-7578395	G	A	TP53	c.C535T	p.H179Y	nonsynonymous SNV	910	6	0.0066
27	D	17	7578395	7578395	17:7578395-7578395	G	A	TP53	c.C535T	p.H179Y	nonsynonymous SNV	964	13	0.0133
27	E	17	7578395	7578395	17:7578395-7578395	G	A	TP53	c.C535T	p.H179Y	nonsynonymous SNV	1281	8	0.0062
27	E	17	7578406	7578406	17:7578406-7578406	C	T	TP53	c.G524A	p.R175H	nonsynonymous SNV	1303	4	0.0031
27	B	17	7578532	7578532	17:7578532-7578532	A	T	TP53	c.T398A	p.M133K	nonsynonymous SNV	685	29	0.0406
27	C	17	7578532	7578532	17:7578532-7578532	A	T	TP53	c.T398A	p.M133K	nonsynonymous SNV	714	34	0.0455
27	D	17	7578532	7578532	17:7578532-7578532	A	T	TP53	c.T398A	p.M133K	nonsynonymous SNV	742	25	0.0326
27	E	17	7578532	7578532	17:7578532-7578532	A	T	TP53	c.T398A	p.M133K	nonsynonymous SNV	986	47	0.0455
27	C	17	7579310	7579310	17:7579310-7579310	A	C	TP53	c.258+2T>G		splicing	676	3	0.0044
28	A	20	33867535	33867535	20:33867535-33867535	G	A	EIF6	c.C505T	p.R169X	stopgain	802	4	0.005
28	A	20	33872073	33872073	20:33872073-33872073	A	C	EIF6	c.T218G	p.V73G	nonsynonymous SNV	737	3	0.0041
28	A	20	33872242	33872242	20:33872242-33872242	C	G	EIF6	c.G49C	p.A17P	nonsynonymous SNV	812	3	0.0037
28	A	17	7573009	7573009	17:7573009-7573009	C	T	TP53	c.984-1G>A		splicing	623	15	0.0235
28	A	17	7577539	7577539	17:7577539-7577539	G	A	TP53	c.C742T	p.R248W	nonsynonymous SNV	800	8	0.0099
28	A	17	7578395	7578395	17:7578395-7578395	G	A	TP53	c.C535T	p.H179Y	nonsynonymous SNV	845	26	0.0299
28	A	17	7578532	7578532	17:7578532-7578532	A	T	TP53	c.T398A	p.M133K	nonsynonymous SNV	670	37	0.0523
28	A	17	7579310	7579310	17:7579310-7579310	A	C	TP53	c.258+2T>G		splicing	697	6	0.0085
29	D	20	33867481	33867481	20:33867481-33867481	A	G	EIF6	c.T559C	p.C187R	nonsynonymous SNV	1171	3	0.0026
29	B	20	33867755	33867755	20:33867755-33867755	A	T	EIF6	c.T479A	p.V160D	nonsynonymous SNV	639	4	0.0062
29	C	20	33867755	33867755	20:33867755-33867755	A	T	EIF6	c.T479A	p.V160D	nonsynonymous SNV	938	9	0.0095
29	D	20	33867755	33867755	20:33867755-33867755	A	T	EIF6	c.T479A	p.V160D	nonsynonymous SNV	901	9	0.0099
29	C	20	33868512	33868512	20:33868512-33868512	C	T	EIF6	c.G314A	p.G105D	nonsynonymous SNV	1375	10	0.0072
29	D	20	33868512	33868512	20:33868512-33868512	C	T	EIF6	c.G314A	p.G105D	nonsynonymous SNV	1089	8	0.0073
29	A	20	33868572	33868572	20:33868572-33868572	C	T	EIF6	c.G254A	p.R85H	nonsynonymous SNV	872	5	0.0057
29	C	20	33868572	33868572	20:33868572-33868572	C	T	EIF6	c.G254A	p.R85H	nonsynonymous SNV	1472	3	0.002
29	B	20	33871991	33871991	20:33871991-33871991	-	CC	EIF6	c.299_300insGG	p.G100fs	frameshift insertion	655	13	0.0195
29	C	20	33871991	33871991	20:33871991-33871991	-	CC	EIF6	c.299_300insGG	p.G100fs	frameshift insertion	927	30	0.0313
29	D	20	33871991	33871991	20:33871991-33871991	-	CC	EIF6	c.299_300insGG	p.G100fs	frameshift insertion	789	20	0.0247
29	B	20	33872048	33872048	20:33872048-33872048	-	C	EIF6	c.242dupG	p.S81fs	frameshift insertion	626	4	0.0063
29	C	20	33872048	33872048	20:33872048-33872048	-	C	EIF6	c.242dupG	p.S81fs	frameshift insertion	1058	7	0.0066
29	D	20	33872048	33872048	20:33872048-33872048	-	C	EIF6	c.242dupG	p.S81fs	frameshift insertion	870	4	0.0046
29	B	17	7578191	7578191	17:7578191-7578191	A	G	TP53	c.T658C	p.Y220H	nonsynonymous SNV	590	3	0.0051
30	E	20	33867749	33867749	20:33867749-33867749	A	G	EIF6	c.T485C	p.L162P	nonsynonymous SNV	674	3	0.0044
31	A	17	7577114	7577114	17:7577114-7577114	C	T	TP53	c.G824A	p.C275Y	nonsynonymous SNV	699	3	0.0043
31	A	17	7577141	7577141	17:7577141-7577141	C	T	TP53	c.G797A	p.G266E	nonsynonymous SNV	569	3	0.0052
31	B	17	7577141	7577141	17:7577141-7577141	C	T	TP53	c.G797A	p.G266E	nonsynonymous SNV	539	268	0.3321
31	A	17	7578395	7578395	17:7578395-7578395	G	T	TP53	c.C535A	p.H179N	nonsynonymous SNV	680	4	0.0058
31	B	17	7578525	7578525	17:7578525-7578525	G	C	TP53	c.C405G	p.C135W	nonsynonymous SNV	577	300	0.3421
33	A	20	33867491	33867491	20:33867491-33867491	C	T	EIF6	c.G549A	p.W183X	stopgain	1053	3	0.0028
33	B	20	33868491	33868491	20:33868491-33868491	T	A	EIF6	c.A335T	p.D112V	nonsynonymous SNV	992	4	0.004
33	A	20	33868553	33868553	20:33868553-33868553	T	-	EIF6	c.273delA	p.T91fs	frameshift deletion	1071	5	0.0046
33	A	17	7576570	7576570	17:7576570-7576570	T	-	TP53	c.531delA	p.L177fs	frameshift deletion	916	4	0.0043
33	A	17	7577548	7577548	17:7577548-7577548	C	T	TP53	c.G733A	p.G245S	nonsynonymous SNV	1028	4	0.0039

33	B	17	7578406	7578406	17:7578406-7578406	C	T	TP53	c.G524A	p.R175H	nonsynonymous SNV	1124	3	0.0027
34	A	20	33867535	33867535	20:33867535-33867535	G	A	EIF6	c.C505T	p.R169X	stopgain	777	6	0.0077
34	B	20	33867535	33867535	20:33867535-33867535	G	A	EIF6	c.C505T	p.R169X	stopgain	582	68	0.1046
34	C	20	33867535	33867535	20:33867535-33867535	G	A	EIF6	c.C505T	p.R169X	stopgain	561	40	0.0666
34	D	20	33867535	33867535	20:33867535-33867535	G	A	EIF6	c.C505T	p.R169X	stopgain	878	111	0.1122
34	E	20	33867535	33867535	20:33867535-33867535	G	A	EIF6	c.C505T	p.R169X	stopgain	836	92	0.0991
34	G	20	33867535	33867535	20:33867535-33867535	G	A	EIF6	c.C505T	p.R169X	stopgain	725	174	0.1935
34	A	20	33872021	33872021	20:33872021-33872021	C	G	EIF6	c.G151C	p.A51P	nonsynonymous SNV	705	6	0.0084
34	C	20	33872021	33872021	20:33872021-33872021	C	G	EIF6	c.G151C	p.A51P	nonsynonymous SNV	697	5	0.0071
34	D	20	33872224	33872224	20:33872224-33872224	A	T	EIF6	c.T67A	p.Y23N	nonsynonymous SNV	1093	3	0.0027
34	E	20	33872224	33872224	20:33872224-33872224	A	T	EIF6	c.T67A	p.Y23N	nonsynonymous SNV	1027	8	0.0077
34	E	12	112926852	112926852	12:112926852-112926852	C	T	PTPN11	c.C1472T	p.P491L	nonsynonymous SNV	1065	3	0.0028
35	A	20	33867402	33867402	20:33867402-33867402	A	-	EIF6	c.638delT	p.I213fs	frameshift deletion	714	5	0.007
35	A	20	33867905	33867905	20:33867905-33867905	A	-	EIF6	c.329delT	p.L110fs	frameshift deletion	597	15	0.0245
35	A	20	33868540	33868540	20:33868540-33868540	G	A	EIF6	c.C286T	p.R96W	nonsynonymous SNV	700	33	0.045
35	A	20	33868621	33868621	20:33868621-33868621	C	T	EIF6	c.G205A	p.G69S	nonsynonymous SNV	554	31	0.053
35	A	17	7577539	7577539	17:7577539-7577539	G	A	TP53	c.C742T	p.R248W	nonsynonymous SNV	680	10	0.0145
36	A	20	33867809	33867809	20:33867809-33867809	A	C	EIF6	c.T425G	p.V142G	nonsynonymous SNV	812	4	0.0049
36	3	20	33867809	33867809	20:33867809-33867809	A	C	EIF6	c.T425G	p.V142G	nonsynonymous SNV	1523	6	0.0039
36	A	20	33867888	33867888	20:33867888-33867888	C	T	EIF6	c.G346A	p.V116M	nonsynonymous SNV	697	5	0.0071
36	1	20	33867888	33867888	20:33867888-33867888	C	T	EIF6	c.G346A	p.V116M	nonsynonymous SNV	867	7	0.008
36	3	20	33867888	33867888	20:33867888-33867888	C	T	EIF6	c.G346A	p.V116M	nonsynonymous SNV	1205	11	0.009
36	3	20	33868588	33868588	20:33868588-33868588	C	A	EIF6	c.G238T	p.E80X	stopgain	1429	5	0.0035
36	3	17	7578406	7578406	17:7578406-7578406	C	T	TP53	c.G524A	p.R175H	nonsynonymous SNV	2178	3	0.0014
36	3	17	7578526	7578526	17:7578526-7578526	C	T	TP53	c.G404A	p.C135Y	nonsynonymous SNV	1920	3	0.0016
38	C	20	33868509	33868509	20:33868509-33868509	T	C	EIF6	c.A317G	p.N106S	nonsynonymous SNV	853	3	0.0035
38	G	20	33868509	33868509	20:33868509-33868509	T	C	EIF6	c.A317G	p.N106S	nonsynonymous SNV	1430	5	0.0035
38	A	20	33868621	33868621	20:33868621-33868621	C	T	EIF6	c.G205A	p.G69S	nonsynonymous SNV	543	11	0.0199
38	B	20	33868621	33868621	20:33868621-33868621	C	T	EIF6	c.G205A	p.G69S	nonsynonymous SNV	630	12	0.0187
38	C	20	33868621	33868621	20:33868621-33868621	C	T	EIF6	c.G205A	p.G69S	nonsynonymous SNV	666	8	0.0119
38	D	20	33868621	33868621	20:33868621-33868621	C	T	EIF6	c.G205A	p.G69S	nonsynonymous SNV	580	11	0.0186
38	E	20	33868621	33868621	20:33868621-33868621	C	T	EIF6	c.G205A	p.G69S	nonsynonymous SNV	583	11	0.0185
38	G	20	33868621	33868621	20:33868621-33868621	C	T	EIF6	c.G205A	p.G69S	nonsynonymous SNV	1093	13	0.0118
38	H	20	33868621	33868621	20:33868621-33868621	C	T	EIF6	c.G205A	p.G69S	nonsynonymous SNV	614	5	0.0081
38	I	20	33868621	33868621	20:33868621-33868621	C	T	EIF6	c.G205A	p.G69S	nonsynonymous SNV	703	7	0.0099
38	H	20	33872257	33872257	20:33872257-33872257	C	T	EIF6	c.G34A	p.E12K	nonsynonymous SNV	770	4	0.0052
38	B	12	112884198	112884198	12:112884198-112884198	G	C	PTPN11	c.G133C	p.V45L	nonsynonymous SNV	829	3	0.0036
38	C	17	7578455	7578455	17:7578455-7578455	C	G	TP53	c.G475C	p.A159P	nonsynonymous SNV	908	4	0.0044
38	H	17	7578455	7578455	17:7578455-7578455	C	G	TP53	c.G475C	p.A159P	nonsynonymous SNV	949	3	0.0032
39	G	2	25457191	25457191	2:25457191-25457191	C	T	DNMT3A	c.G2696A	p.R899H	nonsynonymous SNV	844	4	0.0047
40	1	20	31023408	31023408	20:31023408-31023408	C	T	ASXL1	c.C2893T	p.R965X	stopgain	1928	3	0.0016
40	1	5	148892675	148892675	5:148892675-148892675	G	A	CSNK1A1	c.C638T	p.A213V	nonsynonymous SNV	1864	5	0.0027
40	2	5	148892675	148892675	5:148892675-148892675	G	A	CSNK1A1	c.C638T	p.A213V	nonsynonymous SNV	1795	4	0.0022
40	2	20	33867517	33867517	20:33867517-33867517	C	T	EIF6	c.G523A	p.A175T	nonsynonymous SNV	1962	3	0.0015
40	1	20	33868509	33868509	20:33868509-33868509	T	C	EIF6	c.A317G	p.N106S	nonsynonymous SNV	2152	10	0.0046
40	2	20	33868509	33868509	20:33868509-33868509	T	C	EIF6	c.A317G	p.N106S	nonsynonymous SNV	2067	6	0.0029
40	1	12	22811995	22811995	12:22811995-22811995	A	G	ETNK1	c.A731G	p.N244S	nonsynonymous SNV	1660	144	0.0798
40	2	12	22811995	22811995	12:22811995-22811995	A	G	ETNK1	c.A731G	p.N244S	nonsynonymous SNV	1644	159	0.0882
40	2	3	128202795	128202795	3:128202795-128202795	C	T	GATA2	c.G925A	p.D309N	nonsynonymous SNV	1783	20	0.0111
41	A	20	33867535	33867535	20:33867535-33867535	G	A	EIF6	c.C505T	p.R169X	stopgain	856	3	0.0035
41	A	20	33867816	33867816	20:33867816-33867816	C	T	EIF6	c.G418A	p.G140R	nonsynonymous SNV	957	5	0.0052
41	A	20	33867888	33867888	20:33867888-33867888	C	T	EIF6	c.G346A	p.V116M	nonsynonymous SNV	883	4	0.0045
41	A	20	33868540	33868540	20:33868540-33868540	G	A	EIF6	c.C286T	p.R96W	nonsynonymous SNV	904	12	0.0131
41	B	20	33868589	33868589	20:33868589-33868589	C	-	EIF6	c.237delG	p.Q79fs	frameshift deletion	1356	3	0.0022
41	A	17	7577094	7577094	17:7577094-7577094	G	A	TP53	c.C844T	p.R282W	nonsynonymous SNV	851	5	0.0058
41	B	17	7578190	7578190	17:7578190-7578190	T	C	TP53	c.A659G	p.Y220C	nonsynonymous SNV	887	13	0.0144
41	C	17	7578190	7578190	17:7578190-7578190	T	C	TP53	c.A659G	p.Y220C	nonsynonymous SNV	734	4	0.0054
41	B	17	7578454	7578454	17:7578454-7578454	G	A	TP53	c.C476T	p.A159V	nonsynonymous SNV	1262	3	0.0024
42	C	20	33867841	33867841	20:33867841-33867841	G	C	EIF6	c.C393G	p.S131R	nonsynonymous SNV	1311	4	0.003
42	H	20	33872006	33872006	20:33872006-33872006	A	G	EIF6	c.T166C	p.C56R	nonsynonymous SNV	892	4	0.0045
42	D	17	7578190	7578190	17:7578190-7578190	T	C	TP53	c.A659G	p.Y220C	nonsynonymous SNV	779	6	0.0076
43	C	2	25457290	25457290	2:25457290-25457290	C	G	DNMT3A	c.2142-1G>C		splicing	1173	5	0.0042
43	A	20	33867773	33867773	20:33867773-33867773	A	G	EIF6	c.T461C	p.L154P	nonsynonymous SNV	892	10	0.0111
43	A	20	33867893	33867893	20:33867893-33867893	A	G	EIF6	c.T341C	p.L114P	nonsynonymous SNV	881	4	0.0045
43	B	20	33867893	33867893	20:33867893-33867893	A	G	EIF6	c.T341C	p.L114P	nonsynonymous SNV	840	10	0.0118
43	C	20	33867893	33867893	20:33867893-33867893	A	G	EIF6	c.T341C	p.L114P	nonsynonymous SNV	1580	14	0.0088
43	A	20	33867905	33867905	20:33867905-33867905	A	T	EIF6	c.T329A	p.L110Q	nonsynonymous SNV	810	3	0.0037
43	B	20	33868494	33868494	20:33868494-33868494	T	C	EIF6	c.A332G	p.N111S	nonsynonymous SNV	910	8	0.0087
43	C	20	33868494	33868494	20:33868494-33868494	T	C	EIF6	c.A332G	p.N111S	nonsynonymous SNV	1877	22	0.0116
43	C	20	33868620	33868620	20:33868620-33868620	C	A	EIF6	c.G206T	p.G69V	nonsynonymous SNV	1526	4	0.0026
43	C	17	7573925	7573925	17:7573925-7573925	A	C	TP53	c.983+2T>G		splicing	1357	3	0.0022
43	C	17	7577121	7577121	17:7577121-7577121	G	A	TP53	c.C817T	p.R273C	nonsynonymous SNV	1899	3	0.0016
43	A	17	7578392	7578392	17:7578392-7578392	C	T	TP53	c.G538A	p.E180K	nonsynonymous SNV	956	3	0.0031
43	B	17	7578392	7578392	17:7578392-7578392	C	T	TP53	c.G538A	p.E180K	nonsynonymous SNV	915	4	0.0044
43	C	17	7578392	7578392	17:7578392-7578392	C	T	TP53	c.G538A	p.E180K	nonsynonymous SNV	2076	3	0.0014
43	C	17	7578448	7578448	17:7578448-7578448	G	T	TP53	c.C482A	p.A161D	nonsynonymous SNV	2037	3	0.0015
43	A	17	7578517	7578517	17:7578517-7578517	G	A	TP53	c.C413T	p.A138V	nonsynonymous SNV	843	7	0.0082
43	B	17	7578517	7578517	17:7578517-7578517	G	A	TP53	c.C413T	p.A138V	nonsynonymous SNV	865	7	0.008
43	C	17	7578517	7578517	17:7578517-7578517	G	A	TP53	c.C413T	p.A138V	nonsynonymous SNV	1766	26	0.0145
45	E	20	33868478	33868478	20:33868478-33868478	C	-	EIF6	c.348delG	p.L116fs	frameshift deletion	762	3	0.0039
45	D	20	33868549	33868549	20:33868549-33868549	G	A	EIF6	c.C277T	p.Q93X	stopgain	980	5	0.0051
45	E	20	33868549	33868549	20:33868549-33868549	G	A	EIF6	c.C277T	p.Q93X	stopgain	860	3	0.0035
45	E	20	33868588	33868588	20:33868588-33868588	C	T	EIF6	c.G238A	p.E80K	nonsynonymous SNV	868	3	0.0034
45	C	20	33872274	33872274	20:33872274-33872274	G	T	EIF6	c.C17A	p.S6X	stopgain	876	4	0.0045
45	A	7	148543691	148543691	7:148543691-148543691	C	-	EZH2	c.118-1G>-		splicing	816	4	0.0049
45	A	12	112926887	112926887	12:112926887-112926887	G	C	PTPN11	c.G1507C	p.G503R	nonsynonymous SNV	2260	4	0.0018
45	C	12	112926887	112926887	12:112926887-112926887	G	C	PTPN11	c.G1507C	p.G503R	nonsynonymous SNV	896	3	0.0033
45	D	12	112926887	112926887	12:112926887-112926887	G	C	PTPN11	c.G1507C	p.G503R	nonsynonymous SNV	1009	5	0.0049
46	A	17	7577085	7577085	17:7577085-7577085	C	T	TP53	c.G853A	p.E285K	nonsynonymous SNV	854	4	0.0047
46	B	17	7577085	7577085	17:7577085-7577085	C	T	TP53	c.G853A	p.E285K	nonsynonymous SNV	850	4	0.0047
47	A	20	33868509	33868509	20:33868509-33868509	T	C	EIF6	c.A317G	p.N106S	nonsynonymous SNV	1571	6	0.0038
47	C	20	33868552	33868553	20:33868552-33868553	0	-	EIF6	c.273_274del	p.T91fs	frameshift deletion	971	3	0.0031
48	6	5	148904672	148904672	5:148904672-148904672	T	C	CSNK1A1	c.A293G	p.E98G	nonsynonymous SNV	1836	4	0.0022
48	6	5	148904673	148904673	5:148904673-148904673	C	T	CSNK1A1	c.G292A	p.E98K	nonsynonymous SNV	1832	7	0.0038
48	2	2	25463297	25463299	2:25463297-25463299	0	-	DNMT3A	c.2194_2196del	p.732_732del	nonframeshift deletion	1757	3	0.0017
48	4	2	25463297	25463299	2:25463297-25463299	0	-	DNMT3A	c.2194_2196del	p.732_732del	nonframeshift deletion	2214	5	0.0023
48	A	20	33868492	33868492	20:33868492-33868492	C	T	EIF6	c.G334A	p.D112N	nonsynonymous SNV	898	3	0.0033
48	6	20	33868492	33868492	20:33868492-33868492	C	T	EIF6	c.G334A	p.D112N	nonsynonymous SNV	2175	5	0.0023
48	1	20	33868509	33868509	20:33868509-33868509	T	C	EIF6	c.A317G	p.N106S	nonsynonymous SNV	1933	5	0.0026
48	2	20	33868509	33868509	20:33868509-33868509	T	C	EIF6	c.A317G	p.N106S	nonsynonymous SNV	2156	3	0.0014
48	4	20	33868509	33868509	20:33868509-33868509	T	C	EIF6	c.A317G	p.N106S	nonsynonymous SNV	2583	9	0.0035
48	6	20	33868509	33868509	20:33868509-33868509	T	C	EIF6	c.A317G	p.N106S	nonsynonymous SNV	2241	4	0.0018
48	A	20	33868573	33868573	20:33868573-33868573	G	-	EIF6	c.253delC	p.R85fs	frameshift deletion	895	6	0.0067
48	B	20	33868573	33868573	20:33868573-33868573	G	-	EIF6	c.253delC	p.R85fs	frameshift deletion	733	7	0.0095
48	1	20	33868573	33868573	20:33868573-33868573	G	-	EIF6	c.253delC	p.R85fs	frameshift deletion	1966	12	0.0061
48	2	20	33868573	33868573	20:33868573-33868573	G	-	EIF6	c.253delC	p.R85fs	frameshift deletion	2152	7	0.0032
48	4	20	33868573	33868573	20:33868573-33868573	G	-	EIF6	c.253delC	p.R85fs	frameshift deletion	2535	18	0.0071
48	6	20	33868573	33868573	20:33868573-33868573	G	-	EIF6	c.253delC	p.R85fs	frameshift deletion	2198	35	0.0157

48	A	20	33868621	33868621	20:33868621-33868621	C	T	EIF6	c.G205A	p.G69S	nonsynonymous SNV	607	83	0.1203
48	B	20	33868621	33868621	20:33868621-33868621	C	T	EIF6	c.G205A	p.G69S	nonsynonymous SNV	509	68	0.1179
48	Z	20	33868621	33868621	20:33868621-33868621	C	T	EIF6	c.G205A	p.G69S	nonsynonymous SNV	572	39	0.0638
48	1	20	33868621	33868621	20:33868621-33868621	C	T	EIF6	c.G205A	p.G69S	nonsynonymous SNV	1402	121	0.0794
48	2	20	33868621	33868621	20:33868621-33868621	C	T	EIF6	c.G205A	p.G69S	nonsynonymous SNV	1566	81	0.0492
48	4	20	33868621	33868621	20:33868621-33868621	C	T	EIF6	c.G205A	p.G69S	nonsynonymous SNV	1781	139	0.0724
48	6	20	33868621	33868621	20:33868621-33868621	C	T	EIF6	c.G205A	p.G69S	nonsynonymous SNV	1482	161	0.098
48	6	20	33868627	33868627	20:33868627-33868627	T	A	EIF6	c.A199T	p.R67W	nonsynonymous SNV	1523	3	0.002
48	6	20	33868633	33868633	20:33868633-33868633	C	T	EIF6	c.194-1G>A		splicing	1419	4	0.0028
48	4	20	33872034	33872034	20:33872034-33872034	G	-	EIF6	c.257delC	p.S86fs	frameshift deletion	2272	12	0.0053
48	6	20	33872034	33872034	20:33872034-33872034	G	-	EIF6	c.257delC	p.S86fs	frameshift deletion	1949	5	0.0026
48	2	20	33872253	33872253	20:33872253-33872253	A	T	EIF6	c.T38A	p.I13N	nonsynonymous SNV	2071	6	0.0029
48	6	20	33872260	33872262	20:33872260-33872262	0	-	EIF6	c.29_31del	p.10_11del	nonframeshift deletion	2008	6	0.003
48	6	17	1565303	1565303	17:1565303-1565303	T	C	PRPF8	c.A3919G	p.M1307V	nonsynonymous SNV	2141	4	0.0019
48	6	7	66459210	66459210	7:66459210-66459210	T	C	SBDS	c.A247G	p.I83V	nonsynonymous SNV	602	3	0.005
48	6	7	66460343	66460343	7:66460343-66460343	T	C	SBDS	c.A62G	p.K21R	nonsynonymous SNV	2106	3	0.0014
48	6	17	7577108	7577108	17:7577108-7577108	C	T	TP53	c.G830A	p.C277Y	nonsynonymous SNV	2285	4	0.0017
48	6	17	7577120	7577120	17:7577120-7577120	C	T	TP53	c.G818A	p.R273H	nonsynonymous SNV	2133	4	0.0019
48	2	17	7577511	7577511	17:7577511-7577511	A	C	TP53	c.T770G	p.L257R	nonsynonymous SNV	1444	5	0.0035
48	4	17	7577511	7577511	17:7577511-7577511	A	C	TP53	c.T770G	p.L257R	nonsynonymous SNV	1756	11	0.0062
48	6	17	7577511	7577511	17:7577511-7577511	A	C	TP53	c.T770G	p.L257R	nonsynonymous SNV	1535	21	0.0135
48	A	17	7577539	7577539	17:7577539-7577539	G	A	TP53	c.C742T	p.R248W	nonsynonymous SNV	748	32	0.041
48	B	17	7577539	7577539	17:7577539-7577539	G	A	TP53	c.C742T	p.R248W	nonsynonymous SNV	668	14	0.0205
48	Z	17	7577539	7577539	17:7577539-7577539	G	A	TP53	c.C742T	p.R248W	nonsynonymous SNV	817	12	0.0145
48	1	17	7577539	7577539	17:7577539-7577539	G	A	TP53	c.C742T	p.R248W	nonsynonymous SNV	1510	85	0.0533
48	2	17	7577539	7577539	17:7577539-7577539	G	A	TP53	c.C742T	p.R248W	nonsynonymous SNV	1754	53	0.0293
48	4	17	7577539	7577539	17:7577539-7577539	G	A	TP53	c.C742T	p.R248W	nonsynonymous SNV	2105	63	0.0291
48	6	17	7577539	7577539	17:7577539-7577539	G	A	TP53	c.C742T	p.R248W	nonsynonymous SNV	1878	57	0.0295
48	B	17	7577556	7577556	17:7577556-7577556	C	A	TP53	c.G725T	p.C242F	nonsynonymous SNV	654	28	0.0411
48	C	17	7577556	7577556	17:7577556-7577556	C	A	TP53	c.G725T	p.C242F	nonsynonymous SNV	46	20	0.303
48	2	17	7577556	7577556	17:7577556-7577556	C	A	TP53	c.G725T	p.C242F	nonsynonymous SNV	1807	3	0.0017
48	4	17	7577556	7577556	17:7577556-7577556	C	A	TP53	c.G725T	p.C242F	nonsynonymous SNV	2100	15	0.0071
48	6	17	7577556	7577556	17:7577556-7577556	C	A	TP53	c.G725T	p.C242F	nonsynonymous SNV	1775	129	0.0678
48	6	17	7578203	7578203	17:7578203-7578203	C	T	TP53	c.G646A	p.V216M	nonsynonymous SNV	1760	5	0.0028
48	4	17	7579358	7579358	17:7579358-7579358	C	A	TP53	c.G329T	p.R110L	nonsynonymous SNV	2558	3	0.0012
48	6	17	7579358	7579358	17:7579358-7579358	C	A	TP53	c.G329T	p.R110L	nonsynonymous SNV	2136	9	0.0042
49	A	20	33867743	33867743	20:33867743-33867743	A	C	EIF6	c.489+2T>G		splicing	622	20	0.0312
49	B	20	33867743	33867743	20:33867743-33867743	A	C	EIF6	c.489+2T>G		splicing	448	9	0.0197
49	C	20	33867743	33867743	20:33867743-33867743	A	C	EIF6	c.489+2T>G		splicing	610	5	0.0081
49	D	20	33867743	33867743	20:33867743-33867743	A	C	EIF6	c.489+2T>G		splicing	422	12	0.0276
49	E	20	33867743	33867743	20:33867743-33867743	A	C	EIF6	c.489+2T>G		splicing	531	14	0.0257
49	G	20	33867743	33867743	20:33867743-33867743	A	C	EIF6	c.489+2T>G		splicing	877	4	0.0045
49	H	20	33867743	33867743	20:33867743-33867743	A	C	EIF6	c.489+2T>G		splicing	697	9	0.0127
49	I	20	33867743	33867743	20:33867743-33867743	A	C	EIF6	c.489+2T>G		splicing	368	3	0.0081
49	Z	20	33867743	33867743	20:33867743-33867743	A	C	EIF6	c.489+2T>G		splicing	577	25	0.0415
49	G	20	33867758	33867760	20:33867758-33867760	0	-	EIF6	c.474_476del	p.158_159del	nonframeshift deletion	967	5	0.0051
49	E	20	33867758	33867760	20:33867758-33867760	0	-	EIF6	c.474_476del	p.158_159del	nonframeshift deletion	614	4	0.0065
49	A	20	33867758	33867760	20:33867758-33867760	0	-	EIF6	c.474_476del	p.158_159del	nonframeshift deletion	690	3	0.0043
49	Z	20	33867758	33867760	20:33867758-33867760	0	-	EIF6	c.474_476del	p.158_159del	nonframeshift deletion	674	7	0.0103
49	H	20	33867758	33867760	20:33867758-33867760	0	-	EIF6	c.474_476del	p.158_159del	nonframeshift deletion	805	4	0.0049
49	C	20	33867758	33867760	20:33867758-33867760	0	-	EIF6	c.474_476del	p.158_159del	nonframeshift deletion	674	3	0.0044
49	D	20	33867758	33867760	20:33867758-33867760	0	-	EIF6	c.474_476del	p.158_159del	nonframeshift deletion	497	3	0.006
49	A	20	33868461	33868461	20:33868461-33868461	T	A	EIF6	c.A365T	p.D122V	nonsynonymous SNV	693	7	0.01
49	B	20	33868461	33868461	20:33868461-33868461	T	A	EIF6	c.A365T	p.D122V	nonsynonymous SNV	491	3	0.0061
49	Z	20	33868461	33868461	20:33868461-33868461	T	A	EIF6	c.A365T	p.D122V	nonsynonymous SNV	698	6	0.0085
49	Z	20	33868491	33868491	20:33868491-33868491	T	A	EIF6	c.A335T	p.D112V	nonsynonymous SNV	855	4	0.0047
49	D	20	33871990	33871990	20:33871990-33871990	C	T	EIF6	c.G301A	p.A101T	nonsynonymous SNV	509	4	0.0078
49	E	20	33871990	33871990	20:33871990-33871990	C	T	EIF6	c.G301A	p.A101T	nonsynonymous SNV	574	9	0.0154
49	G	20	33871990	33871990	20:33871990-33871990	C	T	EIF6	c.G301A	p.A101T	nonsynonymous SNV	925	9	0.0096
49	H	20	33871990	33871990	20:33871990-33871990	C	T	EIF6	c.G301A	p.A101T	nonsynonymous SNV	797	12	0.0148
49	I	20	33871990	33871990	20:33871990-33871990	C	T	EIF6	c.G301A	p.A101T	nonsynonymous SNV	431	9	0.0205
49	B	17	1565301	1565301	17:1565301-1565301	C	T	PRPF8	c.G3921A	p.M1307I	nonsynonymous SNV	568	4	0.007
49	B	17	1565303	1565303	17:1565303-1565303	T	C	PRPF8	c.A3919G	p.M1307V	nonsynonymous SNV	569	4	0.007
49	C	17	1565303	1565303	17:1565303-1565303	T	C	PRPF8	c.A3919G	p.M1307V	nonsynonymous SNV	791	5	0.0063
49	D	17	1565303	1565303	17:1565303-1565303	T	C	PRPF8	c.A3919G	p.M1307V	nonsynonymous SNV	577	7	0.012
49	E	17	1565303	1565303	17:1565303-1565303	T	C	PRPF8	c.A3919G	p.M1307V	nonsynonymous SNV	684	8	0.0116
49	G	17	1565303	1565303	17:1565303-1565303	T	C	PRPF8	c.A3919G	p.M1307V	nonsynonymous SNV	1070	14	0.0129
49	H	17	1565303	1565303	17:1565303-1565303	T	C	PRPF8	c.A3919G	p.M1307V	nonsynonymous SNV	1025	5	0.0049
49	I	17	1565303	1565303	17:1565303-1565303	T	C	PRPF8	c.A3919G	p.M1307V	nonsynonymous SNV	471	3	0.0063
49	A	17	7577156	7577156	17:7577156-7577156	C	T	TP53	c.666-1G>A		splicing	639	9	0.0139
49	B	17	7577156	7577156	17:7577156-7577156	C	T	TP53	c.666-1G>A		splicing	429	4	0.0092
49	A	17	7578413	7578413	17:7578413-7578413	C	A	TP53	c.G517T	p.V173L	nonsynonymous SNV	930	3	0.0032
49	A	17	7579350	7579350	17:7579350-7579350	A	C	TP53	c.T337G	p.F113V	nonsynonymous SNV	843	8	0.0094
49	B	17	7579350	7579350	17:7579350-7579350	A	C	TP53	c.T337G	p.F113V	nonsynonymous SNV	575	6	0.0103
49	C	17	7579350	7579350	17:7579350-7579350	A	C	TP53	c.T337G	p.F113V	nonsynonymous SNV	846	8	0.0094
49	D	17	7579350	7579350	17:7579350-7579350	A	C	TP53	c.T337G	p.F113V	nonsynonymous SNV	642	5	0.0077
49	E	17	7579350	7579350	17:7579350-7579350	A	C	TP53	c.T337G	p.F113V	nonsynonymous SNV	689	7	0.0101
49	G	17	7579350	7579350	17:7579350-7579350	A	C	TP53	c.T337G	p.F113V	nonsynonymous SNV	1129	9	0.0079
49	H	17	7579350	7579350	17:7579350-7579350	A	C	TP53	c.T337G	p.F113V	nonsynonymous SNV	1079	12	0.011
49	I	17	7579350	7579350	17:7579350-7579350	A	C	TP53	c.T337G	p.F113V	nonsynonymous SNV	523	3	0.0057
49	Z	17	7579350	7579350	17:7579350-7579350	A	C	TP53	c.T337G	p.F113V	nonsynonymous SNV	821	14	0.0168
50	A	20	33867535	33867535	20:33867535-33867535	G	A	EIF6	c.C505T	p.R169X	stopgain	889	19	0.0209
50	B	20	33867535	33867535	20:33867535-33867535	G	A	EIF6	c.C505T	p.R169X	stopgain	515	14	0.0265
50	C	20	33867535	33867535	20:33867535-33867535	G	A	EIF6	c.C505T	p.R169X	stopgain	860	20	0.0227
50	A	20	33867793	33867793	20:33867793-33867793	T	-	EIF6	c.441delA	p.S147fs	frameshift deletion	1006	66	0.0616
50	B	20	33867793	33867793	20:33867793-33867793	T	-	EIF6	c.441delA	p.S147fs	frameshift deletion	552	40	0.0676
50	C	20	33867793	33867793	20:33867793-33867793	T	-	EIF6	c.441delA	p.S147fs	frameshift deletion	942	65	0.0645
50	A	20	33867866	33867869	20:33867866-33867869	0	-	EIF6	c.365_368del	p.T122fs	frameshift deletion	1081	12	0.011
50	B	20	33867866	33867869	20:33867866-33867869	0	-	EIF6	c.365_368del	p.T122fs	frameshift deletion	543	3	0.0055
50	C	20	33867866	33867869	20:33867866-33867869	0	-	EIF6	c.365_368del	p.T122fs	frameshift deletion	996	7	0.007
50	C	20	33868493	33868493	20:33868493-33868493	A	T	EIF6	c.T333A	p.N111K	nonsynonymous SNV	1041	4	0.0038
50	C	20	33868494	33868494	20:33868494-33868494	T	C	EIF6	c.A332G	p.N111S	nonsynonymous SNV	1048	3	0.0029
50	A	20	33868573	33868573	20:33868573-33868573	G	-	EIF6	c.253delC	p.R85fs	frameshift deletion	1037	3	0.0029
50	A	20	33871995	33871995	20:33871995-33871995	G	C	EIF6	c.C296G	p.S99W	nonsynonymous SNV	852	4	0.0047
50	B	20	33871995	33871995	20:33871995-33871995	G	C	EIF6	c.C296G	p.S99W	nonsynonymous SNV	495	3	0.006
50	C	20	33871995	33871995	20:33871995-33871995	G	C	EIF6	c.C296G	p.S99W	nonsynonymous SNV	796	3	0.0038
50	A	20	33872018	33872018	20:33872018-33872018	-	CG	EIF6	c.272_273insCG	p.R91fs	frameshift insertion	890	42	0.045
50	B	20	33872018	33872018	20:33872018-33872018	-	CG	EIF6	c.272_273insCG	p.R91fs	frameshift insertion	510	11	0.0211
50	C	20	33872018	33872018	20:33872018-33872018	-	CG	EIF6	c.272_273insCG	p.R91fs	frameshift insertion	845	37	0.042
50	A	20	33872257	33872257	20:33872257-33872257	C	T	EIF6	c.G34A	p.E12K	nonsynonymous SNV	968	5	0.0051
50	B	20	33872257	33872257	20:33872257-33872257	C	T	EIF6	c.G34A	p.E12K	nonsynonymous SNV	567	6	0.0105
50	C	20	33872257	33872257	20:33872257-33872257	C	T	EIF6	c.G34A	p.E12K	nonsynonymous SNV	896	9	0.0099
50	A	17	1565303	1565303	17:1565303-1565303	T	C	PRPF8	c.A3919G	p.M1307V	nonsynonymous SNV	980	15	0.0151
50	B	17	1565303	1565303	17:1565303-1565303	T	C	PRPF8	c.A3919G	p.M1307V	nonsynonymous SNV	497	16	0.0312
50	C	17	1565303	1565303	17:1565303-1565303	T	C	PRPF8	c.A3919G	p.M1307V	nonsynonymous SNV	904	34	0.0362
51	C	20	33867513	33867513	20:33867513-33867513	G	T	EIF6	c.C527A	p.A176D	nonsynonymous SNV	1008	5	0.0049
51	B	20	33867552	33867552	20:33867552-33867552	T	A	EIF6	c.490-2A>T		splicing	760	3	0.0039
51	B	20	33868509	33868509	20:33868509-33868509	T	C	EIF6	c.A317G	p.N106S	nonsynonymous SNV	1046	4	0.0038

51	C	20	33868509	33868509	20:33868509-33868509	T	C	EIF6	c.A317G	p.N106S	nonsynonymous SNV	1056	6	0.0056
51	A	20	33868540	33868540	20:33868540-33868540	G	A	EIF6	c.C286T	p.R96W	nonsynonymous SNV	807	4	0.0049
51	C	20	33868540	33868540	20:33868540-33868540	G	A	EIF6	c.C286T	p.R96W	nonsynonymous SNV	1053	3	0.0028
51	A	20	33872236	33872236	20:33872236-33872236	G	A	EIF6	c.C55T	p.L19F	nonsynonymous SNV	933	10	0.0106
51	B	20	33872236	33872236	20:33872236-33872236	G	A	EIF6	c.C55T	p.L19F	nonsynonymous SNV	983	9	0.0091
51	C	20	33872236	33872236	20:33872236-33872236	G	A	EIF6	c.C55T	p.L19F	nonsynonymous SNV	950	3	0.0031
51	B	20	33872239	33872239	20:33872239-33872239	T	-	EIF6	c.52delA	p.K18fs	frameshift deletion	997	3	0.003
51	C	20	33872239	33872239	20:33872239-33872239	T	-	EIF6	c.52delA	p.K18fs	frameshift deletion	942	4	0.0042
51	B	7	66459201	66459201	7:66459201-66459201	G	T	SBDS	c.C256A	p.Q86K	nonsynonymous SNV	200	11	0.0521
51	C	7	66459201	66459201	7:66459201-66459201	G	T	SBDS	c.C256A	p.Q86K	nonsynonymous SNV	200	39	0.1632
51	A	17	7577580	7577580	17:7577580-7577580	T	C	TP53	c.A701G	p.Y234C	nonsynonymous SNV	658	3	0.0045
52	H	20	33867536	33867536	20:33867536-33867536	G	T	EIF6	c.C504A	p.N168K	nonsynonymous SNV	1317	4	0.003
52	H	20	33867782	33867782	20:33867782-33867782	T	-	EIF6	c.452delA	p.Q151fs	frameshift deletion	1458	6	0.0041
52	G	20	33872052	33872052	20:33872052-33872052	-	C	EIF6	c.238dupG	p.A80fs	frameshift insertion	922	3	0.0032
53	B	20	33868495	33868495	20:33868495-33868495	T	G	EIF6	c.A331C	p.N111H	nonsynonymous SNV	622	8	0.0127
53	A	20	33868540	33868540	20:33868540-33868540	G	A	EIF6	c.C286T	p.R96W	nonsynonymous SNV	1133	5	0.0044
53	B	20	33868540	33868540	20:33868540-33868540	G	A	EIF6	c.C286T	p.R96W	nonsynonymous SNV	668	4	0.006
53	C	20	33868540	33868540	20:33868540-33868540	G	A	EIF6	c.C286T	p.R96W	nonsynonymous SNV	982	6	0.0061
53	D	20	33868540	33868540	20:33868540-33868540	G	A	EIF6	c.C286T	p.R96W	nonsynonymous SNV	1106	6	0.0054
53	E	20	33868540	33868540	20:33868540-33868540	G	A	EIF6	c.C286T	p.R96W	nonsynonymous SNV	1319	11	0.0083
53	D	20	33871990	33871990	20:33871990-33871990	C	T	EIF6	c.G301A	p.A101T	nonsynonymous SNV	856	3	0.0035
53	B	20	33872005	33872005	20:33872005-33872005	C	T	EIF6	c.G286A	p.A96T	nonsynonymous SNV	639	4	0.0062
53	E	20	33872035	33872035	20:33872035-33872035	A	C	EIF6	c.T256G	p.S86A	nonsynonymous SNV	1145	3	0.0026
53	D	20	33872184	33872184	20:33872184-33872184	C	G	EIF6	c.G107C	p.R36T	nonsynonymous SNV	1017	3	0.0029
53	A	20	33872197	33872197	20:33872197-33872197	C	A	EIF6	c.G94T	p.E32X	stopgain	1133	22	0.019
53	B	20	33872197	33872197	20:33872197-33872197	C	A	EIF6	c.G94T	p.E32X	stopgain	747	15	0.0197
53	C	20	33872197	33872197	20:33872197-33872197	C	A	EIF6	c.G94T	p.E32X	stopgain	961	12	0.0123
53	D	20	33872197	33872197	20:33872197-33872197	C	A	EIF6	c.G94T	p.E32X	stopgain	1036	7	0.0067
53	E	20	33872197	33872197	20:33872197-33872197	C	A	EIF6	c.G94T	p.E32X	stopgain	1254	4	0.0032
53	E	17	7579389	7579389	17:7579389-7579389	G	A	TP53	c.C298T	p.Q100X	stopgain	1307	3	0.0023
56	B	20	33872211	33872211	20:33872211-33872211	G	A	EIF6	c.C80T	p.A27V	nonsynonymous SNV	1043	7	0.0067
57	G	20	33868509	33868509	20:33868509-33868509	T	C	EIF6	c.A317G	p.N106S	nonsynonymous SNV	1082	4	0.0037
57	C	17	7577539	7577539	17:7577539-7577539	G	A	TP53	c.C742T	p.R248W	nonsynonymous SNV	1010	3	0.003
57	D	17	7577539	7577539	17:7577539-7577539	G	A	TP53	c.C742T	p.R248W	nonsynonymous SNV	1159	7	0.006
57	E	17	7577539	7577539	17:7577539-7577539	G	A	TP53	c.C742T	p.R248W	nonsynonymous SNV	886	5	0.0056
57	G	17	7577539	7577539	17:7577539-7577539	G	A	TP53	c.C742T	p.R248W	nonsynonymous SNV	914	5	0.0054
57	H	17	7577539	7577539	17:7577539-7577539	G	A	TP53	c.C742T	p.R248W	nonsynonymous SNV	1001	6	0.006
57	B	17	7578507	7578512	17:7578507-7578512	0	-	TP53	c.418_423del	p.140_141del	nonframeshift deletion	901	3	0.0033
58	D	20	33867495	33867500	20:33867495-33867500	0	-	EIF6	c.540_545del	p.180_182del	nonframeshift deletion	831	5	0.006
58	D	20	33871990	33871990	20:33871990-33871990	C	T	EIF6	c.G301A	p.A101T	nonsynonymous SNV	649	5	0.0076
58	C	17	7577082	7577082	17:7577082-7577082	C	T	TP53	c.G856A	p.E286K	nonsynonymous SNV	1047	4	0.0038
58	D	17	7577082	7577082	17:7577082-7577082	C	T	TP53	c.G856A	p.E286K	nonsynonymous SNV	874	4	0.0046
62	A	20	33867790	33867790	20:33867790-33867790	-	G	EIF6	c.443_444insC	p.I148fs	frameshift insertion	976	3	0.0031
62	A	20	33867791	33867791	20:33867791-33867791	A	T	EIF6	c.T443A	p.I148N	nonsynonymous SNV	987	3	0.003
62	C	20	33868509	33868509	20:33868509-33868509	T	C	EIF6	c.A317G	p.N106S	nonsynonymous SNV	922	3	0.0032
62	C	20	33868611	33868611	20:33868611-33868611	A	C	EIF6	c.T215G	p.V72G	nonsynonymous SNV	706	3	0.0042
63	D	20	31022441	31022441	20:31022441-31022441	-	G	ASXL1	c.1927dupG	p.G642fs	frameshift insertion	525	6	0.0113
63	E	20	31022441	31022441	20:31022441-31022441	-	G	ASXL1	c.1927dupG	p.G642fs	frameshift insertion	571	7	0.0121
63	A	20	33868494	33868494	20:33868494-33868494	T	C	EIF6	c.A332G	p.N111S	nonsynonymous SNV	794	3	0.0038
63	D	17	7577120	7577120	17:7577120-7577120	C	T	TP53	c.G818A	p.R273H	nonsynonymous SNV	1013	3	0.003
64	A	20	33867001	33867005	20:33867001-33867005	0	-	EIF6	c.676_680del	p.T226fs	frameshift deletion	902	24	0.0259
64	B	20	33867001	33867005	20:33867001-33867005	0	-	EIF6	c.676_680del	p.T226fs	frameshift deletion	679	19	0.0272
64	A	20	33867866	33867869	20:33867866-33867869	0	-	EIF6	c.365_368del	p.T122fs	frameshift deletion	947	12	0.0125
64	B	20	33867866	33867869	20:33867866-33867869	0	-	EIF6	c.365_368del	p.T122fs	frameshift deletion	680	10	0.0145
64	A	20	33867912	33867912	20:33867912-33867912	C	T	EIF6	c.G322A	p.E108K	nonsynonymous SNV	491	193	0.2822
64	B	20	33867912	33867912	20:33867912-33867912	C	T	EIF6	c.G322A	p.E108K	nonsynonymous SNV	365	100	0.2151
64	A	20	33868509	33868509	20:33868509-33868509	T	C	EIF6	c.A317G	p.N106S	nonsynonymous SNV	957	7	0.0073
64	B	20	33868509	33868509	20:33868509-33868509	T	C	EIF6	c.A317G	p.N106S	nonsynonymous SNV	747	5	0.0066
65	A	20	33867888	33867888	20:33867888-33867888	C	T	EIF6	c.G346A	p.V116M	nonsynonymous SNV	1101	3	0.0027
65	A	20	33867923	33867923	20:33867923-33867923	T	C	EIF6	c.313-2A>G		splicing	824	3	0.0036
65	A	17	7577539	7577539	17:7577539-7577539	G	A	TP53	c.C742T	p.R248W	nonsynonymous SNV	950	32	0.0326
67	D	20	33867773	33867773	20:33867773-33867773	A	C	EIF6	c.T461G	p.L154R	nonsynonymous SNV	967	4	0.0041
67	C	20	33868540	33868540	20:33868540-33868540	G	A	EIF6	c.C286T	p.R96W	nonsynonymous SNV	1231	4	0.0032
67	D	20	33868540	33868540	20:33868540-33868540	G	A	EIF6	c.C286T	p.R96W	nonsynonymous SNV	1134	7	0.0061
67	E	20	33868540	33868540	20:33868540-33868540	G	A	EIF6	c.C286T	p.R96W	nonsynonymous SNV	1359	6	0.0044
67	G	20	33868540	33868540	20:33868540-33868540	G	A	EIF6	c.C286T	p.R96W	nonsynonymous SNV	968	3	0.0031
68	A	20	33868467	33868467	20:33868467-33868467	T	A	EIF6	c.A359T	p.D120V	nonsynonymous SNV	1103	18	0.0161
68	A	20	33868488	33868488	20:33868488-33868488	T	C	EIF6	c.A338G	p.Y113C	nonsynonymous SNV	1254	4	0.0032
68	B	20	33868488	33868488	20:33868488-33868488	T	C	EIF6	c.A338G	p.Y113C	nonsynonymous SNV	758	5	0.0066
68	A	20	33868509	33868509	20:33868509-33868509	T	C	EIF6	c.A317G	p.N106S	nonsynonymous SNV	1292	5	0.0039
68	B	20	33868509	33868509	20:33868509-33868509	T	C	EIF6	c.A317G	p.N106S	nonsynonymous SNV	805	3	0.0037
68	B	20	33868540	33868540	20:33868540-33868540	G	A	EIF6	c.C286T	p.R96W	nonsynonymous SNV	835	3	0.0036
68	A	20	33872222	33872222	20:33872222-33872222	-	T	EIF6	c.68dupA	p.Y23_C24delinsX	stopgain	1213	26	0.021
68	B	20	33872222	33872222	20:33872222-33872222	-	T	EIF6	c.68dupA	p.Y23_C24delinsX	stopgain	744	14	0.0185
68	B	17	7577555	7577555	17:7577555-7577555	G	C	TP53	c.C726G	p.C242W	nonsynonymous SNV	805	3	0.0037
68	A	17	7577568	7577568	17:7577568-7577568	C	A	TP53	c.G713T	p.C238F	nonsynonymous SNV	1062	4	0.0038
68	A	17	7578271	7578271	17:7578271-7578271	T	C	TP53	c.A578G	p.H193R	nonsynonymous SNV	1193	6	0.005
68	B	17	7578271	7578271	17:7578271-7578271	T	C	TP53	c.A578G	p.H193R	nonsynonymous SNV	743	3	0.004
68	B	17	7578413	7578413	17:7578413-7578413	C	T	TP53	c.G517A	p.V173M	nonsynonymous SNV	801	3	0.0037
69	A	5	148904673	148904673	5:148904673-148904673	C	T	CSNK1A1	c.G292A	p.E98K	nonsynonymous SNV	1231	5	0.004
69	A	20	33867749	33867749	20:33867749-33867749	A	T	EIF6	c.T485A	p.L162H	nonsynonymous SNV	992	3	0.003
69	A	20	33868540	33868540	20:33868540-33868540	G	A	EIF6	c.C286T	p.R96W	nonsynonymous SNV	1418	27	0.0187
69	A	20	33868582	33868582	20:33868582-33868582	G	A	EIF6	c.C244T	p.Q82X	stopgain	1415	3	0.0021
69	A	20	33872058	33872058	20:33872058-33872058	G	-	EIF6	c.114delC	p.F38fs	frameshift deletion	1094	5	0.0045
69	A	17	7577556	7577556	17:7577556-7577556	C	T	TP53	c.G725A	p.C242Y	nonsynonymous SNV	1336	5	0.0037
69	A	17	7578394	7578394	17:7578394-7578394	T	C	TP53	c.A536G	p.H179R	nonsynonymous SNV	1407	5	0.0035
69	A	17	7578403	7578403	17:7578403-7578403	C	T	TP53	c.G527A	p.C176Y	nonsynonymous SNV	1425	11	0.0077
69	A	17	7578406	7578406	17:7578406-7578406	C	T	TP53	c.G524A	p.R175H	nonsynonymous SNV	1436	3	0.0021
69	A	17	7578479	7578479	17:7578479-7578479	G	A	TP53	c.C451T	p.P151S	nonsynonymous SNV	1385	7	0.005
70	D	20	33867742	33867744	20:33867742-33867744	0	-	EIF6			splicing	755	3	0.004
70	B	20	33868461	33868461	20:33868461-33868461	T	A	EIF6	c.A365T	p.D122V	nonsynonymous SNV	837	3	0.0036
70	C	20	33868461	33868461	20:33868461-33868461	T	A	EIF6	c.A365T	p.D122V	nonsynonymous SNV	710	4	0.0056
70	C	17	7577094	7577094	17:7577094-7577094	G	A	TP53	c.C844T	p.R282W	nonsynonymous SNV	861	8	0.0092
70	D	17	7577108	7577108	17:7577108-7577108	C	T	TP53	c.G830A	p.C277Y	nonsynonymous SNV	1133	7	0.0061
70	C	17	7577520	7577520	17:7577520-7577520	A	C	TP53	c.T761G	p.I254S	nonsynonymous SNV	675	5	0.0074
70	D	17	7577538	7577538	17:7577538-7577538	C	T	TP53	c.G743A	p.R248Q	nonsynonymous SNV	937	3	0.0032
70	A	17	7578191	7578191	17:7578191-7578191	A	T	TP53	c.T658A	p.Y220N	nonsynonymous SNV	769	34	0.0423
70	B	17	7578191	7578191	17:7578191-7578191	A	T	TP53	c.T658A	p.Y220N	nonsynonymous SNV	733	39	0.0505
70	C	17	7578191	7578191	17:7578191-7578191	A	T	TP53	c.T658A	p.Y220N	nonsynonymous SNV	614	39	0.0597
70	D	17	7578191	7578191	17:7578191-7578191	A	T	TP53	c.T658A	p.Y220N	nonsynonymous SNV	752	41	0.0517
70	D	17	7578518	7578518	17:7578518-7578518	C	G	TP53	c.G412C	p.A138P	nonsynonymous SNV	1051	17	0.0159
70	D	17	7578535	7578535	17:7578535-7578535	T	C	TP53	c.A395G	p.K132R	nonsynonymous SNV	932	3	0.0032
71	A	20	33867480	33867480	20:33867480-33867480	C	T	EIF6	c.G560A	p.C187Y	nonsynonymous SNV	1043	10	0.0095
71	A	20	33867537	33867537	20:33867537-33867537	T	A	EIF6	c.A503T	p.N168I	nonsynonymous SNV	824	34	0.0396
71	A	20	33868621	33868621	20:33868621-33868621	C	T	EIF6	c.G205A	p.G69S	nonsynonymous SNV	818	5	0.0061
71	A	17	7577090	7577090	17:7577090-7577090	C	G	TP53	c.G848C	p.R283P	nonsynonymous SNV	703	107	0.1321
72	B	20	33867492	33867492	20:33867492-33867492	C	T	EIF6	c.G548A	p.W183X	stopgain	1052	5	0.0047
72	C	20	33872205	33872205	20:33872205-33872205	C	A	EIF6	c.G86T	p.G29V	nonsynonymous SNV	932	3	0.0032

72	A	20	33872289	33872289	20:33872289-33872289	A	G	EIF6	c.T2C	p.M1T	nonsynonymous SNV	605	124	0.1701
72	B	20	33872289	33872289	20:33872289-33872289	A	G	EIF6	c.T2C	p.M1T	nonsynonymous SNV	665	79	0.1062
72	C	20	33872289	33872289	20:33872289-33872289	A	G	EIF6	c.T2C	p.M1T	nonsynonymous SNV	727	61	0.0774
72	D	20	33872289	33872289	20:33872289-33872289	A	G	EIF6	c.T2C	p.M1T	nonsynonymous SNV	558	42	0.07
72	A	4	106155778	106155778	4:106155778-106155778	G	T	TET2	c.G679T	p.E227X	stopgain	969	30	0.03
72	B	4	106155778	106155778	4:106155778-106155778	G	T	TET2	c.G679T	p.E227X	stopgain	1153	15	0.0128
72	C	4	106155778	106155778	4:106155778-106155778	G	T	TET2	c.G679T	p.E227X	stopgain	946	17	0.0177
72	D	4	106155778	106155778	4:106155778-106155778	G	T	TET2	c.G679T	p.E227X	stopgain	890	5	0.0056
73	C	20	33867922	33867922	20:33867922-33867922	C	G	EIF6	c.313-1G>C		splicing	1004	9	0.0089
73	C	20	33868509	33868509	20:33868509-33868509	T	C	EIF6	c.A317G	p.N106S	nonsynonymous SNV	1651	3	0.0018
73	C	20	33868594	33868594	20:33868594-33868594	C	A	EIF6	c.G232T	p.D78Y	nonsynonymous SNV	1535	3	0.002
73	D	20	33872065	33872065	20:33872065-33872065	C	A	EIF6	c.G226T	p.V76L	nonsynonymous SNV	931	5	0.0053
73	C	20	33872257	33872257	20:33872257-33872257	-	A	EIF6	c.33dupT	p.E12_I13delinsX	stopgain	1387	5	0.0036
74	D	20	33868519	33868519	20:33868519-33868519	C	G	EIF6	c.G307C	p.A103P	nonsynonymous SNV	1241	14	0.0112
75	A	20	33867453	33867453	20:33867453-33867453	A	G	EIF6	c.T587C	p.L196P	nonsynonymous SNV	1369	38	0.027
75	C	20	33867761	33867761	20:33867761-33867761	A	C	EIF6	c.T473G	p.L158R	nonsynonymous SNV	1240	5	0.004
75	A	20	33868495	33868495	20:33868495-33868495	T	C	EIF6	c.A331G	p.N111D	nonsynonymous SNV	1355	11	0.0081
75	A	20	33868509	33868509	20:33868509-33868509	T	C	EIF6	c.A317G	p.N106S	nonsynonymous SNV	1342	19	0.014
75	B	20	33868509	33868509	20:33868509-33868509	T	C	EIF6	c.A317G	p.N106S	nonsynonymous SNV	929	3	0.0032
75	C	20	33868509	33868509	20:33868509-33868509	T	C	EIF6	c.A317G	p.N106S	nonsynonymous SNV	1561	13	0.0083
75	1	20	33868509	33868509	20:33868509-33868509	T	C	EIF6	c.A317G	p.N106S	nonsynonymous SNV	1392	4	0.0029
75	A	20	33868551	33868551	20:33868551-33868551	A	T	EIF6	c.T275A	p.V92E	nonsynonymous SNV	1279	3	0.0023
75	B	20	33868575	33868575	20:33868575-33868575	-	T	EIF6	c.250dupA	p.I84fs	frameshift insertion	917	7	0.0076
75	A	20	33868608	33868608	20:33868608-33868608	G	C	EIF6	c.C218G	p.P73R	nonsynonymous SNV	1134	17	0.0148
75	A	20	33868632	33868632	20:33868632-33868632	C	A	EIF6	c.G194T	p.G65V	nonsynonymous SNV	893	5	0.0056
75	A	17	1565303	1565303	17:1565303-1565303	T	C	PRPF8	c.A3919G	p.M1307V	nonsynonymous SNV	1095	128	0.1047
75	B	17	1565303	1565303	17:1565303-1565303	T	C	PRPF8	c.A3919G	p.M1307V	nonsynonymous SNV	581	223	0.2774
75	C	17	1565303	1565303	17:1565303-1565303	T	C	PRPF8	c.A3919G	p.M1307V	nonsynonymous SNV	951	459	0.3255
75	1	17	1565303	1565303	17:1565303-1565303	T	C	PRPF8	c.A3919G	p.M1307V	nonsynonymous SNV	780	471	0.3765
75	B	17	7577557	7577557	17:7577557-7577557	A	T	TP53	c.T724A	p.C242S	nonsynonymous SNV	848	3	0.0035
75	A	17	7578235	7578235	17:7578235-7578235	T	C	TP53	c.A614G	p.Y205C	nonsynonymous SNV	1201	9	0.0074
76	D	20	33867477	33867477	20:33867477-33867477	C	T	EIF6	c.G563A	p.G188D	nonsynonymous SNV	1335	3	0.0022
76	E	20	33868519	33868519	20:33868519-33868519	C	G	EIF6	c.G307C	p.A103P	nonsynonymous SNV	1302	7	0.0053
76	G	20	33868519	33868519	20:33868519-33868519	C	G	EIF6	c.G307C	p.A103P	nonsynonymous SNV	855	7	0.0081
76	H	20	33868519	33868519	20:33868519-33868519	C	G	EIF6	c.G307C	p.A103P	nonsynonymous SNV	980	132	0.1187
78	A	20	33867534	33867534	20:33867534-33867534	C	G	EIF6	c.G506C	p.R169P	nonsynonymous SNV	853	38	0.0426
78	B	20	33867534	33867534	20:33867534-33867534	C	G	EIF6	c.G506C	p.R169P	nonsynonymous SNV	989	12	0.012
78	C	20	33867534	33867534	20:33867534-33867534	C	G	EIF6	c.G506C	p.R169P	nonsynonymous SNV	1019	23	0.0221
78	D	20	33867534	33867534	20:33867534-33867534	C	G	EIF6	c.G506C	p.R169P	nonsynonymous SNV	995	18	0.0178
78	E	20	33867534	33867534	20:33867534-33867534	C	G	EIF6	c.G506C	p.R169P	nonsynonymous SNV	930	22	0.0231
78	G	20	33867534	33867534	20:33867534-33867534	C	G	EIF6	c.G506C	p.R169P	nonsynonymous SNV	777	22	0.0275
78	H	20	33867534	33867534	20:33867534-33867534	C	G	EIF6	c.G506C	p.R169P	nonsynonymous SNV	871	23	0.0257
78	B	20	33868608	33868608	20:33868608-33868608	G	A	EIF6	c.C218T	p.P73L	nonsynonymous SNV	1042	6	0.0057
78	E	20	33868608	33868608	20:33868608-33868608	G	A	EIF6	c.C218T	p.P73L	nonsynonymous SNV	981	5	0.0051
78	G	20	33868608	33868608	20:33868608-33868608	G	A	EIF6	c.C218T	p.P73L	nonsynonymous SNV	810	5	0.0061
78	H	20	33868608	33868608	20:33868608-33868608	G	A	EIF6	c.C218T	p.P73L	nonsynonymous SNV	891	4	0.0045
78	B	17	7577094	7577094	17:7577094-7577094	G	C	TP53	c.C844G	p.R282G	nonsynonymous SNV	1392	3	0.0022
78	C	17	7577094	7577094	17:7577094-7577094	G	C	TP53	c.C844G	p.R282G	nonsynonymous SNV	1343	3	0.0022
78	D	17	7577094	7577094	17:7577094-7577094	G	C	TP53	c.C844G	p.R282G	nonsynonymous SNV	1263	3	0.0024
78	G	17	7577094	7577094	17:7577094-7577094	G	C	TP53	c.C844G	p.R282G	nonsynonymous SNV	983	9	0.0091
78	H	17	7577095	7577095	17:7577095-7577095	G	T	TP53	c.C843A	p.D281E	nonsynonymous SNV	1082	3	0.0028
78	G	17	7577120	7577120	17:7577120-7577120	C	T	TP53	c.G818A	p.R273H	nonsynonymous SNV	946	3	0.0032
78	H	17	7577120	7577120	17:7577120-7577120	C	T	TP53	c.G818A	p.R273H	nonsynonymous SNV	1067	4	0.0037
78	A	17	7577538	7577538	17:7577538-7577538	C	T	TP53	c.G743A	p.R248Q	nonsynonymous SNV	983	29	0.0287
78	B	17	7577538	7577538	17:7577538-7577538	C	T	TP53	c.G743A	p.R248Q	nonsynonymous SNV	1150	18	0.0154
78	C	17	7577538	7577538	17:7577538-7577538	C	T	TP53	c.G743A	p.R248Q	nonsynonymous SNV	1124	26	0.0226
78	D	17	7577538	7577538	17:7577538-7577538	C	T	TP53	c.G743A	p.R248Q	nonsynonymous SNV	1068	33	0.03
78	E	17	7577538	7577538	17:7577538-7577538	C	T	TP53	c.G743A	p.R248Q	nonsynonymous SNV	1153	23	0.0196
78	G	17	7577538	7577538	17:7577538-7577538	C	T	TP53	c.G743A	p.R248Q	nonsynonymous SNV	887	13	0.0144
78	H	17	7577538	7577538	17:7577538-7577538	C	T	TP53	c.G743A	p.R248Q	nonsynonymous SNV	1013	17	0.0165
78	G	17	7578406	7578406	17:7578406-7578406	C	T	TP53	c.G524A	p.R175H	nonsynonymous SNV	1085	3	0.0028
79	A	20	33868540	33868540	20:33868540-33868540	G	A	EIF6	c.C286T	p.R96W	nonsynonymous SNV	1197	5	0.0042
80	A	20	33868540	33868540	20:33868540-33868540	G	A	EIF6	c.C286T	p.R96W	nonsynonymous SNV	1070	6	0.0056
80	A	20	33868582	33868582	20:33868582-33868582	G	A	EIF6	c.C244T	p.Q82X	stopgain	1115	7	0.0062
80	A	20	33872032	33872032	20:33872032-33872032	G	A	EIF6	c.C259T	p.P87S	nonsynonymous SNV	797	127	0.1374
80	A	21	36259172	36259172	21:36259172-36259172	G	C	RUNX1	c.C319G	p.R107G	nonsynonymous SNV	1083	10	0.0091
80	A	17	7577538	7577538	17:7577538-7577538	C	T	TP53	c.G743A	p.R248Q	nonsynonymous SNV	1059	3	0.0028
82	A	20	33868494	33868494	20:33868494-33868494	T	C	EIF6	c.A332G	p.N111S	nonsynonymous SNV	1089	16	0.0145
82	A	20	33868540	33868540	20:33868540-33868540	G	A	EIF6	c.C286T	p.R96W	nonsynonymous SNV	1111	3	0.0027
82	A	17	1563731	1563731	17:1563731-1563731	A	G	PRPF8	c.T4780C	p.C1594R	nonsynonymous SNV	754	3	0.004
82	A	17	7577094	7577094	17:7577094-7577094	G	A	TP53	c.C844T	p.R282W	nonsynonymous SNV	1088	8	0.0073
86	B	5	148904672	148904672	5:148904672-148904672	T	C	CSNK1A1	c.A293G	p.E98G	nonsynonymous SNV	775	5	0.0064
86	B	5	148904673	148904673	5:148904673-148904673	C	T	CSNK1A1	c.G292A	p.E98K	nonsynonymous SNV	703	78	0.0999
86	A	20	33867749	33867749	20:33867749-33867749	A	G	EIF6	c.T485C	p.L162P	nonsynonymous SNV	709	44	0.0584
86	B	20	33867749	33867749	20:33867749-33867749	A	G	EIF6	c.T485C	p.L162P	nonsynonymous SNV	637	54	0.0781
86	B	20	33867858	33867858	20:33867858-33867858	G	A	EIF6	c.C376T	p.Q126X	stopgain	877	3	0.0034
86	B	20	33872187	33872188	20:33872187-33872188	0	-	EIF6	c.103_104del	p.Y35fs	frameshift deletion	858	7	0.0081
86	A	20	33872217	33872217	20:33872217-33872217	A	C	EIF6	c.T74G	p.L25R	nonsynonymous SNV	987	3	0.003
86	B	20	33872250	33872250	20:33872250-33872250	C	T	EIF6	c.G41A	p.G14D	nonsynonymous SNV	908	4	0.0044
86	A	17	1565295	1565295	17:1565295-1565295	A	T	PRPF8	c.T3927A	p.S1309R	nonsynonymous SNV	921	8	0.0086
86	B	17	1565295	1565295	17:1565295-1565295	A	T	PRPF8	c.T3927A	p.S1309R	nonsynonymous SNV	834	3	0.0036
86	A	17	7577532	7577532	17:7577532-7577532	G	A	TP53	c.C749T	p.P250L	nonsynonymous SNV	883	3	0.0034
86	B	17	7577556	7577556	17:7577556-7577556	C	G	TP53	c.G725C	p.C242S	nonsynonymous SNV	826	5	0.006
86	A	17	7578406	7578406	17:7578406-7578406	C	T	TP53	c.G524A	p.R175H	nonsynonymous SNV	1035	3	0.0029
86	B	17	7578406	7578406	17:7578406-7578406	C	T	TP53	c.G524A	p.R175H	nonsynonymous SNV	1013	5	0.0049
87	A	2	25469958	25469958	2:25469958-25469958	G	A	DNMT3A	c.C1084T	p.Q362X	stopgain	1256	3	0.0024
87	A	20	33867513	33867513	20:33867513-33867513	G	T	EIF6	c.C527A	p.A176D	nonsynonymous SNV	1256	9	0.0071
87	A	20	33867818	33867818	20:33867818-33867818	C	A	EIF6	c.G416T	p.G139V	nonsynonymous SNV	1298	3	0.0023
87	A	20	33872297	33872297	20:33872297-33872297	T	G	EIF6	EIF6(NM_181466:exon2:UTR5,		splicing	791	4	0.005
87	A	17	7577094	7577094	17:7577094-7577094	G	A	TP53	c.C844T	p.R282W	nonsynonymous SNV	1364	27	0.0194
87	A	17	7577557	7577557	17:7577557-7577557	A	-	TP53	c.724delT	p.C242fs	frameshift deletion	1167	9	0.0077
87	A	17	7577568	7577568	17:7577568-7577568	C	T	TP53	c.G713A	p.C238Y	nonsynonymous SNV	1100	3	0.0027
87	A	17	7578205	7578205	17:7578205-7578205	C	A	TP53	c.G644T	p.S215I	nonsynonymous SNV	1124	21	0.0183
87	A	17	7578406	7578406	17:7578406-7578406	C	T	TP53	c.G524A	p.R175H	nonsynonymous SNV	1351	3	0.0022
87	A	17	7578503	7578503	17:7578503-7578503	C	T	TP53	c.G427A	p.V143M	nonsynonymous SNV	1284	10	0.0077
87	A	17	7579358	7579358	17:7579358-7579358	C	G	TP53	c.G329C	p.R110P	nonsynonymous SNV	499	715	0.589
88	A	20	33867749	33867749	20:33867749-33867749	A	T	EIF6	c.T485A	p.L162H	nonsynonymous SNV	1538	5	0.0032
88	A	20	33867888	33867888	20:33867888-33867888	C	n	EIF6	c.G346A	p.V116M	nonsynonymous SNV	1700	4	0.0023
88	C	20	33867888	33867888	20:33867888-33867888	C	T	EIF6	c.G346A	p.V116M	nonsynonymous SNV	1029	3	0.0029
88	A	20	33868540	33868540	20:33868540-33868540	G	A	EIF6	c.C286T	p.R96W	nonsynonymous SNV	2064	3	0.0015
88	A	17	7577559	7577559	17:7577559-7577559	G	A	TP53	c.C722T	p.S241F	nonsynonymous SNV	1607	3	0.0019
88	B	17	7577581	7577581	17:7577581-7577581	A	G	TP53	c.T700C	p.Y234H	nonsynonymous SNV	683	3	0.0044
88	A	17	7578397	7578397	17:7578397-7578397	T	G	TP53	c.A533C	p.H178P	nonsynonymous SNV	2047	6	0.0029
89	E	7	140453155	140453155	7:140453155-140453155	C	T	BRAF	c.G1780A	p.D594N	nonsynonymous SNV	572	238	0.2938
89	G	7	140453155	140453155	7:140453155-140453155	C	T	BRAF	c.G1780A	p.D594N	nonsynonymous SNV	923	235	0.2029
89	B	20	33868540	33868540	20:33868540-33868540	G	A	EIF6	c.C286T	p.R96W	nonsynonymous SNV	975	5	0.0051
89	C	20	33868540	33868540	20:33868540-33868540	G	A	EIF6	c.C286T	p.R96W	nonsynonymous SNV	1328	3	0.0023
89	A	20	33872224	33872224	20:33872224-33872224	A	G	EIF6	c.T67C	p.Y23H	nonsynonymous SNV	1188	3	0.0025

89	B	20	33872224	33872224	20:33872224-33872224	A	G	EIF6	c.T67C	p.Y23H	nonsynonymous SNV	893	7	0.0078
89	C	20	33872224	33872224	20:33872224-33872224	A	G	EIF6	c.T67C	p.Y23H	nonsynonymous SNV	1391	6	0.0043
89	E	12	25380279	25380279	12:25380279-25380279	C	A	KRAS	c.G179T	p.G60V	nonsynonymous SNV	985	8	0.0081
89	G	12	25380279	25380279	12:25380279-25380279	C	A	KRAS	c.G179T	p.G60V	nonsynonymous SNV	1625	22	0.0134
89	E	1	115258747	115258747	1:115258747-115258747	C	T	NRAS	c.G35A	p.G12D	nonsynonymous SNV	839	144	0.1465
89	G	1	115258747	115258747	1:115258747-115258747	C	T	NRAS	c.G35A	p.G12D	nonsynonymous SNV	1068	267	0.2
89	G	7	66460343	66460343	7:66460343-66460343	T	C	SBDS	c.A62G	p.K21R	nonsynonymous SNV	1453	3	0.0021
89	D	18	42531924	42531926	18:42531924-42531926	0	-	SETBP1	c.2619_2621del	p.873_874del	nonframeshift deletion	519	50	0.0879
89	E	18	42531924	42531926	18:42531924-42531926	0	-	SETBP1	c.2619_2621del	p.873_874del	nonframeshift deletion	698	292	0.2949
89	G	18	42531924	42531926	18:42531924-42531926	0	-	SETBP1	c.2619_2621del	p.873_874del	nonframeshift deletion	1046	268	0.204
89	B	17	7578406	7578406	17:7578406-7578406	C	T	TP53	c.G524A	p.R175H	nonsynonymous SNV	794	5	0.0063
89	C	17	7578406	7578406	17:7578406-7578406	C	T	TP53	c.G524A	p.R175H	nonsynonymous SNV	1285	3	0.0023
90	B	20	33867858	33867858	20:33867858-33867858	G	A	EIF6	c.C376T	p.Q126X	stopgain	973	5	0.0051
92	A	20	33867511	33867511	20:33867511-33867511	C	T	EIF6	c.G529A	p.G177R	nonsynonymous SNV	1703	14	0.0082
92	1	20	33867511	33867511	20:33867511-33867511	C	T	EIF6	c.G529A	p.G177R	nonsynonymous SNV	1544	4	0.0026
92	A	20	33867534	33867534	20:33867534-33867534	C	G	EIF6	c.G506C	p.R169P	nonsynonymous SNV	1481	3	0.002
92	A	20	33867742	33867744	20:33867742-33867744	0	-	EIF6			splicing	1207	10	0.0082
92	1	20	33867742	33867744	20:33867742-33867744	0	-	EIF6			splicing	1029	23	0.0219
92	A	20	33868540	33868540	20:33868540-33868540	G	A	EIF6	c.C286T	p.R96W	nonsynonymous SNV	1635	3	0.0018
92	1	20	33871979	33871979	20:33871979-33871979	-	CACA	EIF6	c.311_312insTGTG	p.W104fs	frameshift insertion	1107	21	0.0186
92	A	7	148504764	148504764	7:148504764-148504764	-	GCGGG	EZH2	c.2229_2230insCC(	p.I744fs	frameshift insertion	1103	14	0.0125
92	1	7	148504764	148504764	7:148504764-148504764	-	GCGGG	EZH2	c.2229_2230insCC(	p.I744fs	frameshift insertion	1014	14	0.0136
92	A	17	1565303	1565303	17:1565303-1565303	T	C	PRPF8	c.A3919G	p.M1307V	nonsynonymous SNV	1608	5	0.0031
92	A	21	36231791	36231791	21:36231791-36231791	T	C	RUNX1	c.A593G	p.D198G	nonsynonymous SNV	1328	68	0.0487
92	1	21	36231791	36231791	21:36231791-36231791	T	C	RUNX1	c.A593G	p.D198G	nonsynonymous SNV	1127	92	0.0755
92	1	17	7577524	7577524	17:7577524-7577524	T	G	TP53	c.A757C	p.T253P	nonsynonymous SNV	1236	38	0.0298
92	A	17	7577539	7577539	17:7577539-7577539	G	A	TP53	c.C742T	p.R248W	nonsynonymous SNV	1491	4	0.0027
92	A	17	7578271	7578271	17:7578271-7578271	T	C	TP53	c.A578G	p.H193R	nonsynonymous SNV	1565	3	0.0019
92	1	17	7578271	7578271	17:7578271-7578271	T	C	TP53	c.A578G	p.H193R	nonsynonymous SNV	1343	4	0.003
93	B	20	33867468	33867468	20:33867468-33867468	G	A	EIF6	c.C572T	p.T191I	nonsynonymous SNV	1231	3	0.0024
93	A	20	33867498	33867498	20:33867498-33867498	T	A	EIF6	c.A542T	p.N181I	nonsynonymous SNV	1084	5	0.0046
93	B	20	33867498	33867498	20:33867498-33867498	T	A	EIF6	c.A542T	p.N181I	nonsynonymous SNV	1180	4	0.0034
93	A	20	33867755	33867755	20:33867755-33867755	A	C	EIF6	c.T479G	p.V160G	nonsynonymous SNV	821	4	0.0048
93	A	20	33867812	33867812	20:33867812-33867812	A	G	EIF6	c.T422C	p.L141P	nonsynonymous SNV	1108	8	0.0072
93	B	20	33867812	33867812	20:33867812-33867812	A	G	EIF6	c.T422C	p.L141P	nonsynonymous SNV	1354	3	0.0022
93	B	20	33867828	33867830	20:33867828-33867830	0	-	EIF6	c.404_406del	p.135_136del	nonframeshift deletion	1336	3	0.0022
93	A	20	33867922	33867922	20:33867922-33867922	C	T	EIF6	c.313-1G>A		splicing	650	21	0.0313
93	B	20	33867922	33867922	20:33867922-33867922	C	T	EIF6	c.313-1G>A		splicing	868	17	0.0192
93	A	20	33872184	33872184	20:33872184-33872184	C	A	EIF6	c.G107T	p.R36M	nonsynonymous SNV	1086	4	0.0037
93	B	20	33872184	33872184	20:33872184-33872184	C	A	EIF6	c.G107T	p.R36M	nonsynonymous SNV	961	4	0.0041
93	A	20	33872222	33872222	20:33872222-33872222	-	T	EIF6	c.68dupA	p.Y23_C24delinsX	stopgain	1127	3	0.0027
93	B	17	7574034	7574034	17:7574034-7574034	C	G	TP53	c.877-1G>C		splicing	1097	5	0.0045
93	B	17	7574035	7574035	17:7574035-7574035	T	G	TP53	c.877-2A>C		splicing	1087	5	0.0046
93	A	17	7577081	7577081	17:7577081-7577081	T	A	TP53	c.A857T	p.E286V	nonsynonymous SNV	1207	6	0.0049
93	B	17	7577081	7577081	17:7577081-7577081	T	A	TP53	c.A857T	p.E286V	nonsynonymous SNV	1386	5	0.0036
93	A	17	7577094	7577094	17:7577094-7577094	G	A	TP53	c.C844T	p.R282W	nonsynonymous SNV	1194	11	0.0091
93	B	17	7577094	7577094	17:7577094-7577094	G	A	TP53	c.C844T	p.R282W	nonsynonymous SNV	1378	15	0.0108
93	A	17	7577095	7577095	17:7577095-7577095	G	C	TP53	c.C843G	p.D281E	nonsynonymous SNV	1192	4	0.0033
93	B	17	7577095	7577095	17:7577095-7577095	G	C	TP53	c.C843G	p.D281E	nonsynonymous SNV	1393	3	0.0021
93	A	17	7577100	7577100	17:7577100-7577100	T	C	TP53	c.A838G	p.R280G	nonsynonymous SNV	1186	5	0.0042
93	B	17	7577100	7577100	17:7577100-7577100	T	C	TP53	c.A838G	p.R280G	nonsynonymous SNV	1388	9	0.0064
93	A	17	7577120	7577120	17:7577120-7577120	-	GCA	TP53	c.817_818insTGC	p.R273delinsLR	nonframeshift insertion	1136	3	0.0026
93	A	17	7577120	7577120	17:7577120-7577120	-	GCA	TP53	c.817_818insTGC	p.R273delinsLR	nonframeshift insertion	1136	31	0.0265
93	B	17	7577120	7577120	17:7577120-7577120	-	GCA	TP53	c.817_818insTGC	p.R273delinsLR	nonframeshift insertion	1287	48	0.0359
93	A	17	7577141	7577141	17:7577141-7577141	C	T	TP53	c.G797A	p.G266E	nonsynonymous SNV	968	3	0.0031
93	A	17	7577498	7577498	17:7577498-7577498	C	T	TP53	c.665+1G>A		splicing	846	4	0.0047
93	B	17	7577498	7577498	17:7577498-7577498	C	T	TP53	c.665+1G>A		splicing	915	7	0.0076
93	A	17	7577511	7577511	17:7577511-7577511	A	G	TP53	c.T770C	p.L257P	nonsynonymous SNV	954	4	0.0042
93	B	17	7577511	7577511	17:7577511-7577511	A	G	TP53	c.T770C	p.L257P	nonsynonymous SNV	1021	5	0.0049
93	A	17	7577556	7577556	17:7577556-7577556	C	G	TP53	c.G725C	p.C242S	nonsynonymous SNV	1096	5	0.0045
93	A	17	7577609	7577609	17:7577609-7577609	C	A	TP53	c.556-1G>T		splicing	749	4	0.0053
93	A	17	7578190	7578190	17:7578190-7578190	T	C	TP53	c.A659G	p.Y220C	nonsynonymous SNV	867	10	0.0114
93	B	17	7578190	7578190	17:7578190-7578190	T	C	TP53	c.A659G	p.Y220C	nonsynonymous SNV	1020	5	0.0049
93	B	17	7578437	7578437	17:7578437-7578437	G	A	TP53	c.C493T	p.Q165X	stopgain	1425	3	0.0021
93	A	17	7578442	7578442	17:7578442-7578442	T	C	TP53	c.A488G	p.Y163C	nonsynonymous SNV	1263	16	0.0125
93	B	17	7578442	7578442	17:7578442-7578442	T	C	TP53	c.A488G	p.Y163C	nonsynonymous SNV	1401	17	0.012
93	A	17	7578476	7578476	17:7578476-7578476	G	T	TP53	c.C454A	p.P152T	nonsynonymous SNV	1176	6	0.0051
93	B	17	7578476	7578476	17:7578476-7578476	G	T	TP53	c.C454A	p.P152T	nonsynonymous SNV	1258	13	0.0102
93	A	17	7579345	7579347	17:7579345-7579347	0	-	TP53	c.340_342del	p.114_114del	nonframeshift deletion	1149	4	0.0035
93	B	17	7579345	7579347	17:7579345-7579347	0	-	TP53	c.340_342del	p.114_114del	nonframeshift deletion	1374	3	0.0022
94	A	20	33867844	33867844	20:33867844-33867844	T	-	EIF6	c.390delA	p.G130fs	frameshift deletion	978	10	0.0101
95	A	20	33867511	33867511	20:33867511-33867511	C	T	EIF6	c.G529A	p.G177R	nonsynonymous SNV	880	23	0.0255
95	B	20	33867511	33867511	20:33867511-33867511	C	T	EIF6	c.G529A	p.G177R	nonsynonymous SNV	707	17	0.0235
95	A	20	33867758	33867760	20:33867758-33867760	0	-	EIF6	c.474_476del	p.158_159del	nonframeshift deletion	746	3	0.004
95	B	20	33867758	33867760	20:33867758-33867760	0	-	EIF6	c.474_476del	p.158_159del	nonframeshift deletion	580	4	0.0068
95	A	20	33867888	33867888	20:33867888-33867888	C	T	EIF6	c.G346A	p.V116M	nonsynonymous SNV	775	4	0.0051
95	A	20	33868509	33868509	20:33868509-33868509	T	C	EIF6	c.A317G	p.N106S	nonsynonymous SNV	853	3	0.0035
95	B	20	33868509	33868509	20:33868509-33868509	T	C	EIF6	c.A317G	p.N106S	nonsynonymous SNV	747	7	0.0093
95	A	12	12037507	12037507	12:12037507-12037507	T	A	ETV6	c.T1138A	p.W380R	nonsynonymous SNV	713	16	0.0219
95	B	17	7577120	7577120	17:7577120-7577120	C	T	TP53	c.G818A	p.R273H	nonsynonymous SNV	855	3	0.0035
95	B	17	7578402	7578402	17:7578402-7578402	G	C	TP53	c.C528G	p.C176W	nonsynonymous SNV	903	5	0.0055
95	A	17	7578406	7578406	17:7578406-7578406	C	T	TP53	c.G524A	p.R175H	nonsynonymous SNV	1121	6	0.0053
95	B	17	7578406	7578406	17:7578406-7578406	C	T	TP53	c.G524A	p.R175H	nonsynonymous SNV	892	17	0.0187
96	A	18	42531907	42531907	18:42531907-42531907	G	A	SETBP1	c.G2602A	p.D868N	nonsynonymous SNV	713	178	0.1998
96	B	18	42531907	42531907	18:42531907-42531907	G	A	SETBP1	c.G2602A	p.D868N	nonsynonymous SNV	598	278	0.3174
98	1	5	148904673	148904673	5:148904673-148904673	C	T	CSNK1A1	c.G292A	p.E98K	nonsynonymous SNV	1390	49	0.0341
98	3	5	148904673	148904673	5:148904673-148904673	C	T	CSNK1A1	c.G292A	p.E98K	nonsynonymous SNV	1429	79	0.0524
98	A	5	148904673	148904673	5:148904673-148904673	C	T	CSNK1A1	c.G292A	p.E98K	nonsynonymous SNV	907	44	0.0463
98	B	5	148904673	148904673	5:148904673-148904673	C	T	CSNK1A1	c.G292A	p.E98K	nonsynonymous SNV	917	70	0.0709
98	C	5	148904673	148904673	5:148904673-148904673	C	T	CSNK1A1	c.G292A	p.E98K	nonsynonymous SNV	1000	18	0.0177
98	D	5	148904673	148904673	5:148904673-148904673	C	T	CSNK1A1	c.G292A	p.E98K	nonsynonymous SNV	990	11	0.011
98	1	20	33867367	33867367	20:33867367-33867367	A	T	EIF6	c.671+2T>A		splicing	1122	3	0.0027
98	3	20	33867367	33867367	20:33867367-33867367	A	T	EIF6	c.671+2T>A		splicing	1215	3	0.0025
98	A	20	33867367	33867367	20:33867367-33867367	A	T	EIF6	c.671+2T>A		splicing	615	5	0.0081
98	3	20	33867744	33867744	20:33867744-33867744	C	T	EIF6	c.489+1G>A		splicing	1252	9	0.0071
98	A	20	33867744	33867744	20:33867744-33867744	C	T	EIF6	c.489+1G>A		splicing	717	8	0.011
98	A	20	33867759	33867759	20:33867759-33867759	G	-	EIF6	c.475delC	p.Q159fs	frameshift deletion	795	7	0.0087
98	3	20	33867819	33867819	20:33867819-33867819	C	T	EIF6	c.G415A	p.G139R	nonsynonymous SNV	1926	9	0.0047
98	A	20	33867819	33867819	20:33867819-33867819	C	T	EIF6	c.G415A	p.G139R	nonsynonymous SNV	965	3	0.0031
98	1	20	33868509	33868509	20:33868509-33868509	T	C	EIF6	c.A317G	p.N106S	nonsynonymous SNV	1682	14	0.0083
98	3	20	33868509	33868509	20:33868509-33868509	T	C	EIF6	c.A317G	p.N106S	nonsynonymous SNV	1940	13	0.0067
98	A	20	33868509	33868509	20:33868509-33868509	T	C	EIF6	c.A317G	p.N106S	nonsynonymous SNV	1016	5	0.0049
98	B	20	33868509	33868509	20:33868509-33868509	T	C	EIF6	c.A317G	p.N106S	nonsynonymous SNV	1194	5	0.0042
98	D	20	33868509	33868509	20:33868509-33868509	T	C	EIF6	c.A317G	p.N106S	nonsynonymous SNV	1075	3	0.0028
98	3	20	33868521	33868521	20:33868521-33868521	G	C	EIF6	c.C305G	p.S102X	stopgain	1947	6	0.0031
98	A	20	33868521	33868521	20:33868521-33868521	G	C	EIF6	c.C305G	p.S102X	stopgain	991	7	0.007
98	3	20	33868531	33868531	20:33868531-33868531	C	G	EIF6	c.G295C	p.E99Q	nonsynonymous SNV	1923	14	0.0072
98	A	20	33868531	33868531	20:33868531-33868531	C	G	EIF6	c.G295C	p.E99Q	nonsynonymous SNV	982	7	0.0071

98	B	20	33868531	33868531	20:33868531-33868531	C	G	EIF6	c.G295C	p.E99Q	nonsynonymous SNV	1151	4	0.0035
98	A	20	33868546	33868550	20:33868546-33868550	0	-	EIF6	c.276_280del	p.V92fs	frameshift deletion	1003	4	0.004
98	A	20	33868552	33868553	20:33868552-33868553	0	-	EIF6	c.273_274del	p.T91fs	frameshift deletion	1000	3	0.003
98	1	20	33868608	33868608	20:33868608-33868608	G	C	EIF6	c.C218G	p.P73R	nonsynonymous SNV	1416	5	0.0035
98	3	20	33868608	33868608	20:33868608-33868608	G	C	EIF6	c.C218G	p.P73R	nonsynonymous SNV	1663	13	0.0078
98	A	20	33868608	33868608	20:33868608-33868608	G	C	EIF6	c.C218G	p.P73R	nonsynonymous SNV	863	9	0.0103
98	B	20	33868608	33868608	20:33868608-33868608	G	C	EIF6	c.C218G	p.P73R	nonsynonymous SNV	1023	3	0.0029
98	C	20	33868608	33868608	20:33868608-33868608	G	C	EIF6	c.C218G	p.P73R	nonsynonymous SNV	998	7	0.007
98	D	20	33868608	33868608	20:33868608-33868608	G	C	EIF6	c.C218G	p.P73R	nonsynonymous SNV	902	4	0.0044
98	A	20	33872232	33872232	20:33872232-33872232	G	A	EIF6	c.C59T	p.T20I	nonsynonymous SNV	933	4	0.0043
98	1	20	33872269	33872269	20:33872269-33872269	C	A	EIF6	c.G22T	p.E8X	stopgain	1370	3	0.0022
98	3	20	33872269	33872269	20:33872269-33872269	C	A	EIF6	c.G22T	p.E8X	stopgain	1662	8	0.0048
98	A	20	33872269	33872269	20:33872269-33872269	C	A	EIF6	c.G22T	p.E8X	stopgain	867	5	0.0057
98	B	20	33872269	33872269	20:33872269-33872269	C	A	EIF6	c.G22T	p.E8X	stopgain	950	3	0.0031
98	3	17	1565295	1565295	17:1565295-1565295	A	C	PRPF8	c.T3927G	p.S1309R	nonsynonymous SNV	1711	7	0.0041
98	3	17	1565295	1565295	17:1565295-1565295	A	C	PRPF8	c.T3927G	p.S1309R	nonsynonymous SNV	1711	4	0.0023
98	A	7	66459281	66459281	7:66459281-66459281	T	C	SBDS	c.A176G	p.N59S	nonsynonymous SNV	422	3	0.0071
98	B	17	7578290	7578290	17:7578290-7578290	C	T	TP53	c.443-1G>A		splicing	1003	3	0.003
98	1	17	7578404	7578404	17:7578404-7578404	A	T	TP53	c.T526A	p.C176S	nonsynonymous SNV	1724	3	0.0017
98	B	17	7578404	7578404	17:7578404-7578404	A	T	TP53	c.T526A	p.C176S	nonsynonymous SNV	1212	3	0.0025
98	1	17	7578496	7578496	17:7578496-7578496	A	G	TP53	c.T434C	p.L145P	nonsynonymous SNV	1635	18	0.0109
98	3	17	7578496	7578496	17:7578496-7578496	A	G	TP53	c.T434C	p.L145P	nonsynonymous SNV	1798	5	0.0028
98	B	17	7578496	7578496	17:7578496-7578496	A	G	TP53	c.T434C	p.L145P	nonsynonymous SNV	1114	5	0.0045
98	C	17	7578496	7578496	17:7578496-7578496	A	G	TP53	c.T434C	p.L145P	nonsynonymous SNV	1187	5	0.0042
98	D	17	7578496	7578496	17:7578496-7578496	A	G	TP53	c.T434C	p.L145P	nonsynonymous SNV	1053	7	0.0066
99	A	4	106197221	106197221	4:106197221-106197221	C	T	TET2	c.C5554T	p.Q1852X	stopgain	2091	11	0.0052
99	A	17	7577100	7577100	17:7577100-7577100	T	C	TP53	c.A838G	p.R280G	nonsynonymous SNV	1729	3	0.0017
99	B	17	7577100	7577100	17:7577100-7577100	T	C	TP53	c.A838G	p.R280G	nonsynonymous SNV	1334	4	0.003
99	A	17	7577533	7577535	17:7577533-7577535	0	-	TP53	c.746_748del	p.249_250del	nonframeshift deletion	1410	3	0.0021
99	A	17	7577539	7577539	17:7577539-7577539	G	A	TP53	c.C742T	p.R248W	nonsynonymous SNV	1480	3	0.002
99	A	17	7578394	7578394	17:7578394-7578394	T	C	TP53	c.A536G	p.H179R	nonsynonymous SNV	1772	3	0.0017
99	B	17	7578394	7578394	17:7578394-7578394	T	C	TP53	c.A536G	p.H179R	nonsynonymous SNV	1314	6	0.0045
99	B	17	7578413	7578413	17:7578413-7578413	C	T	TP53	c.G517A	p.V173M	nonsynonymous SNV	1341	3	0.0022
101	B	20	33867478	33867478	20:33867478-33867478	C	G	EIF6	c.G562C	p.G188R	nonsynonymous SNV	1275	8	0.0062
101	B	20	33867498	33867498	20:33867498-33867498	T	G	EIF6	c.A542C	p.N181T	nonsynonymous SNV	1259	4	0.0032
101	B	20	33867505	33867505	20:33867505-33867505	C	T	EIF6	c.G535A	p.V179M	nonsynonymous SNV	1249	4	0.0032
101	A	20	33867510	33867510	20:33867510-33867510	C	A	EIF6	c.G530T	p.G177V	nonsynonymous SNV	1014	40	0.038
101	B	20	33867510	33867510	20:33867510-33867510	C	A	EIF6	c.G530T	p.G177V	nonsynonymous SNV	1137	83	0.068
101	A	20	33867535	33867535	20:33867535-33867535	G	A	EIF6	c.C505T	p.R169X	stopgain	900	25	0.027
101	B	20	33867535	33867535	20:33867535-33867535	G	A	EIF6	c.C505T	p.R169X	stopgain	995	26	0.0255
101	B	20	33867813	33867816	20:33867813-33867816	0	-	EIF6	c.418_421del	p.G140fs	frameshift deletion	1295	4	0.0031
101	B	20	33867815	33867815	20:33867815-33867815	C	T	EIF6	c.G419A	p.G140E	nonsynonymous SNV	1296	3	0.0023
101	B	20	33868492	33868492	20:33868492-33868492	C	T	EIF6	c.G334A	p.D112N	nonsynonymous SNV	1223	9	0.0073
101	A	20	33871978	33871978	20:33871978-33871978	C	T	EIF6	c.312+1G>A		splicing	732	43	0.0555
101	B	20	33871978	33871978	20:33871978-33871978	C	T	EIF6	c.312+1G>A		splicing	846	54	0.06
101	B	20	33872250	33872250	20:33872250-33872250	C	T	EIF6	c.G41A	p.G14D	nonsynonymous SNV	1240	3	0.0024
101	A	20	33872283	33872283	20:33872283-33872283	A	T	EIF6	c.T8A	p.V3D	nonsynonymous SNV	812	4	0.0049
101	B	20	33872283	33872283	20:33872283-33872283	A	T	EIF6	c.T8A	p.V3D	nonsynonymous SNV	1011	5	0.0049
101	A	17	1565303	1565303	17:1565303-1565303	T	C	PRPF8	c.A3919G	p.M1307V	nonsynonymous SNV	877	6	0.0068
101	B	17	1565303	1565303	17:1565303-1565303	T	C	PRPF8	c.A3919G	p.M1307V	nonsynonymous SNV	1076	8	0.0074
101	A	17	7577143	7577145	17:7577143-7577145	0	-	TP53	c.793_795del	p.265_265del	nonframeshift deletion	765	6	0.0078
101	B	17	7579463	7579463	17:7579463-7579463	G	C	TP53	c.C224G	p.P75R	nonsynonymous SNV	1318	3	0.0023
102	A	20	33867845	33867845	20:33867845-33867845	C	T	EIF6	c.G389A	p.G130E	nonsynonymous SNV	1933	28	0.0143
102	A	20	33868509	33868509	20:33868509-33868509	T	C	EIF6	c.A317G	p.N106S	nonsynonymous SNV	1845	6	0.0032
102	A	1	115258747	115258747	1:115258747-115258747	C	T	NRAS	c.G35A	p.G12D	nonsynonymous SNV	1042	585	0.3596
102	Z	1	115258747	115258747	1:115258747-115258747	C	T	NRAS	c.G35A	p.G12D	nonsynonymous SNV	356	258	0.4202
102	A	17	1565303	1565303	17:1565303-1565303	T	C	PRPF8	c.A3919G	p.M1307V	nonsynonymous SNV	1765	4	0.0023
102	A	17	7578532	7578532	17:7578532-7578532	A	T	TP53	c.T398A	p.M133K	nonsynonymous SNV	336	1085	0.7635
102	Z	17	7578532	7578532	17:7578532-7578532	A	T	TP53	c.T398A	p.M133K	nonsynonymous SNV	99	523	0.8408
106	B	20	33867001	33867001	20:33867001-33867001	C	A	EIF6	c.G680T	p.X227L	stoploss	1015	24	0.0231
106	A	20	33867001	33867001	20:33867001-33867001	C	A	EIF6	c.G680T	p.X227L	stoploss	845	19	0.022
106	A	20	33867410	33867410	20:33867410-33867410	A	-	EIF6	c.630delT	p.P210fs	frameshift deletion	835	8	0.0095
106	B	20	33867410	33867410	20:33867410-33867410	A	-	EIF6	c.630delT	p.P210fs	frameshift deletion	968	4	0.0041
106	A	20	33868628	33868628	20:33868628-33868628	G	T	EIF6	c.C198A	p.N66K	nonsynonymous SNV	607	5	0.0082
106	B	20	33868628	33868628	20:33868628-33868628	G	T	EIF6	c.C198A	p.N66K	nonsynonymous SNV	775	3	0.0039
106	B	20	33872002	33872002	20:33872002-33872002	C	G	EIF6	c.G289C	p.A97P	nonsynonymous SNV	890	8	0.0089
106	A	20	33872067	33872067	20:33872067-33872067	G	C	EIF6	c.C224G	p.T75R	nonsynonymous SNV	651	3	0.0046
106	A	20	33872182	33872182	20:33872182-33872182	A	C	EIF6	c.T109G	p.C37G	nonsynonymous SNV	751	3	0.004
106	B	20	33872182	33872182	20:33872182-33872182	A	C	EIF6	c.T109G	p.C37G	nonsynonymous SNV	847	5	0.0059
106	A	20	33872224	33872224	20:33872224-33872224	A	-	EIF6	c.67delT	p.Y23fs	frameshift deletion	767	10	0.0129
106	B	20	33872224	33872224	20:33872224-33872224	A	-	EIF6	c.67delT	p.Y23fs	frameshift deletion	940	13	0.0136
106	B	20	33872250	33872250	20:33872250-33872250	C	T	EIF6	c.G41A	p.G14D	nonsynonymous SNV	952	4	0.0042
106	B	20	33872266	33872266	20:33872266-33872266	T	C	EIF6	c.A25G	p.N9D	nonsynonymous SNV	902	4	0.0044
106	A	20	33872277	33872277	20:33872277-33872277	G	T	EIF6	c.C14A	p.A5D	nonsynonymous SNV	674	6	0.0088
106	B	20	33872277	33872277	20:33872277-33872277	G	T	EIF6	c.C14A	p.A5D	nonsynonymous SNV	835	4	0.0048
106	A	17	7577082	7577082	17:7577082-7577082	C	T	TP53	c.G856A	p.E286K	nonsynonymous SNV	864	3	0.0035
106	B	17	7577082	7577082	17:7577082-7577082	C	T	TP53	c.G856A	p.E286K	nonsynonymous SNV	1159	6	0.0052
106	A	17	7577094	7577094	17:7577094-7577094	G	A	TP53	c.C844T	p.R282W	nonsynonymous SNV	858	7	0.0081
106	B	17	7577094	7577094	17:7577094-7577094	G	A	TP53	c.C844T	p.R282W	nonsynonymous SNV	1143	10	0.0087
106	A	17	7578542	7578542	17:7578542-7578542	G	A	TP53	c.C388T	p.L130F	nonsynonymous SNV	709	5	0.007
106	B	17	7578542	7578542	17:7578542-7578542	G	A	TP53	c.C388T	p.L130F	nonsynonymous SNV	850	12	0.0139
107	A	20	33867468	33867468	20:33867468-33867468	G	A	EIF6	c.C572T	p.T191I	nonsynonymous SNV	1387	131	0.0863
107	B	20	33867468	33867468	20:33867468-33867468	G	A	EIF6	c.C572T	p.T191I	nonsynonymous SNV	983	5	0.0051
107	B	20	33867546	33867546	20:33867546-33867546	C	T	EIF6	c.G494A	p.G165E	nonsynonymous SNV	661	3	0.0045
107	A	2	209113113	209113113	2:209113113-209113113	G	A	IDH1	c.C394T	p.R132C	nonsynonymous SNV	651	206	0.2404
107	B	2	209113113	209113113	2:209113113-209113113	G	A	IDH1	c.C394T	p.R132C	nonsynonymous SNV	384	213	0.3568
107	A	17	7578455	7578455	17:7578455-7578455	C	G	TP53	c.G475C	p.A159P	nonsynonymous SNV	1432	4	0.0028
107	B	17	7578455	7578455	17:7578455-7578455	C	G	TP53	c.G475C	p.A159P	nonsynonymous SNV	948	4	0.0042
108	A	20	33867535	33867535	20:33867535-33867535	G	A	EIF6	c.C505T	p.R169X	stopgain	1360	6	0.0044
108	A	17	1565295	1565295	17:1565295-1565295	A	T	PRPF8	c.T3927A	p.S1309R	nonsynonymous SNV	1376	4	0.0029
108	B	4	106197300	106197300	4:106197300-106197300	G	A	TET2	c.G5633A	p.R1878H	nonsynonymous SNV	782	6	0.0076
108	A	17	7577581	7577581	17:7577581-7577581	A	T	TP53	c.T700A	p.Y234N	nonsynonymous SNV	507	482	0.4874
108	A	17	7578479	7578479	17:7578479-7578479	G	T	TP53	c.C451A	p.P151T	nonsynonymous SNV	1476	4	0.0027
110	A	20	33867486	33867486	20:33867486-33867486	G	T	EIF6	c.C554A	p.A185D	nonsynonymous SNV	1375	8	0.0058
110	A	20	33868512	33868512	20:33868512-33868512	C	T	EIF6	c.G314A	p.G105D	nonsynonymous SNV	1352	3	0.0022
110	A	20	33868629	33868629	20:33868629-33868629	T	C	EIF6	c.A197G	p.N66S	nonsynonymous SNV	970	3	0.0031
110	A	17	1565301	1565301	17:1565301-1565301	C	G	PRPF8	c.G3921C	p.M1307I	nonsynonymous SNV	1170	40	0.0331
110	A	7	66453398	66453398	7:66453398-66453398	T	G	SBDS	c.A713C	p.N238T	nonsynonymous SNV	1024	3	0.0029
110	A	17	7577144	7577144	17:7577144-7577144	A	G	TP53	c.T794C	p.L265P	nonsynonymous SNV	1063	3	0.0028
110	A	17	7577551	7577551	17:7577551-7577551	C	A	TP53	c.G730T	p.G244C	nonsynonymous SNV	1111	131	0.1055
110	A	17	7577557	7577557	17:7577557-7577557	A	T	TP53	c.T724A	p.C242S	nonsynonymous SNV	1203	16	0.0131
110	A	17	7578397	7578397	17:7578397-7578397	T	G	TP53	c.A533C	p.H178P	nonsynonymous SNV	1271	4	0.0031
