# Supplementary material for: Distinct genetic pathways define pre-malignant versus compensatory clonal hematopoiesis in Shwachman-Diamond syndrome
Source: Nat Commun. 2021 Feb 26;12:1334. doi: 10.1038/s41467-021-21588-4 (PMC7910481; doi:10.1038/s41467-021-21588-4)
Supplement: Supplementary file 2 — Descriptions of Additional Supplementary Files [file 41467_2021_21588_MOESM2_ESM.pdf]

Descriptions of Additional Supplementary Files

Supplementary software 1

Description: Rosetta scripts
