# Supplementary material for: Distinct genetic pathways define pre-malignant versus compensatory clonal hematopoiesis in Shwachman-Diamond syndrome
Source: Nat Commun. 2021 Feb 26;12:1334. doi: 10.1038/s41467-021-21588-4 (PMC7910481; doi:10.1038/s41467-021-21588-4)
Supplement: Supplementary file 4 — Reporting Summary [file 41467_2021_21588_MOESM4_ESM.pdf]

nature research

Corresponding author(s): R. Coleman Lindsley

Last updated by author(s): Dec 22, 2020

Reporting Summary

Nature Research wishes to improve the reproducibility of the work that we publish. This form provides structure for consistency and transparency in reporting. For further information on Nature Research policies, see our Editorial Policies and the Editorial Policy Checklist.

Statistics

For all statistical analyses, confirm that the following items are present in the figure legend, table legend, main text, or Methods section.

n/a	Confirmed	
☐	☒	The exact sample size (*n*) for each experimental group/condition, given as a discrete number and unit of measurement
☐	☒	A statement on whether measurements were taken from distinct samples or whether the same sample was measured repeatedly
☐	☒	The statistical test(s) used AND whether they are one- or two-sided<br>*Only common tests should be described solely by name; describe more complex techniques in the Methods section.*
☐	☒	A description of all covariates tested
☒	☐	A description of any assumptions or corrections, such as tests of normality and adjustment for multiple comparisons
☐	☒	A full description of the statistical parameters including central tendency (e.g. means) or other basic estimates (e.g. regression coefficient) AND variation (e.g. standard deviation) or associated estimates of uncertainty (e.g. confidence intervals)
☐	☒	For null hypothesis testing, the test statistic (e.g. *F*, *t*, *r*) with confidence intervals, effect sizes, degrees of freedom and *P* value noted<br>*Give P values as exact values whenever suitable.*
☒	☐	For Bayesian analysis, information on the choice of priors and Markov chain Monte Carlo settings
☒	☐	For hierarchical and complex designs, identification of the appropriate level for tests and full reporting of outcomes
☒	☐	Estimates of effect sizes (e.g. Cohen's *d*, Pearson's *r*), indicating how they were calculated

Our web collection on statistics for biologists contains articles on many of the points above.

Software and code

Policy information about availability of computer code

Data collection	Tapestri v2.2 (MissionBio), FacsDIVA v8.0.3, FlowJo v10.6.2 , Graphpad Prism v8.4.3, Rosetta 2019.09.post.dev+3.master.7d95c8a 7d95c8a9c33961c6618181f85a2c5ca5c2f6c552, PyMOL v.1.8.4.0.
Data analysis	Custom Rosetta scripts that was used for in silico modeling of human EIF6 are included in the in supplementary files associated with this article as Supplementary Software 1.

For manuscripts utilizing custom algorithms or software that are central to the research but not yet described in published literature, software must be made available to editors and reviewers. We strongly encourage code deposition in a community repository (e.g. GitHub). See the Nature Research guidelines for submitting code & software for further information.

Data

Policy information about availability of data

All manuscripts must include a data availability statement. This statement should provide the following information, where applicable:

- Accession codes, unique identifiers, or web links for publicly available datasets
- A list of figures that have associated raw data
- A description of any restrictions on data availability

Sequence data has been deposited at the European Genome-phenome Archive (EGA), which is hosted by the EBI and the CRG, under accession numbers EGAS00001004879, EGAS00001004880, EGAS00001004881 and can be accessed on https://ega-archive.org The remaining data are available within the Article, Supplementary Information, or available from the authors upon request. Source data are provided with this paper including figures 1D,E; 2D,E,F,G,H,I,K ; 3A,B,C; 4B; 5A,B,C,F,G; 6B,C; Supplementary Figures: 1; 3A,B,D; 4A,B.

Field-specific reporting

Please select the one below that is the best fit for your research. If you are not sure, read the appropriate sections before making your selection.

☒ Life sciences ☐ Behavioural & social sciences ☐ Ecological, evolutionary & environmental sciences

For a reference copy of the document with all sections, see nature.com/documents/nr-reporting-summary-flat.pdf

Life sciences study design

All studies must disclose on these points even when the disclosure is negative.

Sample size	No sample size calculation was performed, as SDS is a rare disease, these were maximum patient samples available for study. No statistical methods were used to predetermine sample size. The total amount of data collected during the experiments was the total data available for analysis in each case.
Data exclusions	Patients were chosen based on a clinical or genetic diagnosis of Shwachman-Diamond syndrome. No data were excluded from the analysis.
Replication	Independent biologic replicates performed as part of functional analysis were performed for all experiments and the numbers of replicates are described in figure legends. All attempts at replication were successful.
Randomization	This study employed data that was part of a prospective cohort study, and by design was not randomized. All patients included in this study had a clinical or genetic diagnosis in order to be included as a study participant.
Blinding	Blinding, when possible, was performed in data analysis. For colony formation assays, researcher was blinded to experimental conditions at time of colony count. Functional biochemistry studies including protein expression and polysome profiling were not blinded. Variant mutation calling was blinded to clinical outcome. All clinical data was accumulated and locked prior to statistical analysis to determine correlations of somatic mutation status and outcome. We did not blind the single cell sequencing experiments performed as these samples were selected based on prior known variants existing in the sample and selected based on the presence of these mutations in order to determine zygosity and clonality and thus not blinded. For the single cell sequencing studies, the variant allele fraction was blinded.

Reporting for specific materials, systems and methods

We require information from authors about some types of materials, experimental systems and methods used in many studies. Here, indicate whether each material, system or method listed is relevant to your study. If you are not sure if a list item applies to your research, read the appropriate section before selecting a response.

Materials & experimental systems

n/a	Involved in the study	
☐	☒	Antibodies
☐	☒	Eukaryotic cell lines
☒	☐	Palaeontology and archaeology
☒	☐	Animals and other organisms
☐	☒	Human research participants
☒	☐	Clinical data
☒	☐	Dual use research of concern

Methods

n/a	Involved in the study	
☒	☐	ChIP-seq
☐	☒	Flow cytometry
☒	☐	MRI-based neuroimaging

Antibodies

Antibodies used

Primary antibodies
1. SBDS (B1872, 1:10,000)
2. GAPDH (Cell Signaling, 2118, clone 14C10, Lot: 10, 1:1,000)
3. eIF6 (Cell Signaling, 3833, clone D16E9, Lot: 1, 1:1,000)
4. p53 (Calbiochem, OP43, clone DO-1, Lot: 3182624 1:1,000)
5. RPL3 (Abcam, ab241412, polyclonal, Lot: GR3251648-4, 1:1,000)
6. Ubiquitin (Cell Signaling, 3933, polyclonal, Lot: 6, 1:1,000)
7. V5 tag (Abcam, ab15828, polyclonal, Lot: GR3265659-1, 1:1,000)
8. V5 tag (Medical and Bio labs, M215-3, clone OZA3, Lot: 003,1:10,000)
9. Vinculin (Invitrogen, 700062, clone 42H89L44, Lot: 2090723, 1:5,000)
10. ECL anti-rabbit IgG (GE Healthcare, NA934V, Lot: 16897770, 1:10,000)
11. ECL anti-rabbit IgG (Cell Signaling, 7074, Lot: 26, 1:10,000)
12. ECL anti-mouse (GE Healthcare, NA931V, Lot: 16895895, 1:10,000)
13. Rabbit monoclonal Fibrillarin (Cell Signaling, 2639, clone C13C3, Lot: 2, 1:1000)
14. AlexaFluor 488 donkey anti-mouse (Life Technologies, A-21202, Lot: 1820538, 1:1000)

15. AlexaFluor 594 donkey anti-rabbit IgG (Fisher Scientific, A21207, Lot: 1827674, 1:1000)

Validation

Website or reference links:
1. SBDS antibody was produced by our laboratory and described in prior publication: Austin KM, Leary RJ, Shimamura A. The Shwachman-Diamond SBDS protein localizes to the nucleolus. Blood. 2005 Aug 15;106(4):1253-8. This antibody was validated by using shRNAs targeting SBDS to confirm knockdown.
2. https://www.cellsignal.com/products/primary-antibodies/gapdh-14c10-rabbit-mab/2118
3. https://www.cellsignal.com/products/primary-antibodies/eif6-d16e9-xp-rabbit-mab/3833
4. https://www.emdmillipore.com/US/en/product/Anti-p53-Ab-6-Pantropic-Mouse-mAb-DO-1,EMD_BIO-OP43
5. https://www.abcam.com/rpl3-antibody-ab241412.html
6. https://www.cellsignal.com/products/primary-antibodies/ubiquitin-antibody/3933
7. https://www.abcam.com/v5-tag-antibody-ab15828.html
8. https://www.mblintl.com/products/m215-3/
9. https://www.thermofisher.com/antibody/product/Vinculin-Antibody-clone-42H89L44-Recombinant-Monoclonal/700062
10. https://www.fishersci.com/shop/products/anti-rabbit-igg-peroxidase-linked-species-specific-whole-antibody-from-donkey-secondary-antibody-cytiva-formerly-ge-healthcare-life-sciences/p-4444576
11. https://www.cellsignal.com/products/secondary-antibodies/anti-rabbit-igg-hrp-linked-antibody/7074?bvstate=pg:2/ct:r
12. https://www.fishersci.com/shop/products/anti-mouse-igg-peroxidase-linked-species-specific-whole-antibody-from-sheep-secondary-antibody-cytiva-formerly-ge-healthcare-life-sciences/p-4444574
13. https://www.cellsignal.com/products/primary-antibodies/fibrillarin-c13c3-rabbit-mab/2639
14. https://www.thermofisher.com/antibody/product/Donkey-anti-Mouse-IgG-H-L-Highly-Cross-Adsorbed-Secondary-Antibody-Polyclonal/A-21202
15. https://www.thermofisher.com/antibody/product/Donkey-anti-Rabbit-IgG-H-L-Highly-Cross-Adsorbed-Secondary-Antibody-Polyclonal/A-21207

A summary of the manufacturer validation:
1. SBDS (not sure what to do for this)
2. GAPDH: Reacts with human, mouse, rat, monkey, bovine, pig. Tested Application: WB, IHC, IF, F.
3. eIF6: Reacts with human, mouse, rat, monkey. Tested Applications: WB, IP, IF.
4. P53: Reacts with human. Tested Applications: WB, IHC, IP.
5. RPL3: Reacts with mouse, human. Tested Applications: WB, IP.
6. Ubiquitin: Reacts with all species expected. Tested Applications: WB, IHC.
7. V5 tag (abcam): Reacts with human. Tested Applications: ChIP, WB.
8. V5 tag (MBL): Tested Applications: FCM, ICC, IP, WB.
9. Vinculin: Reacts with human, mouse. Tested Applications: Flow, ICC, IF, WB.
10. ECL anti-rabbit IgG: Secondary antibody. Reacts with rabbit IgG. Suitable for WB, ELISA, IHC.
11. ECL anti-rabbit IgG: Secondary antibody. Reacts with heavy and light chains of rabbit IgG. Suitable and validated WB applications.
12. ECL anti-mouse: Secondary antibody. Reacts with mouse IgG. Suitable and validated for WB, ELISA, IHC.
13. Fibrillarin: Reacts with human, mouse, rat, mink. Tested Applications: WB, IF.
14. Donkey anti-mouse: Secondary antibody. Reacts with heavy and light chains of mouse IgG. Tested Applications: ICC, IF, IHC.
15. Donkey anti-rabbit: Secondary antibody. Reacts with heavy and light chains of rabbit IgG. Tested Applications: Flow, ICC, IF, IHC.
In case of anti-SBDS antibody, this antibody is previously published (and was previously validated with multiple shRNA hairpins targeting SBDS to demonstrate specificity.

Eukaryotic cell lines

Policy information about cell lines

Cell line source(s)

K562 and K562-TP53 corrected were gifts from Dr. Ben Ebert's laboratory. 293T and HT1080 cells were gifts from Dr. David William's laboratory. Patient-derived fibroblasts, these were generated by our laboratory.

Authentication

The K562 cell lines used were authenticated by short tandem repeat (STR) testing through Dana Farber core facility. The 293T and HT1080 cells were used in viral production and for viral titer, respectively and were not authenticated. The patient derived fibroblasts from SDS patients were not authenticated, but were verified to have SBDS deficient protein expression by western blotting.

Mycoplasma contamination

All cell lines used in this manuscript were tested for mycoplasma and were mycoplasma negative.

Commonly misidentified lines
(See ICLAC register)

No commonly misidentified eukaryotic cell lines were used in this manuscript.

Human research participants

Policy information about studies involving human research participants

Population characteristics	The age, gender and genotype information are published alongside the manuscript in Table 1.
Recruitment	All patients enrolled in the North American SDS Registry were eligible for the study. Those containing marrow samples were analyzed without exclusion criteria. Not all SDS patients participate in the registry. If there are biologic correlates related to participation in the registry, this is a potential source of bias.
Ethics oversight	The IRBs at Boston Children's Hospital and Cincinnati Children's Hospital approved the use of human research participants in this study.

Note that full information on the approval of the study protocol must also be provided in the manuscript.

Flow Cytometry

Plots

Confirm that:

- [x] The axis labels state the marker and fluorochrome used (e.g. CD4-FITC).
- [x] The axis scales are clearly visible. Include numbers along axes only for bottom left plot of group (a 'group' is an analysis of identical markers).
- [x] All plots are contour plots with outliers or pseudocolor plots.
- [x] A numerical value for number of cells or percentage (with statistics) is provided.

Methodology

Sample preparation	Sample preparation for each experiment in which flow cytometry is used for sorting or analysis is described in detail in the materials and methods section. These methods sections include colony formation assays, OP-PURO incorporation and growth competition assays.
Instrument	Fortessa HTS flow cytometer (BD biosciences) was used for growth competition and OP-PURO incorporation.
Software	The flow cytometry data was collected using FACSdiva software and FCS files were then analyzed using FlowJo version 10.
Cell population abundance	For CD34 experiments, post sort fractions sorted on GFP or RFP positivity as compared to an untransduced population. Post sort fractions were 100% either GFP+, RFP+ or double positive as shown in gating strategy in supplementary figure.
Gating strategy	For each flow-based experiment, live cells were gated based on FSC/SSC to delineate from debris and dying cells. Subsequently, doublets were excluded by plotting FSC-H vs FSC-A. Following this, fluorophore positivity was determined by comparing to untransduced cells. Gating strategy for each experiment is shown in supplementary figure.

- [x] Tick this box to confirm that a figure exemplifying the gating strategy is provided in the Supplementary Information.
